# JMJD1B Demethylates H4R3me2s and H3K9me2 to Facilitate Gene Expression for Development of Hematopoietic Stem and Progenitor Cells

**DOI:** 10.1016/j.celrep.2018.03.051

**Published:** 2018-04-10

**Authors:** Sihui Li, Shafat Ali, Xiaotao Duan, Songbai Liu, Juan Du, Changwei Liu, Huifang Dai, Mian Zhou, Lina Zhou, Lu Yang, Peiguo Chu, Ling Li, Ravi Bhatia, Dustin E. Schones, Xiwei Wu, Hong Xu, Yuejin Hua, Zhigang Guo, Yanzhong Yang, Li Zheng, Binghui Shen

**Affiliations:** 1 College of Life Sciences, Zhejiang University, Hangzhou, Zhejiang, China; 2 Departments of Cancer Genetics and Epigenetics, Beckman Research Institute, City of Hope, Duarte, CA, USA; 3 State Key Laboratory of Toxicology and Medical Countermeasures, Beijing Institute of Pharmacology and Toxicology, Beijing, China; 4 Department of Diabetes Complications & Metabolism, Beckman Research Institute, City of Hope, Duarte, CA, USA; 5 Department of Molecular and Cellular Biology, Beckman Research Institute, City of Hope, Duarte, CA, USA; 6 Department of Pathology, Beckman Research Institute, City of Hope, Duarte, CA, USA; 7 Department of Hematologic Malignancy Translational Science, Beckman Research Institute, City of Hope, Duarte, CA, USA; 8 Colleges of Life Sciences and Agriculture and Biotechnology, Zhejiang University, Hangzhou, Zhejiang, China; 9 College of Life Sciences, Nanjing Normal University, Nanjing, Jiangsu, China

## Abstract

The arginine methylation status of histones dynamically changes during many cellular processes, including hematopoietic stem/progenitor cell (HSPC) development. The arginine methyltransferases and the readers that transduce the histone codes have been defined. However, whether arginine demethylation actively occurs in cells and what enzyme demethylates the methylarginine residues during various cellular processes are unknown. We report that JMJD1B, previously identified as a lysine demethylase for H3K9me2, mediates arginine demethylation of H4R3me2s and its intermediate, H4R3me1. We show that demethylation of H4R3me2s and H3K9me2s in promoter regions is correlated with active gene expression. Furthermore, knockout of JMJD1B blocks demethylation of H4R3me2s and/or H3K9me2 at distinct clusters of genes and impairs the activation of genes important for HSPC differentiation and development. Consequently, JMJD1B^−/−^ mice show defects in hematopoiesis. Altogether, our study demonstrates that demethylase-mediated active arginine demethylation process exists in eukaryotes and that JMJD1B demethylates both H4R3me2s and H3K9me2 for epigenetic programming during hematopoiesis.

## In Brief

Li et al. identify the arginine demethylase (RDM) activity of JMJD1B, a known lysine demethylase (KDM). They reveal that JMJD1B actively mediates demethylation of histone markers H4R3me2s and H3K9me2 in hematopoietic stem/progenitor cells (HSPCs).

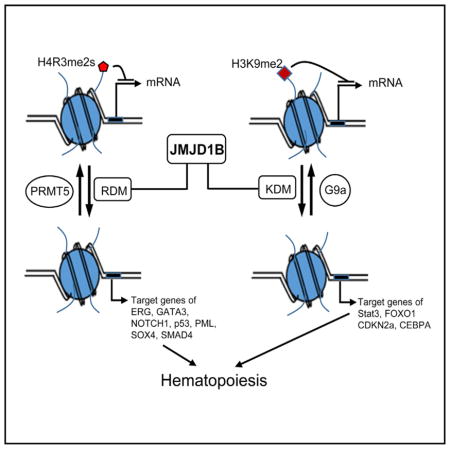

## INTRODUCTION

The arginine methylation status of core histones dynamically changes during many essential cellular processes, particularly during embryonic and hematopoietic stem cell (HSC) development (Bedford and Clarke, 2009; Blanc and Richard, 2017; Chen et al., 2011; Yang and Bedford, 2013). There are nine arginine methyltransferase (PRMT) genes encoded in the human genome, and their protein products catalyze the methylation reaction in three different forms: ω-NG-monomethylarginine (MMA, me1), ω-NG, NG-asymmetric dimethylarginine (ADMA, me2a), and ω-NG, N′G-symmetric dimethylarginine (SDMA, me2s) (Fuhrmann et al., 2015; Yang and Bedford, 2013). These modifications often serve as docking sites for Tudor domain-containing effector molecules, which transduce post-translational modification signals to produce biological outcomes (Chen et al., 2011). Accumulating evidence suggests that arginine methylation is dynamically regulated in cells during transcription (Le Romancer et al., 2008; Sims et al., 2011), replication (Guo et al., 2010), DNA damage response (Jansson et al., 2008), and cell cycle progression (Guo et al., 2010; Yu et al., 2009). Accordant with this, dysregulation of histone arginine methylation has been linked to many human diseases, including neurological disorders, autoimmunity, and cancer (Yang and Bedford, 2013).

Although arginine methyltransferases have been identified and their function in cells has been well documented (Fuhrmann et al., 2015; Yang and Bedford, 2013), arginine demethylases have not yet been identified. JMJD6 (JmjC domain containing 6) was previously reported as a putative arginine demethylase for ADMA and SDMA histone substrates (Chang et al., 2007). However, additional structural and functional studies showed that lysine, rather than arginine, is the substrate of JMJD6 (Mantri et al., 2010; Webby et al., 2009). More recently, a study showed that certain lysine demethylases possess arginine demethylase activity on methylated histone peptide model substrates (Walport et al., 2016). However, an arginine demethylase with a biological role has not been discovered *in vivo* (Blanc and Richard, 2017; Greer and Shi, 2012; Yang and Bedford, 2013). Identification of the histone arginine demethylase will not only fill a fundamental gap in our understanding of the field of epigenetics but also provide additional targets for therapeutic regimens.

Here, we report that JMJD1B possesses arginine demethylase activity on H4R3me2s. We establish that the demethylation of H4R3me2s in promoter and gene body regions is inversely correlated with gene expression in hematopoietic stem/progenitor cells (HSPCs). Furthermore, we observed that knockout of JMJD1B causes defects in demethylation of H4R3me2s, leading to downregulation of genes important for hematopoietic cell differentiation and development. Consequently, JMJD1B^−/−^ mice display defective hematopoiesis, showing moderate anemia and remarkable leukocytosis phenotypes. Altogether, our study demonstrates that arginine demethylases exist in cellular systems and that JMJD1B demethylates H4R3me2s for proper epigenetic programming during development.

## RESULTS

### JMJD1B Protein Possesses Both Lysine and Arginine Demethylase Activities

**Figure 1:**
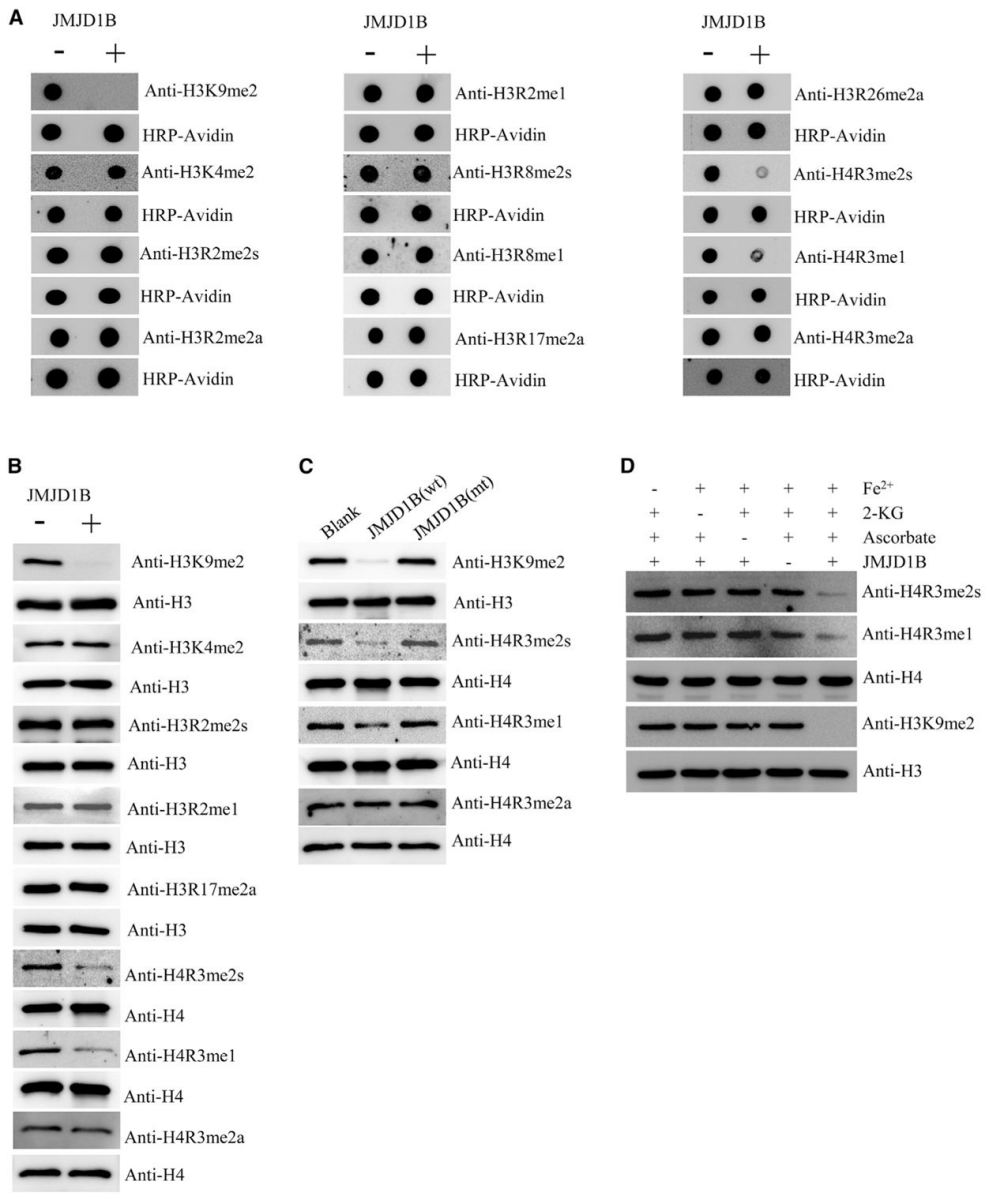
JMJD1B Protein Possesses Both Lysine and Arginine Demethylase Activities *In Vitro* (A) Synthetic histone tails (22–25 amino acid residues, 18 μM) were incubated in a demethylation buffer (50 mM HEPES-KOH [pH 7.5], 1 mM 2-KG, 2 mM ascorbate, 1 mM TCEP, 500 μM [NH_4_]_2_Fe[SO_4_]_2_·6H_2_O) in the absence (−) or presence (+) of purified recombinant JMJD1B (0.25 μM) at 37°C for 2 hr and analyzed using dot blot with indicated antibodies. Blots were stripped and reprobed for HRP-avidin as loading control. (B) Bulk histones (5 μg) were incubated in a demethylation buffer as in (A) in the absence (−) or presence (+) of purified recombinant JMJD1B (0.25 μM) at 37°C for 2 hr and analyzed using western blotting. Blots were stripped and reprobed for total levels of H3 and H4 as loading controls. (C) Bulk histones (5 μg) were incubated in absence (Blank) or presence of WT JMJD1B (wt; 0.25 μM) or a catalytically inactive mutant (mut; JMJD1B [H1560A/D1562A/H1689A], 0.25 μM) at 37°C for 2 and 4 hr and analyzed using western blotting. The total level of H4 was used as an internal loading control. (D) Three cofactors, Fe^2+^, 2-KG, and ascorbate, are required for the full enzyme activity to demethylate both H4R3me2s and H3K9me2. The same reaction conditions as in (B) and (C) were used. See also Table S1 and Figure S1.

JMJD1B, which is a typical JmjC domain-containing protein, was previously characterized as one of the lysine demethylases targeting H3K9 dimethylation (Kim et al., 2012; Mikhaleva et al., 2011). We recently found that flap endonuclease 1 (FEN1), which undergoes SDMA at the R192 residue in S phase or in response to DNA damage (Guo et al., 2010), is in complex with JMJD1B. This prompted us to hypothesize that JMJD1B may be an arginine demethylase acting on both histone and non-histone proteins. To determine whether JMJD1B is a histone arginine demethylase, we expressed and purified FLAG-tagged full-length wild-type (WT) JMJD1B (isoform 1) or a catalytically inactive JMJD1B mutant (mut), which carries mutations in the conserved cofactor (Fe^2+^) binding site (H1560A/D1562A/H1689A) (purity > 90%; Figure S1A). We validated the JMJD1B lysine demethylase activity toward H3K9me2 (Figure 1A) and also evaluated arginine demethylase activity toward H3 and H4, which are known methylation targets (Huang et al., 2014). With the synthetic histone peptides and antibodies (Table S1) against a specific form and histone modification, whose specificity was verified (Figure S1B), we conducted dot-blot-based *in vitro* demethylation assays in the absence or presence of recombinant JMJD1B. JMJD1B had no activity toward the synthesized lysine-methylated and arginine-methylated histone peptides, including H3R2me1, H3R2me2a, H3R2me2s, H3R8me1, H3R8me2s, H3R17me2a, H3R26me2a, H4R3me2a, and H3K4me2. In contrast, JMJD1B exhibited demethylase activity specifically toward H3K9me2, H4R3me1, and H4R3me2s peptides (Figure 1A). We next performed *in vitro* demethylation assays using bulk histones purified from HEK293T cells as substrates and confirmed that JMJD1B preferentially demethylated H3K9me2, H4R3me1, and H4R3me2s (Figure 1B). Mutant JMJD1B showed no activity toward H3K9me2, H4R3me2s, or H4R3me1 (Figure 1C). For optimal activity, lysine demethylases require three cofactors: Fe^2+^, α-ketoglutarate, and ascorbate (Klose et al., 2006; Shi et al., 2004). These three cofactors were also required for the arginine demethylase activity (Figure 1D). Together, these data support a model in which JMJD1B demethylates both lysine (H3K9me2) and arginine (H4R3me2s and H4R3me1) residues.

**Figure 2:**
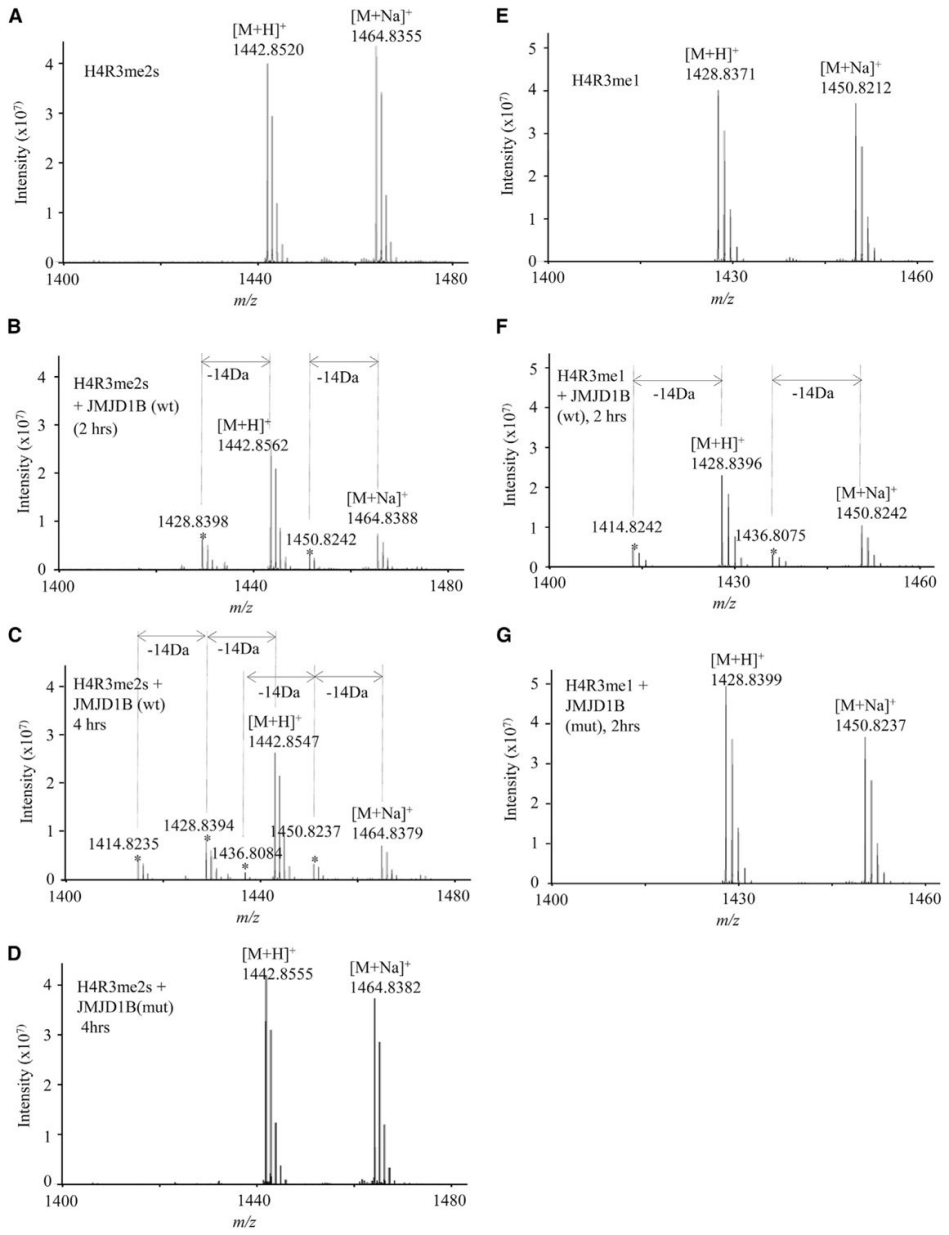
Mass Spectrometry Confirms that JMJD1B Demethylates H4R3me2s and H4R3me1 Peptide Model Substrates (A–D) Representative mass spectra for H4R3me2s demethylation by JMJD1B. The H4R3me2s 16 aa peptide substrate (34 μM) was incubated with buffer only (α-ketoglutarate [2-KG, 1 mM], ascorbate [2 mM], and Fe^2+^ [500 μM]) for 4 hr (A), with wild-type enzyme (JMJD1B [wt]; 0.5 μM) at 37°C for 2 hr (B) and 4 hr (C), or with inactive mutant enzyme for 4 hr (JMJD1B [mutant]; 0.5 μM) (D). Asterisk indicates products from the H4R3me2s peptide (B and C). (E–G) Representative mass spectra for H4R3me1 demethylation by JMJD1B. The H4R3me1 peptide substrate (34 μM) was incubated with demethylation buffer (E), 0.5 μM JMJD1B (wt) (F), or 0.5 μM JMJD1B (mutant) (G) at 37°C for 2 hr. Asterisk indicates the demethylation products. See also Table S2 and Figure S2.

To confirm the arginine demethylase activity of JMJD1B, we conducted *in vitro* demethylation using purified WT or mutant JMJD1B, and used Fourier-transform ion cyclotron resonance (FT-ICR) mass spectrometry to analyze the reaction products. Synthetic histone H4 tail peptide H4R3me1, H4R3me2s, and H4R3me2a were used as arginine demethylation substrates (Table S2). The known JMJD1B lysine demethylation substrate, H3K9me2, was used as a positive control (Figures S2A–S2C). In the absence of JMJD1B, we observed peaks in the H4R3me2s substrate at 1,443 and 1,465 mass-to-charge ratio (m/z), which correspond to the H4R3me2s peptide molecular ion plus a proton ([M+H]^+^) and the molecular ion plus Na^+^ ([M+Na]^+^), respectively (Figure 2A). After incubating JMJD1B with the H4R3me2s peptide substrate for 2 hr, the intensity of these two peaks was reduced, and two new peaks appeared, which were shifted 14 Da from the 1,443 ([M+H]^+^) peak and the 1,465 ([M+Na]^+^) peak, corresponding to the molecular ions of H4R3me1 (Figure 2B). As we increased the incubation time to 4 hr, we observed an additional peak with a shift of 28 Da from the original [M+H]^+^ or [M+Na]^+^ peak, which corresponds to the molecular ion of the non-modified H4 tail peptide (Figure 2C). In contrast, incubating mutant JMJD1B with the H4R3me2s substrate did not resulted in any peak shift (Figure 2D). Similarly, in the absence of JMJD1B, we observed [M+H]^+^ and [M+Na]^+^ peaks in the H4R3me1 substrate (Figure 2E). Addition of WT JMJD1B but not mutant JMJD1B resulted in peaks that were shifted 14 Da from the original [M+H]^+^ and [M+Na]^+^ peaks (Figures 2F and 2G), corresponding to the molecular ion of the non-modified H4 tail peptide. The mass spectrometry revealed no arginine demethylation products when WT or mutant JMJD1B was incubated with the H4R3me2a substrate (Figures S2D–S2F). Altogether, these biochemical data indicate that JMJD1B specifically demethylates H4R3me2s but not H4R3me2a.

### The Rate of JMJD1B Arginine Demethylase Activity Is Similar to That of Its Lysine Demethylase Activity *In Vitro*

**Figure 3:**
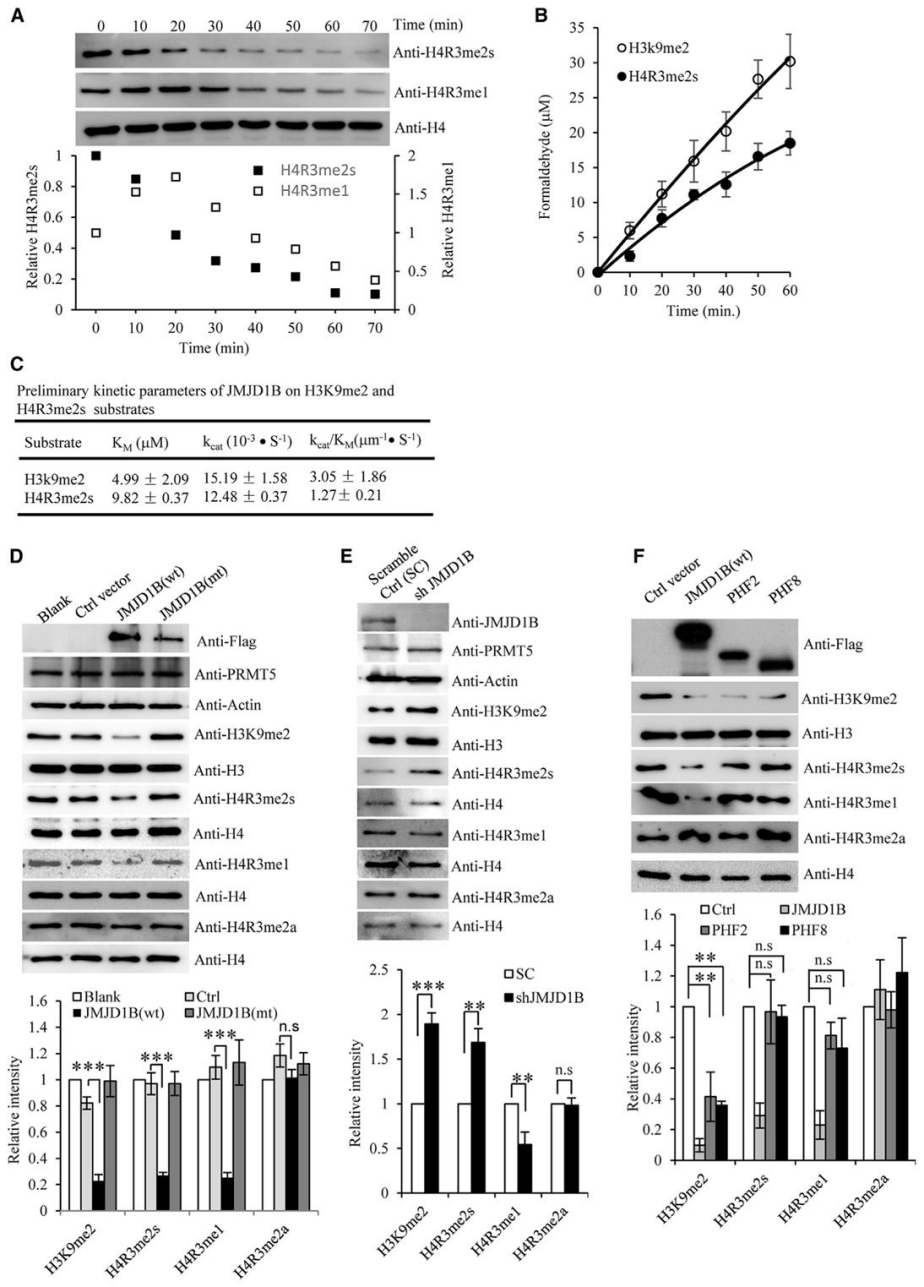
JMJD1B Demethylates Histone H4R3me2s and H3K9me2 *In Vitro* and in Cells (A) Time dependence of JMJD1B histone arginine demethylase activity. Bulk histones (5 μg) were incubated with JMJD1B (0.5 μM) in the demethylation at 37°C for 0–70 min, as indicated, and analyzed by western blotting. Top: representative western blotting images; bottom: semi-quantification of H4R3me2s and H4R3me1 levels. The H4R3me1 and H4R3me2s levels were normalized with corresponding total H4 levels. The normalized H4R3me1 and H4R3me2s levels at 0 hr were arbitrarily set as 1. The relative levels of H4R3me1 and H4R3me2s at other time points were calculated relative to the value at 0 min. Values are the average of two independent assays. (B) Time-dependent formaldehyde release in JMJD1B-mediated demethylation of H4R3me2s and H3K9me2 peptide substrates. The H4R3me2s or H3K9me2 peptide substrates (50 μM) were incubated with JMJD1B (0.5 μM) in the demethylation buffer at 37°C, and the release of formaldehyde was measured at 0, 10, 20, 30, 40, 50, and 60 min. Values are mean ± SEM of three independent assays. (C) Preliminary kinetic parameters of JMJD1B-mediated demethylation of H4R3me2s or H3K9me2. The formaldehyde release assay on different substrate concentrations was used to generate *K*_m_, *k*_cat_, and *k*_cat_/*K*_m_. Values are mean ± SEM of three independent assays. (D–F) Alteration of JMJD1B expression level corresponds to H4R3me2s and H4R3me1 demethylation status in HEK293T cells. (D) JMJD1B was expressed in HEK293T cells as a FLAG fusion protein, and histones were analyzed by western blotting with antibodies against the indicated modifications. Cells with no transfection (Blank) or transfected with an empty vector (Ctrl) were used as controls. (E) Stable JMJD1B-knockdown cells (Sh-JMJD1B) were lysed and immuno-blotted with the indicated antibodies. (F) JMJD1B, PHF2, and PHF8 were expressed in HEK293T cells, and histones were analyzed by western blotting with antibodies against the indicated modifications. Histone H3 and H4 were used as a loading control in all panels. In each panel, the upper section shows the representative western blotting images, and the lower section shows the semi-quantification of western blotting (i.e., the relative intensities of H4R3me2s, H4R3me1, and H3K9me2 bands). Band intensity was normalized to corresponding loading control (histone H3 or H4). The normalized H4R3me2s, H4R3me1, or H3K9me2 levels in the cells with no transfection, or transfected with control short hairpin RNA (shRNA) (SC) or empty vector were arbitrarily set as 1 in (D), (E), and (F), respectively. The relative intensity was calculated by comparing the normalized band intensity of each sample to that of the control. Values are means ± SEM of three independent assays. *p < 0.05, **p < 0.01, and ***p < 0.0001 (Student’s t test). See also Figure S1.

We determined the kinetics of JMJD1B-mediated arginine demethylation of H4R3me2s. We first evaluated the intermediates of the JMJD1B-mediated arginine demethylation reaction and found that the amount of H4R3me2s decreased with time (Figure 3A). In contrast, the H4R3me1 level first increased, and after reaching its maximum at 20 min, it started to decrease. This is consistent with a model in which H4R3me2s is the substrate of JMJD1B, and H4R3me1 is an intermediate in the JMJD1B-mediated demethylation of H4R3me2s. The rate of release of the methyl group from H4R3me2s was further measured by the release of formaldehyde, which showed that the JMJD1B-catalyzed H4R3me2s demethylation rate was slightly lower than the demethylation rate for H3K9me2 (Figure 3B). The preliminary kinetic parameters suggested that although purified recombinant JMJD1B has a similar turnover rate on the H3K9me2 and H4R3me2s peptide substrates (i.e., *k*_cat_ = 15.19 and 12.48), it had higher affinity for the H3K9me2 peptide substrate than the H4R3me2s peptide substrate (i.e., *K*_m_ = 4.99 μM versus 9.82 μM; Figure 3C).

### Cellular Level of JMJD1B Corresponds to H4R3me2s and H3K9me2 Methylation Status

To determine if JMJD1B mediates arginine demethylation of H4R3me2s and H4R3me1 in cells, we overexpressed WT or mutant JMJD1B in HEK293T cells and measured H4R3me2s and H4R3me1 levels. As anticipated, overexpression of WT, but not mutant, JMJD1B significantly reduced the H3K9me2, H4R3me2s, and H4R3me1 levels, compared with parental control cells (Figure 3D). However, overexpression of JMJD1B did not affect the H4R3me2a level (Figure 3D). Conversely, knockdown of JMJD1B (Figure S1C) resulted in H3K9me2 and H4R3me2s accumulation in the cells (Figure 3E). The H4R3me1 level was reduced in JMJD1B-knockdown cells (Figure 3E), consistent with a model in which H4R3me1 is an intermediate of JMJD1B-mediated H4R3me2s demethylation. To demonstrate that JMJD1B does not affect H4R3me2s via regulation of PRMT5, we assessed the level of PRMT5 in JMJD1B-overexpressing or JMJD1B-knockdown cells; we noted little change compared with control cells (Figures 3D and 3E). To determine whether other H3K9me2 demethylases could also catalyze arginine demethylation, we overexpressed PHF2 and PHF8, which have been reported to show H3K9me2 demethylase activity (Lee et al., 2014; Qi et al., 2010). Overexpression of PHF2 or PHF8 reduced the level of H3K9me2, as expected, but did not change the level of H4R3me2s, H4R3me1, or H4R3me2a (Figure 3F). This is consistent with the possibility that JMJD1B is a unique H4R3me2s and H4R3me1 demethylase.

### Highly Expressed JMJD1B Maintains the Demethylation Status of H3K9me2 and H4R3me2s in HSPCs

**Figure 4:**
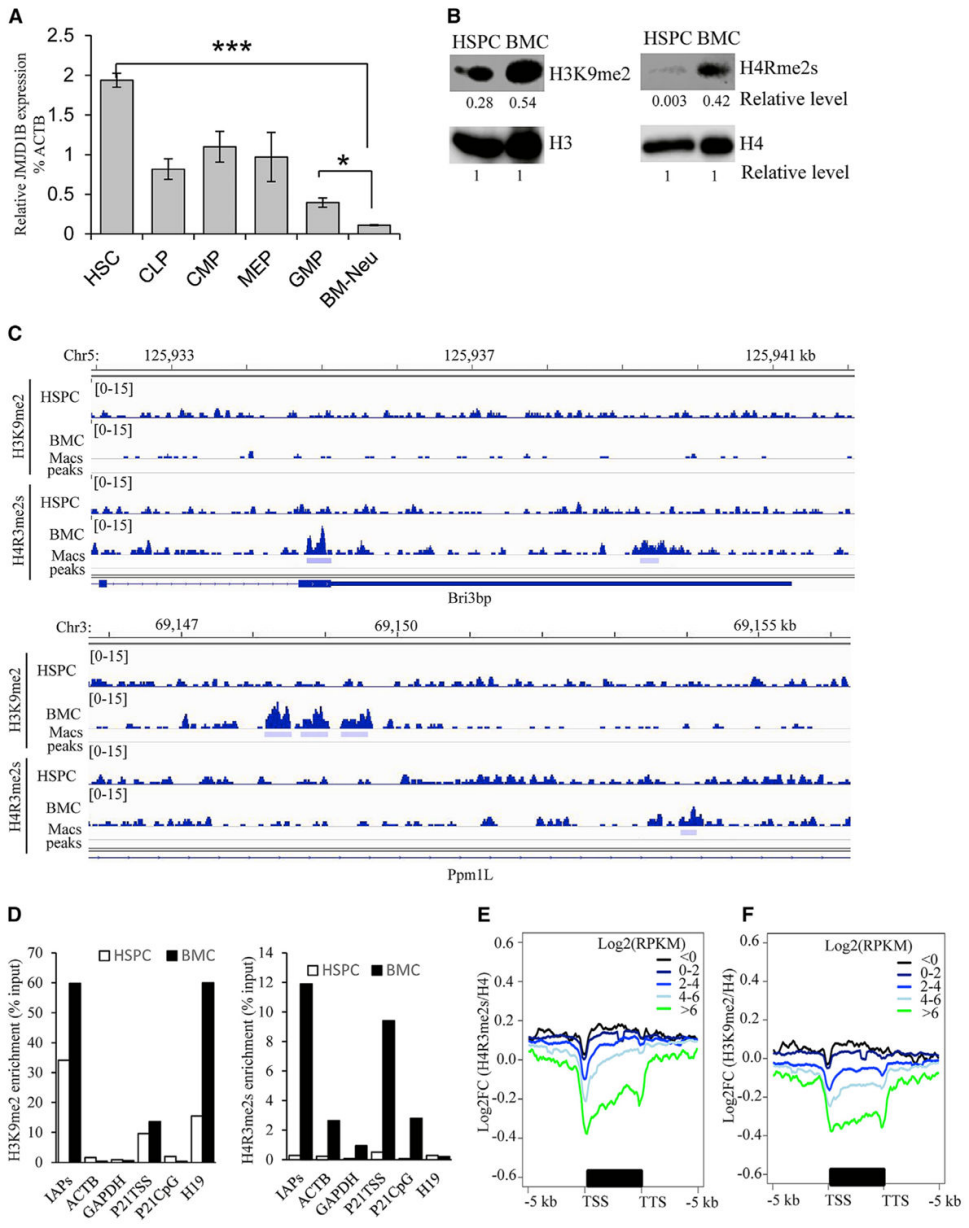
Elevated JMJD1B Expression Maintains the Demethylation Status of H3K9me2 and H4R3me2s in HSPCs (A) JMJD1B gene expression in hematopoietic stem cells and various progenitors, BMCs, and blood cells. HSC, hematopoietic stem cell; CLP, common lymphoid progenitor; CMP, common myeloid progenitor; MEP, megakaryocyte-erythrocyte progenitor; GMP, granulocyte-monocytes progenitor; BM-Neu, bone marrow neutrophils. JMJD1B and the house keeping gene β-actin (ATCB) were measured using real-time RT-PCR. The relative levels of JMJD1B mRNA were calculated by comparing it with the level corresponding β-actin mRNA, which is arbitrarily set as 100, in each type of cell. Values are mean ± SEM of three mice. *p < 0.05, **p < 0.01, and ***p < 0.001 (Student’s t test). (B) Total chromatin-associated histone H3 and H4, H4R3me2s, and H3K9me2 in WT HSPCs and BMCs were detected by western blot. The intensity of each band was semi-quantified using ImageJ. The intensity of histone H3 or H4 for each sample was arbitrarily set as 1, and the relative level of H4R3me2s or H3K9me2 in HSPCs or BMCs was calculated by comparing their intensity with corresponding H3 or H4 control. (C) IGV views of ChIP-seq show representative enriched H3K9me2 and H4R3me2s peaks (p < 0.0001) in BMCs but not in HSPCs. (D) ChIP-qPCR for verifying H4R3me2s and H3K9me2 occupancy at selected gene loci. The ChIPed signals were normalized with corresponding input and were expressed as percentage of input. Values are the mean of three ChIP experiments. (E and F) Aggregate plot of H4R3me2s (E) or H3K9m2 (F) abundance (Log_2_FC[ChIP/ChIP H4]) and gene expression levels (log[RPKM+0.1]) for WT HSPCs. TSS, transcription start site; TTS, transcription termination site. See also Table S3.

Human JMJD1B is highly expressed in hematopoietic cells, retinal tissue, and pineal glands (http://biogps.org), suggesting that it plays a role in development and/or maintenance of proper cellular functions in these cells. Similar to human hematopoietic cells, mouse HSPCs also expressed elevated JMJD1B (Figure 4A) compared with other cell types. In contrast, JMJD1B expression reduced significantly in mature bone marrow cells (BMCs) such as bone marrow neutrophils (Figure 4A). Consistently, HSPCs had relatively low levels of H4R3me2s and H3K9me2 compared with BMCs (Figure 4B).

We then determined if high JMJD1B expression is associated with low H4R3me2s and H3K9me2 density in HSPCs. We performed MACS2 analysis of native chromatin immunoprecipitation-DNA sequencing (ChIP-seq) on H3K9me2 and H4R3me2s and identified 9,822 and 622 H3K9me2 and H4R3me2s peaks (p < 0.0001) in BMCs (Figure 4C). In contrast, H3K9me2 and H4R3me2s had only 1 and 28 significantly enriched peaks in HSPCs, respectively (Figure 4C). Real-time PCR on representative genomic loci confirmed that in many genomic loci, H4R3me2s and H3K9me2 occupancy in HSPCs was much lower than in BMCs (Figure 4D). These findings suggest that during hematopoiesis, H3K9me2s and H4R3me2s histone markers are not established at most loci in HSPCs. This is consistent with previous findings that the H3K9me2 marker is not established in human HSCs (Chen et al., 2012; Schones et al., 2014). In addition, both PRMT5 and G9a (EHMT2), the methyltransferases for H4R3me2s and H3K9me2 respectively were actively expressed (log_2_RPKM [reads per kilobase of transcript per million mapped reads] > 4). Thus, the non-establishment status of H4R3me2s and H3K9me2 in HSPCs is likely due to the activity of demethylases, rather than low levels of methyltransferases.

Even though the currently available software was not able to call peaks from the ChIP-seq data in HSPCs, there are significant differences in the H3K9me2 and H4R3me2s occupancy densities across the genome. To determine the impact of H3K9me2 and H4R3me2s density on gene expression in HSPCs, we conducted RNA sequencing (RNA-seq) and analyzed the relationship between the density of these two histone markers and the levels of gene expression. We categorized genes across the genome into clusters on the basis of gene expression levels and compared them with the H4R3me2s and H3K9me2 occupancy density levels across the gene bodies (transcription start site [TSS] ± 5 kb). The aggregate plots showed an inverse correlation of H4R3me2s and H3K9me2 occupancy and gene expression levels: the reduction of H4R3me2s or H3K9me2 occupancy was associated with an increase in gene expression (Figures 4E and 4F). This suggests that maintaining the demethylation status of the suppressive markers H4R3me2s and H3K9me2 is important for induction of gene expression in HSPCs.

### JMJD1B Demethylates H4R3me2s and H3K9me2 to Regulate Genes Important for HSPC Survival, Proliferation, and Differentiation

**Figure 5:**
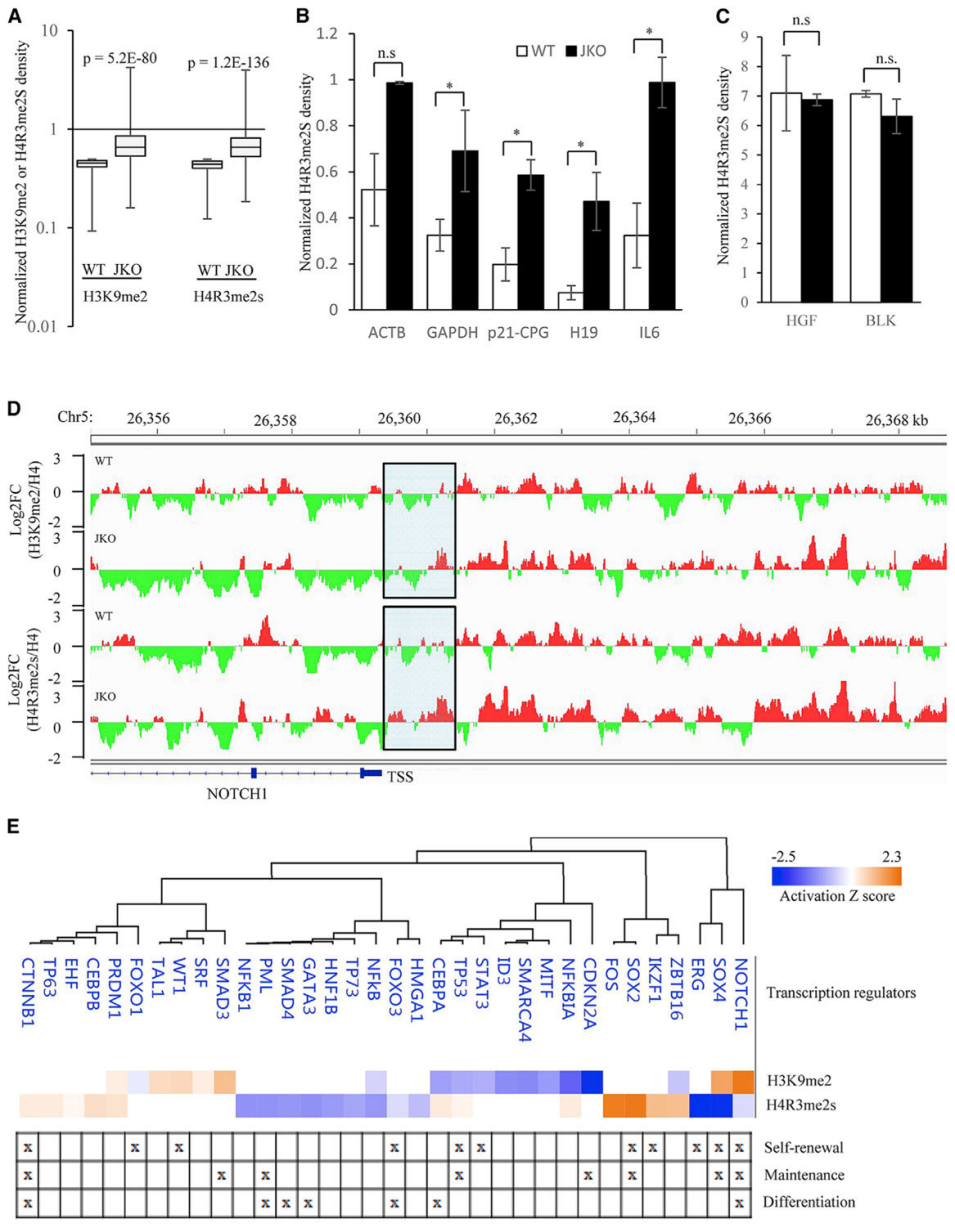
JMJD1B Maintains Demethylation Status of H4R3me2s and H3K9me2 at the Promoter Regions of Distinct Clusters of Genes in HSPCs for Hematopoiesis (A) Box-and-whisker plots of H3K9me2/H4 and H4R3me2s/H4 signal changes between the mutant and WT HSPCs at the promoter regions of genes that are depleted of H3K9me2 or H4R3me2s in the WT HSPCs. The depleted genes are defined as those with log_2_FC (H3K9me2/H4) or log_2_FC (H4R3me2s/H4) ≤ −1 in the WT HSPCs. (B and C) ChIP-qPCR measurement of H4R3me2s occupancy at H4R3me2s-depleted genes (B) and the non-depleted genes (C) in WT and JMJD1B^−/−^ cells. The ChIPed H4R3me2s signal was normalized with the corresponding ChIPed H4 signal and was expressed as percentage of ChIPed H4. Values are mean ± SEM of three ChIP experiments. *p < 0.05 (Student’s t test). (D) Representative IGV view of the H4R3me2s and H3K9me2 ChIP signals normalized to the H4 ChIP signal in the promoter region of a depleted gene (e.g., NOTCH1 gene). The promoter region was defined as the region from the transcription start site (TSS) to 1 kb upstream of the TSS (highlighted). The depleted or enriched regions are specified by green or red color. (E) IPA upstream analysis predicts different transcription factors corresponding to the overlapped genes as defined in (D). The transcription factors with p < 0.01 and a calculated *Z* score are shown. The roles of different transcription factors in the self-renewal, maintenance, and differentiation are specified. See also Figures S3 and S4.

A key question is whether JMJD1B plays an active role in maintaining H4R3me2s and H3K9me2 markers at low levels for gene expression in HSPCs. To this aim, we established JMJD1B knockout (JMJD1B^−/−^; JKO) mice (Figure S3), using validated JMJD1B^−/−^ embryonic stem cells (129P2 genetic background) from the European Mouse Mutant Cell Repository (EuMMCR). We performed H4R3me2s and H3K9me2 ChIP-seq experiments using HSPCs that were purified from the bone marrow of age-matched WT and JMJD1B^−/−^ littermates. We then analyzed the H4R3me2s and H3K9me2 ChIP signals at the promoter region (TSS −1 kb upstream of TSS), and normalized them to corresponding H4 ChIP signals at the same region across the genome in the WT and JMJD1B^−/−^ HSPCs. On the basis of the status of H3K9me2 or H4Rme2s signals relative to H4 in WT HSPCs, we divided the genes across the genome into four groups: the 2-fold or more depletion group (log_2_ fold change [FC] [H3K9me2 or H4R3me2s over H4] ≤ −1), the 1- to 2-fold depletion group (−1 < log_2_FC [H3K9me2 or H4R3me2s over H4] ≤ 0), the 1- to 2-fold enrichment group (0 < log_2_FC [H3K9me2 or H4R3me2s over H4] ≤ 1), and the 2-fold or more enrichment group (0 < log_2_FC [H3K9me2 or H4R3me2s over H4]). We found that JMJD1B knockout led to a significant increase (FC of JMJD1B^−/−^ over WT ≥ 1.5, p < 0.05) in the mean density of H4R3me2s and H3K9me2 in the genes of the 2-fold or more depletion group (Figure 5A). qPCR analysis confirmed that JMJD1B knockout significantly increased the H4R3me2s signals at H4R3m2s-depleted genes (Figure 5B) but not in the non-depleted genes (Figure 5C).

Figure 5D shows a representative gene, NOTCH1, with depletion (in green) of H3K9me2 and H4R3me2 at the promoter region in WT HSPCs; in contrast, JMJD1B knockout resulted in significant gain (in red) of H4R3me2s and H3K9me2 signals. Similar to the NOTCH1 gene, 2,421 and 3,001 genes, respectively, had significant H3K9me2 or H4R3me2s occupancy density increases, which we defined as JMJD1B knockout ChIP reads ≥ 15 and the FC of JMJD1B^−/−^ over WT ≥ 1.5 at the promoter regions. The identified genes with gains in H4R3me2s and/or H3K9me2 ChIP signals could be categorized into three types on the basis of the methylation markers in JMJD1B^−/−^ versus WT HSPCs: (1) 967 genes showed a ≥1.5-fold increase in both H4R3me2s and H3K9me2 density, (2) 1,454 genes had a ≥1.5-fold increase in H3K9me2 but not H4R3me2s, and (3) 2,034 genes displayed a ≥1.5-fold increase in H4R3me2s but not H3K9me2. Therefore, these two genetic markers influence gene expression distinctively. Of note, JMJD1B^−/−^ did not change the level of H3K9me2/H4R3me2s at PRMTs or G9a loci or methyltransferase gene expression levels (Figures S4A and S4B). Thus, the elevation of H4R3me2s or H3K9me2 level in the JMJD1B^−/−^ HSPCs is likely not due to change of protein methyl transferase activities.

We sought to define the genes, corresponding pathways, and cellular functions for hematopoiesis that are controlled by JMJD1B-mediated demethylation of H4R3me2s and H3K9me2 in HSPCs for hematopoiesis. RNA-seq analysis revealed that 937 genes with statistically decreased expression (p < 0.05) and 444 genes with statistically increased expression (p < 0.05) in JMJD1B^−/−^ HSPCs, compared with the WT (Figures S4C–S4E). We characterized the intersection of genes with increases in H4R3me2s or H3K9me2 markers and the genes with altered expression (downregulation or upregulation) in JMJD1B^−/−^ HSPCs. A total of 140 and 99 genes, respectively, of relatively high H4R3me2s and H3K9me2 density were down-regulated genes (Figure S4D). Fifty-six and 40 genes, respectively, of relatively high H4R3me2s or H3K9me2 genes were upregulated genes in JMJD1B^−/−^ HSPCs (Figure S4E). Considering H4R3me2s and H3K9me2 are repressive markers, it is likely that H4R3me2s and H3K9me2 might inhibit the expression of inhibitory transcription regulators, leading to upregulation of the target genes of the transcription repressors. In addition, JMJD1B knockout might also affect the activity of non-histone proteins such as transcript factors, leading to relatively complex gene expression alterations.

Next, we determined what transcription factors are affected by JMJD1B-mediated demethylation of H4R3me2s or H3K9me2, leading to downregulation or upregulation of the genes in JMJD1B^−/−^ HSPCs. Ingenuity Pathway Analysis (IPA) causal analysis on these overlapped genes from the intersection of the RNA-seq and ChIP-seq datasets predicted that an increase in H4R3me2s occupancy due to JMJD1B knockout specifically affected the target genes of CTNNB1, ERG, SOX2, FOS, GATA3, and PML signaling pathways for hematopoiesis (Ito et al., 2012; Ku et al., 2012; Liebermann et al., 1998; Ng et al., 2011; Sarkar and Hochedlinger, 2013; Scheller et al., 2006) (Figure 5E), while the increase in H3K9me2 by JMJD1B knockout was predicted to affect expression of target genes of CDKN2a, STAT3, SMAD3, WT1, and FOXO1 for regulating proper hematopoietic processes (Chung et al., 2006; Cunningham et al., 2013; Larsson and Karlsson, 2005; Liu et al., 2009; Tothova et al., 2007) (Figure 5E). Both H4R3me2s and H3K9me2 affected NOTCH1 and SOX4, which are critical for hematopoiesis (Sarkar and Hochedlinger, 2013; Stier et al., 2002).

### JMJD1B and PRMT5 Work Cooperatively to Regulate the Dynamics of H4R3me2s and Hematopoietic Gene Expression

**Figure 6:**
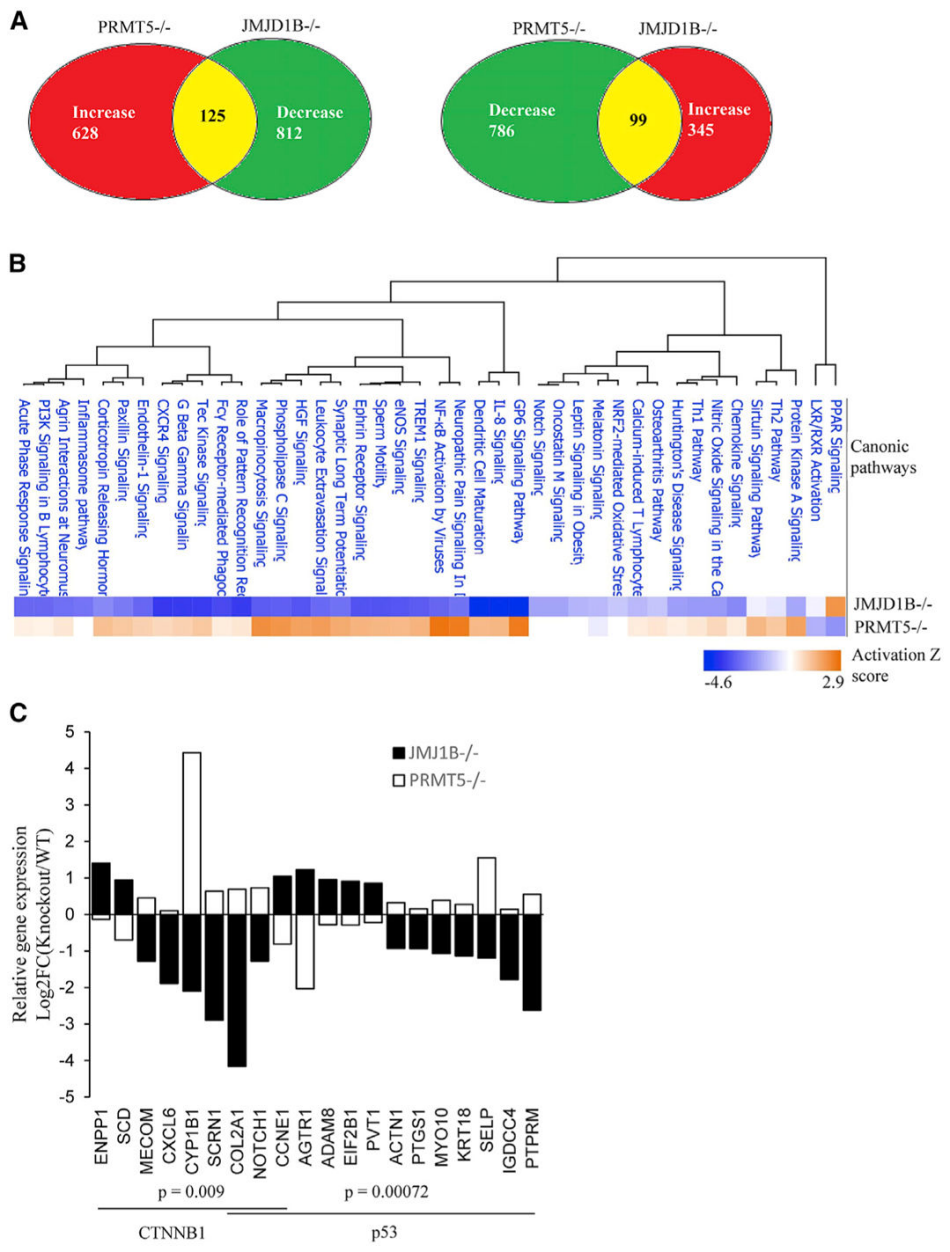
JMJD1B and PRMT5 Oppositely Regulate Genes Important for Hematopoietic Development (A) Number of genes with statistically significant changes (p < 0.05) in JMJD1B or PRMT5 knockout HSPCs, compared with corresponding WT control. The PRMT5 RNA-seq dataset was download from the GEO database (GEO: GSE69937) (Liu et al., 2015a). (B) IPA to identify the canonic pathways that are altered in JMJD1B and PRMT5 knockout cells. The canonic pathway with p < 0.01 and a calculated *Z* score are shown. (C) Top predicted transcription factor signaling and corresponding affected genes that were of relatively high H4R3me2s density (log_2_FC [JMJD1B knockout ChIP/WT ChIP] ≥ 1.5) in JMJD1B^−/−^ HSPCs and overlapped with genes whose expression was oppositely regulated by JMJD1B and PRMT5.

Given that JMJD1B is an arginine demethylase and PRMT5 is the only identified methyltransferase for SDMA on H4R3, it is plausible to hypothesize that they work in opposing fashion to regulate the dynamics of H4R3me2s and resultant gene expression. Thus, we expected that their knockout would have opposite effects on expression of at least certain genes. Supporting this hypothesis, expression of 125 genes increased (p < 0.05) in PRMT5^−/−^ HSPCs but decreased (p < 0.05) in JMJD1B^−/−^ HSPCs (Figure 6A). On the other hand, expression of 99 genes decreased (p < 0.05) in PRMT5^−/−^ HSPCs but increased (p < 0.05) in JMJD1B^−/−^ HSPCs (Figure 6A). Altogether, knockout of PRMT5 or JMJD1B altered gene expression of 224 genes in opposite direction, in contrast to 15 genes in the same direction. We conducted pathway analysis on the 1,631 genes with altered gene expression in PRMT5^−/−^ HSPCs and on 1,385 genes with altered gene expression in JMJD1B^−/−^ HSPCs. We found that JMJD1B^−/−^ HSPCs showed inhibition in 39 canonical pathways, 34 of which were activated in PRMT5^−/−^ HSPCs (Figure 6B). At the same time, JMJD1B knockout resulted in inhibition of only one canonical pathway (PPAR), while PRMT5 knockout led to its activation (Figure 6B). This finding clearly supports our assertion that JMJD1B and PRMT5 function in an opposite way in HSPCs. Finally, we showed the predicted transcription factor signaling (p < 0.05) and gene expression levels of corresponding affected genes, whose H4R3me2s density was considerably increased in JMJD1B^−/−^ versus WT HSPCs (Figure 6C). The most outstanding deduced transcription factors included p53 and CTNNB1, which are closely relevant to HSPC development.

### JMJD1B Knockout Causes Defective Hematopoiesis

**Figure 7:**
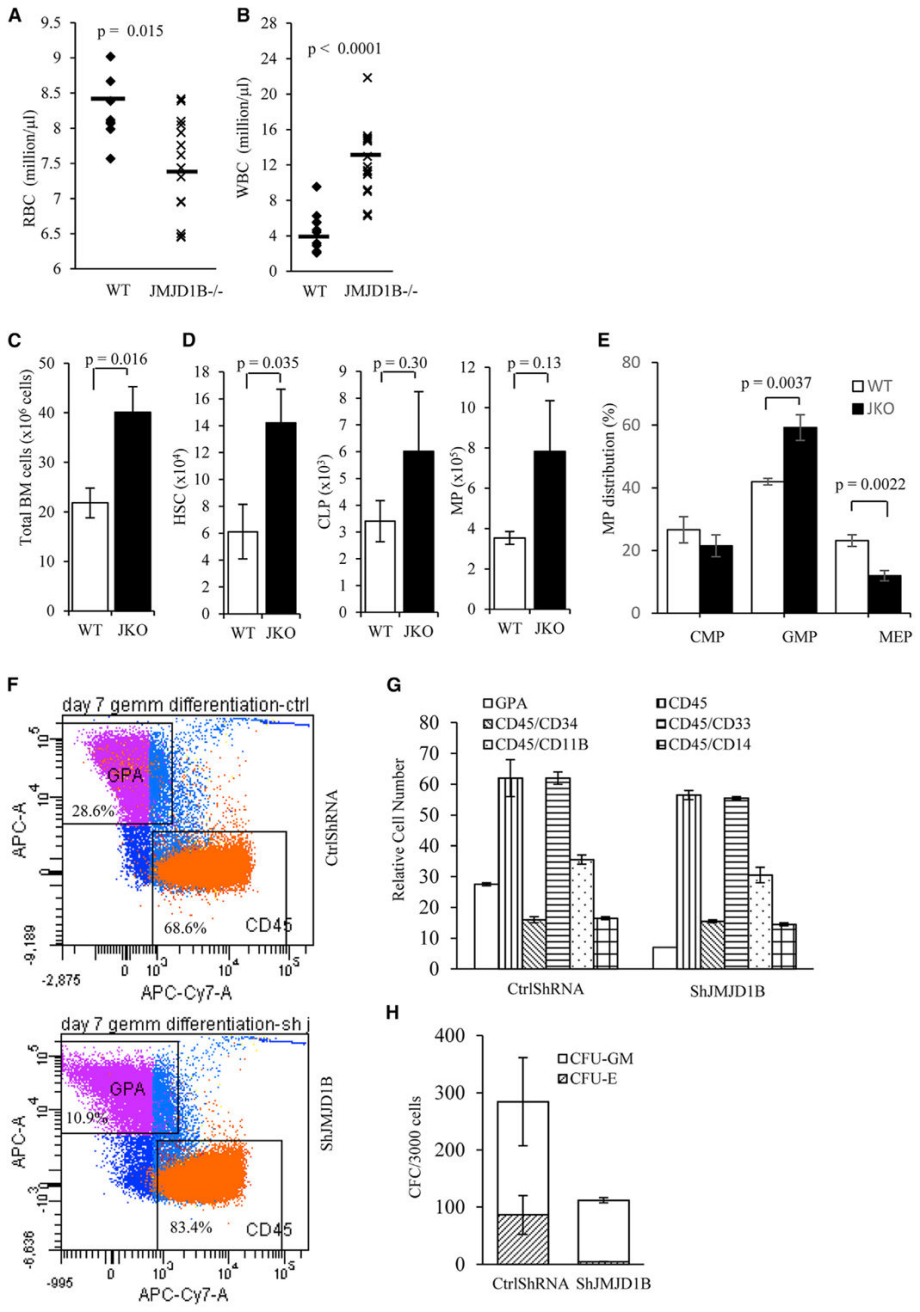
JMJD1B Deficiency Results in Abnormal Hematopoietic Development (A–E) Abnormal hematopoiesis in JMJD1B knockout mice. (A and B) Red blood cell (RBC) and white blood cell (WBC) count analysis of peripheral blood of WT (n = 10) and JMJD1B^−/−^ (n = 14) mice. (C) Total bone marrow (BM) cells from WT and JMJD1B^−/−^ (JMJD1B knockout) mice. (D) Total number of HSC (hematopoietic stem cell), CLP (common lymphoid progenitor), and MP (myeloid progenitor) cells in the BM of WT and JMJD1B^−/−^ mice. They were calculated by multiplying the total bone marrow cell number in WT and JMJ1B^−/−^ by the percentage of HSC, CLP, and MP (Figure S6A). (E) Percentage distribution of different types of MPs: CMP (common myeloid progenitor), GMP (granulocyte-monocyte progenitor), and MEP (megakaryocyte-erythrocyte progenitor) cells relative to the MP population were analyzed by flow cytometry (Figure S6B). In (C)–(E), values are mean ± SEM of five replicates. All p values were calculated using Student’s t test (two-tailed). (F–H). Knockdown of JMJD1B affects differentiation of human hematopoietic stem/progenitor cells (HSPCs). Cord blood CD34^+^ cells transduced with JMJD1B shRNA and control vector (n = 2) were selected by flow cytometry and cultured in GEMM for 7 days. Then the percentages of GPA-, CD45-, CD34-, CD11b-, CD33-, and CD14-positive cells were analyzed using flow cytometry. Representative results of flow cytometry analysis of GPA and CD45 expression are shown in (F). The total numbers of cells that expressed these markers at day 7 (G) are shown. The number of CFU-GM and BFU-E/CFU-E colonies generated from selected CD34^+^ GFP^+^ cells after culture in methylcellulose are shown in (H). See also Figures S3, S6, and S7.

To test if defects in these pathways cause defective hematopoietic phenotypes in JMJD1B^−/−^ mice, we first conducted a complete blood cell count (CBC) analysis of peripheral blood. JMJD1B^−/−^ mice (~50%) developed anemia and showed a statistically significant decrease (p = 0.015) in total red blood cells compared with WT littermates (Figure 7A). The hemoglobin of JMJD1B^−/−^ mice was 12.3 ± 0.2 g/dL, compared with 13.2 ± 0.2 g/dL in WT mice (p = 0.013). On the other hand, JMJD1B^−/−^ mice (~80%) showed a 2.7-fold increase in white blood cells in CBC analysis (p < 0.0001; Figure 7B). The cell numbers of neutrophils and lymphocytes were 4.00 ± 0.47 and 7.80 ± 0.84 k/μL, respectively, in JMJD1B^−/−^ mice, compared with 0.92 ± 0.15 and 3.44 ± 0.58 k/μL in WT mice (p < 0.001 for both cases). JMJD1B^−/−^ mice also displayed a distinct blood cell lineage distribution, with an increase of neutrophils, from the WT (Figure S5A). These results were further confirmed by flow cytometry analysis (Figures S5B and S5C). In addition, JMJD1B^−/−^ mice showed a decrease in the percentage of B lymphocytes and an increase in the T lymphocytes (Figures S5D and S5E).

We next conducted analysis of steady-state hematopoiesis on the bone marrow to identify the origin of the biased lineage. Consistent with downregulation of genes for cell-cycle arrest and cell death, the total numbers of BMCs and HSCs significantly increased in JMJD1B^−/−^ mice compared with the WT (Figures 7C and 7D). The percentage of HSC but not common lymphoid progenitor (CLP) or myeloid progenitor (MP) population in JMJD1B^−/−^ bone marrow increased, compared with the WT (Figure S6A). Although the number or percentage of MP cells had no statistically significant differences (Figures 7D and S6A), the distribution of MP in JMJD1B^−/−^ bone marrow was distinct from that of the WT (Figure 7E). The megakaryocyte-erythrocyte progenitor (MEP) population was significantly reduced in JMJD1B^−/−^ mice compared with WT littermates (p = 0.0022; Figure 7E). Also, the granulocyte-monocyte progenitor (GMP) population in JMJD1B^−/−^ mice was significantly increased (p = 0.0037; Figures 7E and S6B). MEP and GMP cells differentiate into erythrocytes and neutrophils, respectively. Consistent with this, we observed relatively higher granulocyte in JMJD1B^−/−^ bone marrow than in the WT (Figure S6C). These findings were consistent with the observation of less red blood cells and more white blood cells in JMJD1B^−/−^ peripheral blood than in WT (Figures 7A and 7B). We further determined if JMJD1B similarly regulates human CD34^+^ HSPC differentiation. Knockdown of JMJD1B expression in human HSPCs reduced the proportion of glycophorin A (GPA)-expressing erythroid cells and thus increased the proportion of CD45^+^ leukocytes and CD33^+^ myeloid cells (Figures 7F and 7G). Inhibition of JMJD1B also completely constrained BFU-E/CFU-E (erythroid colony) formation from HSPC cells (Figure 7H). These results suggest that JMJD1B regulates the growth or differentiation of human primitive hematopoietic cells.

## DISCUSSION

Our present study reveals that arginine demethylation of H4R3me2s is an active cellular process in HSPCs during hematopoiesis. The abundance of H4R3m2s as well as H3K9me2 markers inversely correlates with gene expression in HSPCs. This is consistent with previous findings, which implied that both H4R3me2s and H3K9me2 markers are repressive epigenetic markers for gene expression, possibly via modulation of the DNA methylation status and recruitment of transcription regulators (Chen et al., 2012; Girardot et al., 2014; Kim et al., 2012; Schones et al., 2014; Zhao et al., 2009). The evidence from our present study demonstrates the existence of arginine demethylases *in vivo* and a role for JMJD1B in demethylation of H4R3me2s to induce gene expression for hematopoiesis. Deficiency in JMJD1B disrupts the dynamics of H4R3me2s and H3K9me2 at the promoter regions of distinct groups of the genes that are important for development and differentiation of HSPCs, altering their expression. Consequently, JMJD1B^−/−^ mice displayed abnormal phenotypes in the hematopoietic system, which are correlated with myelodysplastic syndrome (MDS), including leukocytosis, mild anemia, and granulocytosis. The JMJD1B gene is located at a locus (chromosome 5q arm) commonly deleted in a subcategory of MDS patients (5q syndrome) (Beurlet et al., 2013). Our results suggest that JMJD1B might be a tumor suppressor gene and contribute to MDS through regulation of the H4R3me2s marker to regulate gene expression. One likely mechanism is that JMJD1B is required for maintenance of the H4R3me2s demethylation status for the induction of p53-mediated cell cycle checkpoint and cell death genes. This induction would allow HSPC cell cycle progression and proliferation to continue in a properly controlled manner. Supporting this hypothesis, we show that p53 signaling and corresponding affected genes that were of high H4R3me2s density in JMJD1B^−/−^ HSPCs and were overlapped with genes whose expression was oppositely regulated by JMJD1B and PRMT5. On the other hand, we consider that JMJD1B-mediated H4R3me2s demethylation is only one of the mechanisms, by which JMJD1B regulates hematopoietic gene expression. We observed that many genes, which are regulated by both JMJD1B and PRMT5, displayed little change in H4R3me2s density in the WT and JMJD1B^−/−^ HSPCs. It suggests that JMJD1B and PRMT5 may also cooperatively regulate the dynamics of arginine methylation status of non-histone proteins such as transcription regulators to control gene expression for hematopoiesis and MDS avoidance.

We have demonstrated that JMJD1B demethylates both H3K9me2 and H4R3me2s substrates with similar efficiency *in vitro*. However, JMJD1B distinctly mediates H3K9me2 and H4R3me2s demethylation at different loci *in vivo*. It may simultaneously demethylate H3K9me2 and H4R3me2s in groups of genes, including the target genes of NF-kB. It may primarily demethylate H3K9me2 but not H4R3me2s at certain groups of genes, including the target genes of STAT3, SMAD3, and WT1, and it may only demethylate H4R3me2 but not H3K9me2s in the groups of genes such as those regulated by transcription factors CTNNB1, ERG, and SOX2. We hypothesize that during hematopoiesis and other development processes, other factors including nearby histone modification status, JMJD1B modifications, proteins bound to chromosome and/or JMJD1B may determine whether JMJD1B demethylate H3K9me2, H4R3me2s, or both.

An interesting question is whether inhibition of PRMT5 may rescue the phenotype because of JMJD1B deletion. PRMT5 and JMJD1B possess the reversal enzyme activities, and we revealed that knockout of PRMT5 resulted in opposed molecular changes to JMJD1B knockout. For instance, PRMT5 deletion resulted in p21 (Cdkn 1a) upregulation in HSPCs (Liu et al., 2015a), but JMJD1B knockout led to p21 downregulation in HSPCs. Consequently, PRMT5 knockout mice had a decrease in the number of HSPCs and white blood cells (Liu et al., 2015a), but JMJD1B^−/−^ knockout mice had an increase in the number of HSPCs and white blood cells. Likewise, JMJD1B deficiency may promote cancer cell proliferation and survival via inhibition of p53-p21 signaling. Such alterations might be corrected by inhibition of PRMT5 to induce p21 expression. However, inhibition of PRMT5 may not completely rescue the phenotypes of the JMJD1B^−/−^ mice because the arginine demethylation is not simple reversal of methylation in cells. We suggest that JMJD1B works with PRMT5 to program the dynamic histone arginine methylation balance for turning hematopoietic development genes on/off in a sequential manner. Deficiency in either PRMT5 or JMJD1B would disrupt such an epigenetic program and lead to abnormal development of germ cells (Liu et al., 2015b, 2015c).

JmjC domain-containing proteins are the largest class of potential histone demethylases, and the JmjC domains of several proteins have been shown to demethylate mono-, di-, and tri-methyl lysine residues via an oxidative reaction that requires iron and α-ketoglutarate (Cloos et al., 2008; Klose et al., 2006; Klose and Zhang, 2007). However, the enzyme activities and specificities of most JmjC domain-containing proteins have not been experimentally tested (Greer and Shi, 2012), and it is possible that additional JmjC domain-containing arginine demethylases exist and are important in demethylating methylarginine in different histones or non-histone proteins to regulate different cellular processes. Indeed, JMJD1B knockout only increased the H4R3me2s signal in a group of genes depleted of H4R3me2s. It is consistent with our suggestion that additional arginine demethylases are important in maintaining the demethylation status of H4R3me2s and other histone arginine methylation markers. Our present study describes an important function for JmjC domain-containing enzymes in regulating arginine methylation and demonstrates the need to more clearly define the biochemical properties and *in vivo* functions of other JmjC domain containing enzymes.

## EXPERIMENTAL PROCEDURES

Further details and an outline of resources used in this work can be found in Supplemental Experimental Procedures.

### Animal Studies

Twenty to 40 weeks of male and female mice were used in this study. All protocols that involved animals were approved by the Institutional Animal Care and Use Committee of City of Hope in compliance with the Public Health Service Policy of the United States and all other federal, state, and local regulations. All aspects of the animal study were adequately reported following the NIH guideline.

### Quantification and Statistical Analyses

The band intensity of western blot analysis was quantified using ImageJ software (NIH). All data are expressed as mean ± SEM, and significance (p value) was calculated using two-tailed Student’s t test for between-group differences; p values < 0.05 were considered to indicate statistical significance.

## Supplementary Material

1

2

## Highlights

- JMJD1B is an arginine and lysine demethylase for H4R3me2s and H3K9me2
- JMJD1B and PRMT5 control the H4R3me2s dynamics at promoters of hematopoietic genes
- JMJD1B deficiency alters gene expression profile and impairs hematopoietic development

## References

[R1] Bedford MT, Clarke SG (2009). Protein arginine methylation in mammals: who, what, and why. Mol Cell.

[R2] Beurlet S, Chomienne C, Padua RA (2013). Engineering mouse models with myelodysplastic syndrome human candidate genes; how relevant are they?. Haematologica.

[R3] Blanc RS, Richard S (2017). Arginine methylation: the coming of age. Mol Cell.

[R4] Chang B, Chen Y, Zhao Y, Bruick RK (2007). JMJD6 is a histone arginine demethylase. Science.

[R5] Chen C, Nott TJ, Jin J, Pawson T (2011). Deciphering arginine methylation: Tudor tells the tale. Nat Rev Mol Cell Biol.

[R6] Chen X, Skutt-Kakaria K, Davison J, Ou YL, Choi E, Malik P, Loeb K, Wood B, Georges G, Torok-Storb B, Paddison PJ (2012). G9a/GLP-dependent histone H3K9me2 patterning during human hematopoietic stem cell lineage commitment. Genes Dev.

[R7] Chung YJ, Park BB, Kang YJ, Kim TM, Eaves CJ, Oh IH (2006). Unique effects of Stat3 on the early phase of hematopoietic stem cell regeneration. Blood.

[R8] Cloos PA, Christensen J, Agger K, Helin K (2008). Erasing the methyl mark: histone demethylases at the center of cellular differentiation and disease. Genes Dev.

[R9] Cunningham TJ, Palumbo I, Grosso M, Slater N, Miles CG (2013). WT1 regulates murine hematopoiesis via maintenance of VEGF isoform ratio. Blood.

[R10] Fuhrmann J, Clancy KW, Thompson PR (2015). Chemical biology of protein arginine modifications in epigenetic regulation. Chem Rev.

[R11] Girardot M, Hirasawa R, Kacem S, Fritsch L, Pontis J, Kota SK, Filipponi D, Fabbrizio E, Sardet C, Lohmann F (2014). PRMT5-mediated histone H4 arginine-3 symmetrical dimethylation marks chromatin at G + C-rich regions of the mouse genome. Nucleic Acids Res.

[R12] Greer EL, Shi Y (2012). Histone methylation: a dynamic mark in health, disease and inheritance. Nat Rev Genet.

[R13] Guo Z, Zheng L, Xu H, Dai H, Zhou M, Pascua MR, Chen QM, Shen B (2010). Methylation of FEN1 suppresses nearby phosphorylation and facilitates PCNA binding. Nat Chem Biol.

[R14] Huang H, Sabari BR, Garcia BA, Allis CD, Zhao Y (2014). SnapShot: histone modifications. Cell.

[R15] Ito K, Carracedo A, Weiss D, Arai F, Ala U, Avigan DE, Schafer ZT, Evans RM, Suda T, Lee CH, Pandolfi PP (2012). A PML–PPAR-δ pathway for fatty acid oxidation regulates hematopoietic stem cell maintenance. Nat Med.

[R16] Jansson M, Durant ST, Cho EC, Sheahan S, Edelmann M, Kessler B, La Thangue NB (2008). Arginine methylation regulates the p53 response. Nat Cell Biol.

[R17] Kim JY, Kim KB, Eom GH, Choe N, Kee HJ, Son HJ, Oh ST, Kim DW, Pak JH, Baek HJ (2012). KDM3B is the H3K9 demethylase involved in transcriptional activation of lmo2 in leukemia. Mol Cell Biol.

[R18] Klose RJ, Zhang Y (2007). Regulation of histone methylation by demethylimination and demethylation. Nat Rev Mol Cell Biol.

[R19] Klose RJ, Kallin EM, Zhang Y (2006). JmjC-domain-containing proteins and histone demethylation. Nat Rev Genet.

[R20] Ku CJ, Hosoya T, Maillard I, Engel JD (2012). GATA-3 regulates hematopoietic stem cell maintenance and cell-cycle entry. Blood.

[R21] Larsson J, Karlsson S (2005). The role of Smad signaling in hematopoiesis. Oncogene.

[R22] Le Romancer M, Treilleux I, Leconte N, Robin-Lespinasse Y, Sentis S, Bouchekioua-Bouzaghou K, Goddard S, Gobert-Gosse S, Corbo L (2008). Regulation of estrogen rapid signaling through arginine methylation by PRMT1. Mol Cell.

[R23] Lee KH, Ju UI, Song JY, Chun YS (2014). The histone demethylase PHF2 promotes fat cell differentiation as an epigenetic activator of both C/EBPα and C/EBPδ. Mol Cells.

[R24] Liebermann DA, Gregory B, Hoffman B (1998). AP-1 (Fos/Jun) transcription factors in hematopoietic differentiation and apoptosis. Int J Oncol.

[R25] Liu Y, Elf SE, Miyata Y, Sashida G, Liu Y, Huang G, Di Giandomenico S, Lee JM, Deblasio A, Menendez S (2009). p53 regulates hematopoietic stem cell quiescence. Cell Stem Cell.

[R26] Liu F, Cheng G, Hamard PJ, Greenblatt S, Wang L, Man N, Perna F, Xu H, Tadi M, Luciani L, Nimer SD (2015a). Arginine methyltransferase PRMT5 is essential for sustaining normal adult hematopoiesis. J Clin Invest.

[R27] Liu Z, Chen X, Zhou S, Liao L, Jiang R, Xu J (2015b). The histone H3K9 demethylase Kdm3b is required for somatic growth and female reproductive function. Int J Biol Sci.

[R28] Liu Z, Oyola MG, Zhou S, Chen X, Liao L, Tien JC, Mani SK, Xu J (2015c). Knockout of the histone demethylase Kdm3b decreases spermatogenesis and impairs male sexual behaviors. Int J Biol Sci.

[R29] Mantri M, Krojer T, Bagg EA, Webby CJ, Butler DS, Kochan G, Kavanagh KL, Oppermann U, McDonough MA, Schofield CJ (2010). Crystal structure of the 2-oxoglutarate- and Fe(II)-dependent lysyl hydroxylase JMJD6. J Mol Biol.

[R30] Mikhaleva II, Prudchenko IA, Ivanov VT, Voitenkov VB (2011). JmjC-domain-containing histone demethylases of the JMJD1B type as putative precursors of endogenous DSIP. Peptides.

[R31] Ng AP, Loughran SJ, Metcalf D, Hyland CD, de Graaf CA, Hu Y, Smyth GK, Hilton DJ, Kile BT, Alexander WS (2011). Erg is required for self-renewal of hematopoietic stem cells during stress hematopoiesis in mice. Blood.

[R32] Qi HH, Sarkissian M, Hu GQ, Wang Z, Bhattacharjee A, Gordon DB, Gonzales M, Lan F, Ongusaha PP, Huarte M (2010). Histone H4K20/H3K9 demethylase PHF8 regulates zebrafish brain and craniofacial development. Nature.

[R33] Sarkar A, Hochedlinger K (2013). The sox family of transcription factors: versatile regulators of stem and progenitor cell fate. Cell Stem Cell.

[R34] Scheller M, Huelsken J, Rosenbauer F, Taketo MM, Birchmeier W, Tenen DG, Leutz A (2006). Hematopoietic stem cell and multilineage defects generated by constitutive beta-catenin activation. Nat Immunol.

[R35] Schones DE, Chen X, Trac C, Setten R, Paddison PJ (2014). G9a/GLP-dependent H3K9me2 patterning alters chromatin structure at CpG islands in hematopoietic progenitors. Epigenetics Chromatin.

[R36] Shi Y, Lan F, Matson C, Mulligan P, Whetstine JR, Cole PA, Casero RA, Shi Y (2004). Histone demethylation mediated by the nuclear amine oxidase homolog LSD1. Cell.

[R37] Sims RJ, Rojas LA, Beck DB, Bonasio R, Schüller R, Drury WJ, Eick D, Reinberg D (2011). The C-terminal domain of RNA polymerase II is modified by site-specific methylation. Science.

[R38] Stier S, Cheng T, Dombkowski D, Carlesso N, Scadden DT (2002). Notch1 activation increases hematopoietic stem cell self-renewal in vivo and favors lymphoid over myeloid lineage outcome. Blood.

[R39] Tothova Z, Kollipara R, Huntly BJ, Lee BH, Castrillon DH, Cullen DE, McDowell EP, Lazo-Kallanian S, Williams IR, Sears C (2007). FoxOs are critical mediators of hematopoietic stem cell resistance to physiologic oxidative stress. Cell.

[R40] Walport LJ, Hopkinson RJ, Chowdhury R, Schiller R, Ge W, Kawamura A, Schofield CJ (2016). Arginine demethylation is catalysed by a subset of JmjC histone lysine demethylases. Nat Commun.

[R41] Webby CJ, Wolf A, Gromak N, Dreger M, Kramer H, Kessler B, Nielsen ML, Schmitz C, Butler DS, Yates JR (2009). Jmjd6 catalyses lysyl-hydroxylation of U2AF65, a protein associated with RNA splicing. Science.

[R42] Yang Y, Bedford MT (2013). Protein arginine methyltransferases and cancer. Nat Rev Cancer.

[R43] Yu Z, Chen T, Hébert J, Li E, Richard S (2009). A mouse PRMT1 null allele defines an essential role for arginine methylation in genome maintenance and cell proliferation. Mol Cell Biol.

[R44] Zhao Q, Rank G, Tan YT, Li H, Moritz RL, Simpson RJ, Cerruti L, Curtis DJ, Patel DJ, Allis CD (2009). PRMT5-mediated methylation of histone H4R3 recruits DNMT3A, coupling histone and DNA methylation in gene silencing. Nat Struct Mol Biol.

