# Supplementary material for: JMJD1B Demethylates H4R3me2s and H3K9me2 to Facilitate Gene Expression for Development of Hematopoietic Stem and Progenitor Cells
Source: Cell Rep. Author manuscript; Available in PMC 2018 May 3. (PMC5933860; doi:10.1016/j.celrep.2018.03.051)
Supplement: 1 [file NIHMS961949-supplement-1.pdf]

Cell Reports, Volume *23*

Supplemental Information

JMJD1B Demethylates H4R3me2s and H3K9me2 to Facilitate Gene Expression for Development of Hematopoietic Stem and Progenitor Cells

Sihui Li, Shafat Ali, Xiaotao Duan, Songbai Liu, Juan Du, Changwei Liu, Huifang Dai, Mian Zhou, Lina Zhou, Lu Yang, Peiguo Chu, Ling Li, Ravi Bhatia, Dustin E. Schones, Xiwei Wu, Hong Xu, Yuejin Hua, Zhigang Guo, Yanzhong Yang, Li Zheng, and Binghui Shen

Supplemental Table S1: Methylated histone tail peptide substrates* and antibodies used in western blotting analysis. Related to Figure 1 and Figure 3

Substrates	Sequences	Peptide Resources	Antibody Resources
H3K9me2	ARTKQTAR K(Me2) - STGGKAPRKQLAGGK(Biotin)	AnaSpec 64627-025	Abcam Ab1220
H3K4me2	ARTK(Me2)QTARKSTGGKAPRKQLA-GGK(Biotin)	AnaSpec 64356-025	Active Motif 39679
H3R2me2s	AR(Me2s)TKQTARKSTGGKAPRKQLA-GGK(Biotin)	AnaSpec 64631-025	Active Motif 39703
H3R2me2a	AR(Me2a)TKQTARKSTGGKAPRKQLA-GGK(Biotin)	AnaSpec 64630-025	Abcam Ab80075
H3R2me1	AR(Me1)TKQTARKSTGGKAPRKQLAGG K(Biotin)	AnaSpec 64629-025	Abcam ab15584
H3R8me2s	ARTKQTAR(Me2s)KSTGGKAPRKQLA GGK(Biotin)	AnaSpec 65351-025	Said Sif Ohio State Univ.
H3R8me1	ARTKQTAR(Me1)KSTGGKAPRKQ-LAK(Biotin)	AnaSpec 64607-025	Active Motif 39673
H3R17me2a	ARTKQTARKSTGGKAPR(Me2a)-KQLAGGK(Biotin	AnaSpec 64634-025	Active Motif 39709
H3R26me2a	APRKQLATKAAR(Me2a)KSAPATGGVK-GGK(Biotin)	AnaSpec 65261-025	EMD Millipore 07-215
H4R3me2s	SGR(Me2s)GKGGKGLGKGGAKRHRKV-GGK(Biotin)	AnaSpec 65424-025	Active Motif 61187
H4R3me2a	SGR(Me2a)GKGGKGLGKGGAKRHRKV-GGK(Biotin)	AnaSpec 64976-025	Active Motif 39706
H4R3me1	SGR(Me1)GKGGKGLGKGGAKRHRKV-GGK(Biotin)	AnaSpec 64977-025	Abcam Ab17339

*Custom peptide synthesis

Supplemental Table S2: Peptides* for arginine demethylation assay by mass spectrometry. Related to Figure 2

Peptides	Sequences	Resources
H3K9me2	ARTKQTAR K(Me2) STGGKAPRKQLATKAA	Anaspec 62622-5
H4R3me2s	SGR(Me2s)GKGGKGLGKGGAK	Anaspec 62392-1
H4R3me2a	SGR(Me2a)GKGGKGLGKGGAK	Anaspec 62622-3
H3R2me1	SGR(Me1)GKGGKGLGKGGAK	Anaspec 62392-2

*Custom peptide synthesis

Supplemental Table S3: Summary of ChIP-Seq and alignment. Related to Figure 4 and Figure 5.

Sample	Total Reads	Unique Aligned Reads	% Aligned	Non-redundant Reads
WT-HSPC-INPUT	58,792,986	49,010,628	83.4%	46,407,639
WT-HSPC-H4R3ME2S	117,604,443	92,976,262	79.1%	83,718,326
WT-HSPC-H3K9ME2	115,014,233	87,872,149	76.4%	81,224,010
WT-HSPC-H4	112,649,572	81,278,139	72.2%	71,254,082
WT-BMC-INPUT	62,493,414	51,494,573	82.4%	48,205,709
WT-BMC-H4R3ME2S	40,909,933	33,873,424	82.8%	27,724,518
WT-BMC-H3K9me2	50,164,784	38,526,554	77.0%	36,238,249
JKO-HSPC-INPUT	54,501,763	45,248,507	83.4%	42,962,961
JKO-HSPC-H4R3ME2S	102,856,353	83,608,487	81.3%	76,948,453
JKO-HSPC-H3K9ME2	101,544,449	83,127,518	81.9%	77,417,891
JKO-HSPC-H4	107,300,436	77,308,068	72.0%	67,765,370
JKO-BMC-INPUT	63,118,338	51,441,445	82.0%	48,083,779
JKO-BMC-H4R3ME2S	42,059,087	32,848,146	78.0%	30,027,216
JKO-BMC-H3K9ME2	53,731,355	43,200,009	80.0%	40,995,991

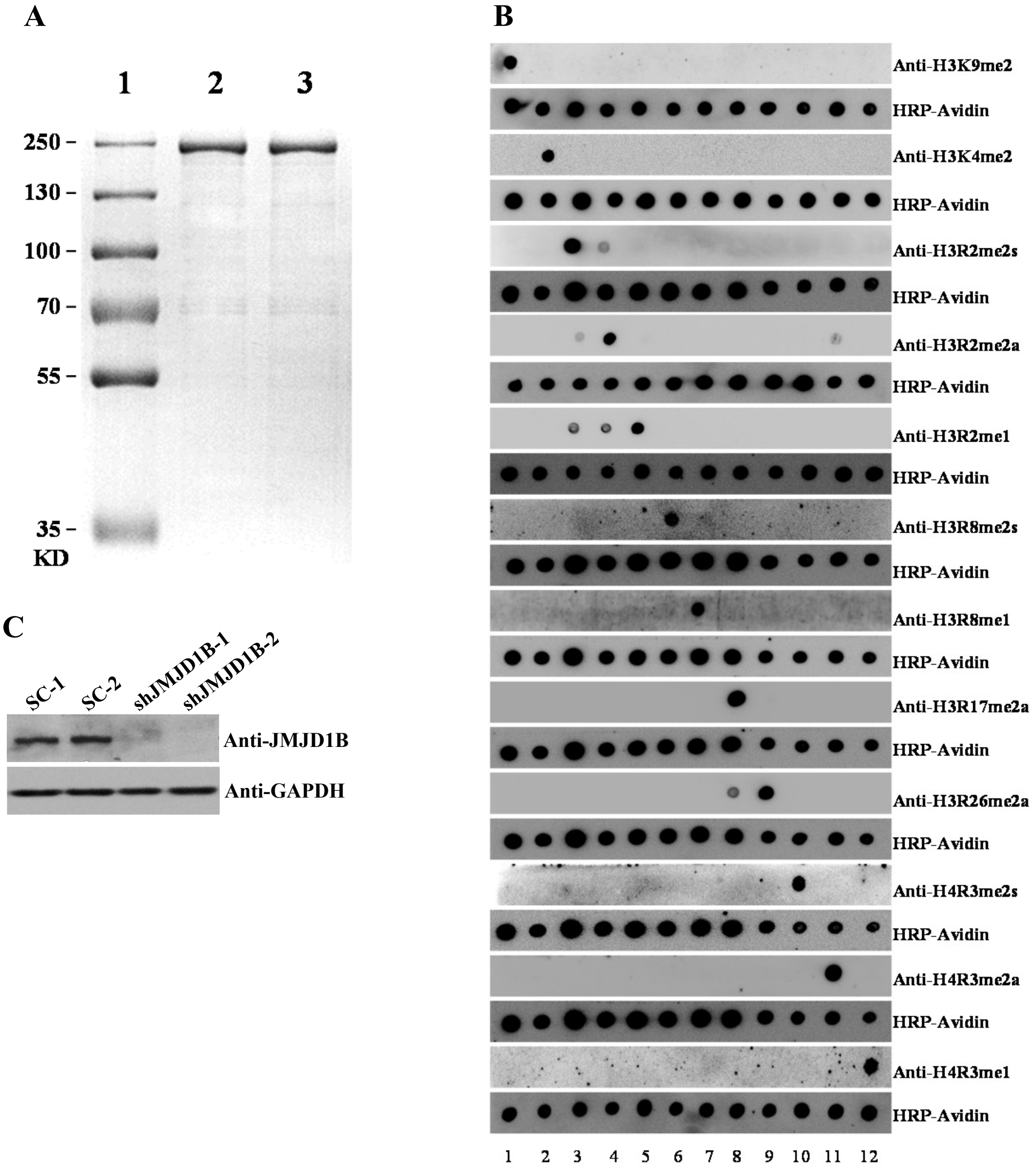

Supplemental Figure S1. Validation of purity of recombinant JMJD1B proteins, specificity of histone antibodies, and siRNA efficiency. Related to Figures 1-3. (A) JMJD1B expression and purification. PEZ-M12-JMJD1B (isoform 1) was purchased from GeneCopoeia (EX-E1505-M12) with a 3 x FLAG tag. JMJD1B protein After expression in HEK293T cells, the recombinant proteins were purified using M2 magnetic beads (Sigma). Shown is the Coomassie Brilliant Blue-stained gel demonstrating the purity of the WT (lane 2) and mutant (H1560A/D1562A/H1689A) (lane 3) JMJD1B proteins, along with molecular weight markers (Lane 1). **(B)** Dot blot analyses verify the specificity of histone antibodies. Equal amount (1 μg) of biotin-synthetic modified histone peptides were dotted on the nitrocellulose membrane at the following order from left to right: 1. H3K9me2; 2. H3K4me2; 3. H3R2me2s; 4. H3R2me2a; 5. H3R2me1; 6. H3R8me2s; 7. H3R8me1; 8. H3R17me2a; 9. H3R26me2a; 10. H4R3me2s; 11. H4R3me2a; 12. H4R3me1. Standard dot blot protocol was performed using indicated antibodies against specific forms of modified histones. For controls, membranes were stripped and reprobed with HRP-avidin. **(C)** Knockdown of JMJD1B by shRNA in 293T cells. 293T cells were transfected with scrambled shRNA vectors (SC-1: Cat No. SHC001 and SC-2: SHC002) or shRNA vectors against JMJD1B [shJMJD1B-1: Cat No. TRCN0000017093 and shJMJD1B-2: (Sigma, Cat No. TRCN0000017093). 48 h post-transfection, the JMJD1B level was analyzed with western blot using the anti-JMJD1B antibody. GAPDH was used as a loading control.

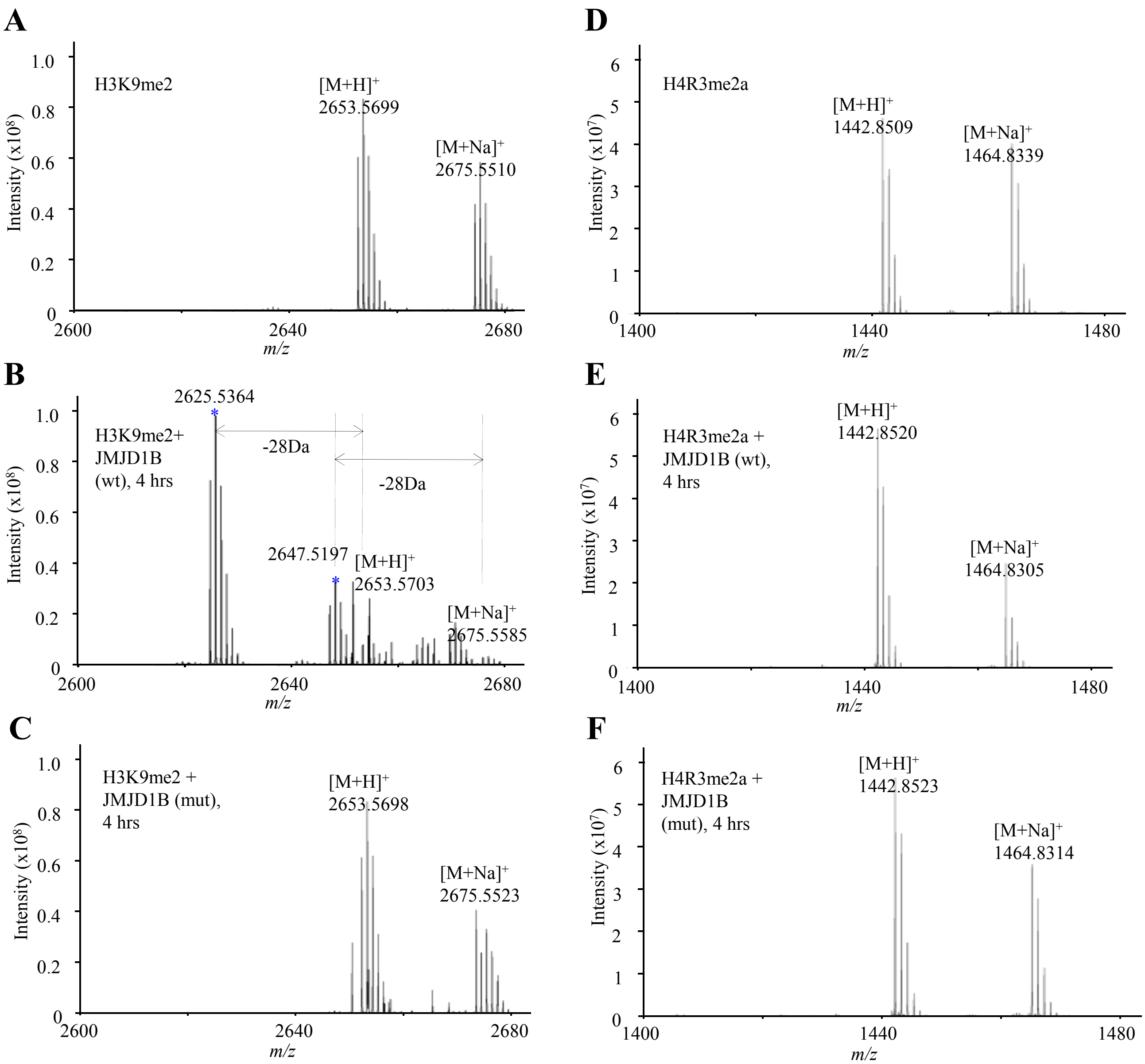

Supplemental Figure S2. JMJD1B demethylase activity assay on H3K9me2 and H4R3me2a by mass spectrometry. Related to Figure 2. (**A**)-(**C**) H3K9me2 is demethylated by JMJD1B. The H3K9me2 peptide substrate (18 µM) was incubated at 37ºC for 4 h in demethylation buffer alone (**A**), or with 0.5 µM wild-type JMJD1B (wt) (**B**) or 0.5 µM mutant JMJD1B (mut) (**C**). The reaction mixtures were analyzed by mass spectrometry. Representative mass spectrometry images are shown, and (*) indicates the demethylation products in the reactions containing the H3K9me2 peptide and WT JMJD1B (B). No product was found in the reaction with the mutant JMJD1B (**C**). (**D**)-(**F**) H4R3me2a is not demethylated by JMJD1B. The H4R3me2a peptide substrate (34 µM) was incubated at 37ºC for 4 h in demethylation buffer alone (**D**), or with 0.5 µM of JMJD1B (wt) (**E**) or 0.5 µM of JMJD1B (mut) (**F**). The reaction mixtures were analyzed by mass spectrometry. Representative mass spectrometry images are shown, demonstrating the absence of products in the reactions containing JMJD1B and the H4R3me2a peptide (**E**).

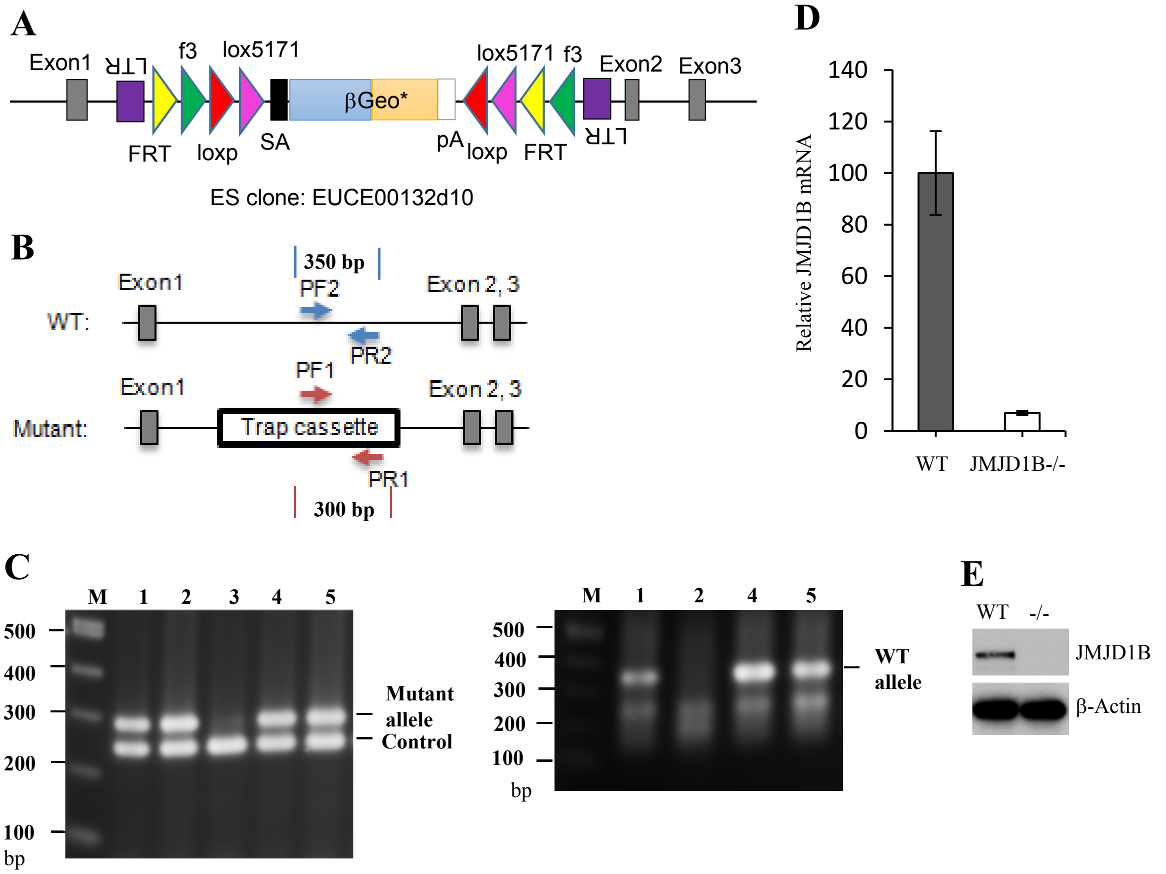

Supplemental Figure S3. Knockout of JMJD1B via gene trap technology. Related to Figures 5 and Figure 7. (**A**) Schematic map indicating insertion of the gene trap cassette (rFlipROSAbeta-Geo*) into intron 1 of JMJD1B. The gene cassette carrying a splicing acceptor (SA) at the 5' end and a polyA sequence at the 3' end causes premature termination of transcription and splicing, abolishing JMJD1B expression. (**B**) and (**C**) Screening of JMJD1B mutant mice by PCR. Panel b shows the strategy and primers for PCR amplification. The mutant allele was detected by PCR using the PF1/PR1 primers, which correspond to the DNA sequences of the trap cassette. The WT allele, at the trap cassette insertion site, was detected by PCR using the PF2/PR2 primers. Panel c shows representative images of agarose gel electrophoresis of PCR products amplified by PF1/PR1 (left) and PF2/PR2 (right). In the first PCR reaction (left), a pair of control primers corresponding to the actin gene was used as an internal control to ensure the quality of the PCR reactions. The first PCR reaction detected the mutant allele, and the second PCR reaction detected the WT allele. Mouse #3 (WT) carried no mutant allele; Mice #1, 4, and 5 (heterozygous) carried both the WT and mutant alleles; and mouse #2 (mutant) carried only the mutant allele. (**D**) mRNA levels of JMJD1B in WT and JMJD1B-/- MEF cells (E13.5). Total RNA was extracted from WT and JMJD1B-/- cells and real-time RT-PCR was used to quantify the levels of JMJD1B mRNA and control β-actin mRNA. The JMJD1B mRNA level was normalized to the corresponding β-actin mRNA level. The normalized JMJD1B mRNA level in the WT cells was arbitrarily set to 100. The values are the means ± s.d. of three independent assays. (**E**) Western blot to detect JMJD1B protein levels in WT and JMJD1B-/- embryos (E11.5). Whole cell extracts were prepared from WT and JMJD1B embryos and resolved by 4-20% SDS-PAGE. The JMJD1B protein was detected by western blotting using an anti-JMJD1B antibody (Bethyl).

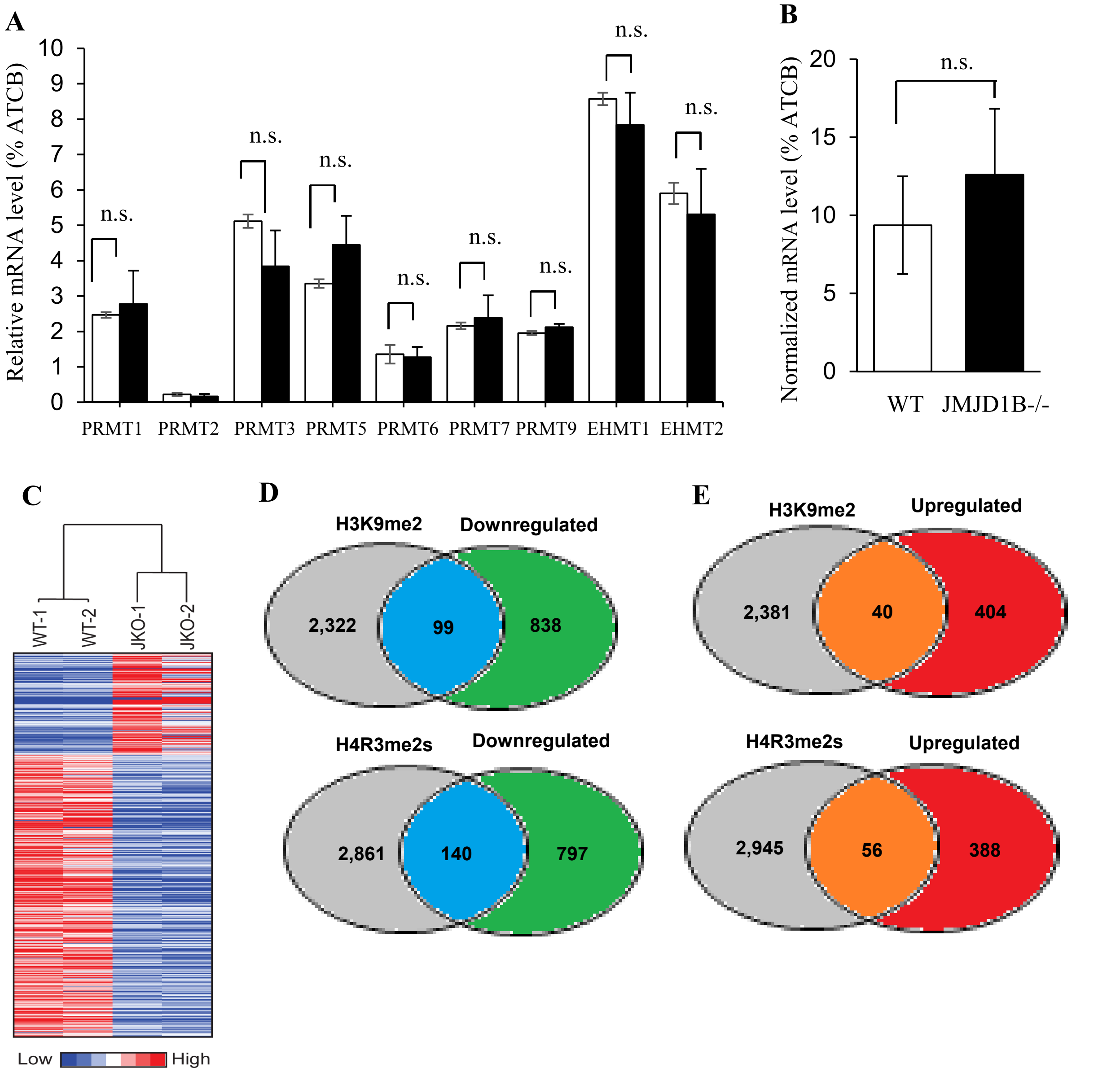

Supplemental Figure S4. Gene expression profiles in WT and JMJD1B-/- HSPCs. Related to Figure 5. (A) and (B) Relative expression of protein arginine and lysine methyltransferases in WT and JMJD1B-/- HSPCs. **(A)** The mRNA levels of PRMTs and EHMTs were determined by RNA-Seq. The relative mRNA level of PRMTs and EHMTs was normalized with corresponding mRNA level of β-actin, which is arbitrarily set as 100. Values are means ± s.d. for two biological replicates for each genetic background. (**B)** The mRNA levels of PRMT5 were determined by quantitative PCR. The relative mRNA level of PRMTs was normalized with corresponding mRNA level of β-actin, which was arbitrarily set as 100. Values are means ± s.d. for three biological replicates for each genetic background. p value: Student's t-test. **(C)** Heatmap of gene expression profilings in WT and JMJD1B$^{-/-}$ (JKO) HSPCs. **(D) and (E)** Intersection of the gene lists with increased H3K9me2 or H4R3me2s density and the gene lists of down- (Panel D) or up-regulated genes (Panel E) (JMJD1B$^{-/-}$ vs WT). Genes with FC of JMJD1B$^{-/-}$/WT in H4R3me2s or H3K9me2 signal no less than 1.5-fold and the JKO ChIP reads in H4R3me2s or H3K9me2 $\geq$ 15 were selected as increased H3K9me2 or H4R3me2s genes. Genes with FC of JMJD1B$^{-/-}$/WT in gene expression being less than or greater than 0 and $p < 0.05$ were selected as down- and up-regulated genes.

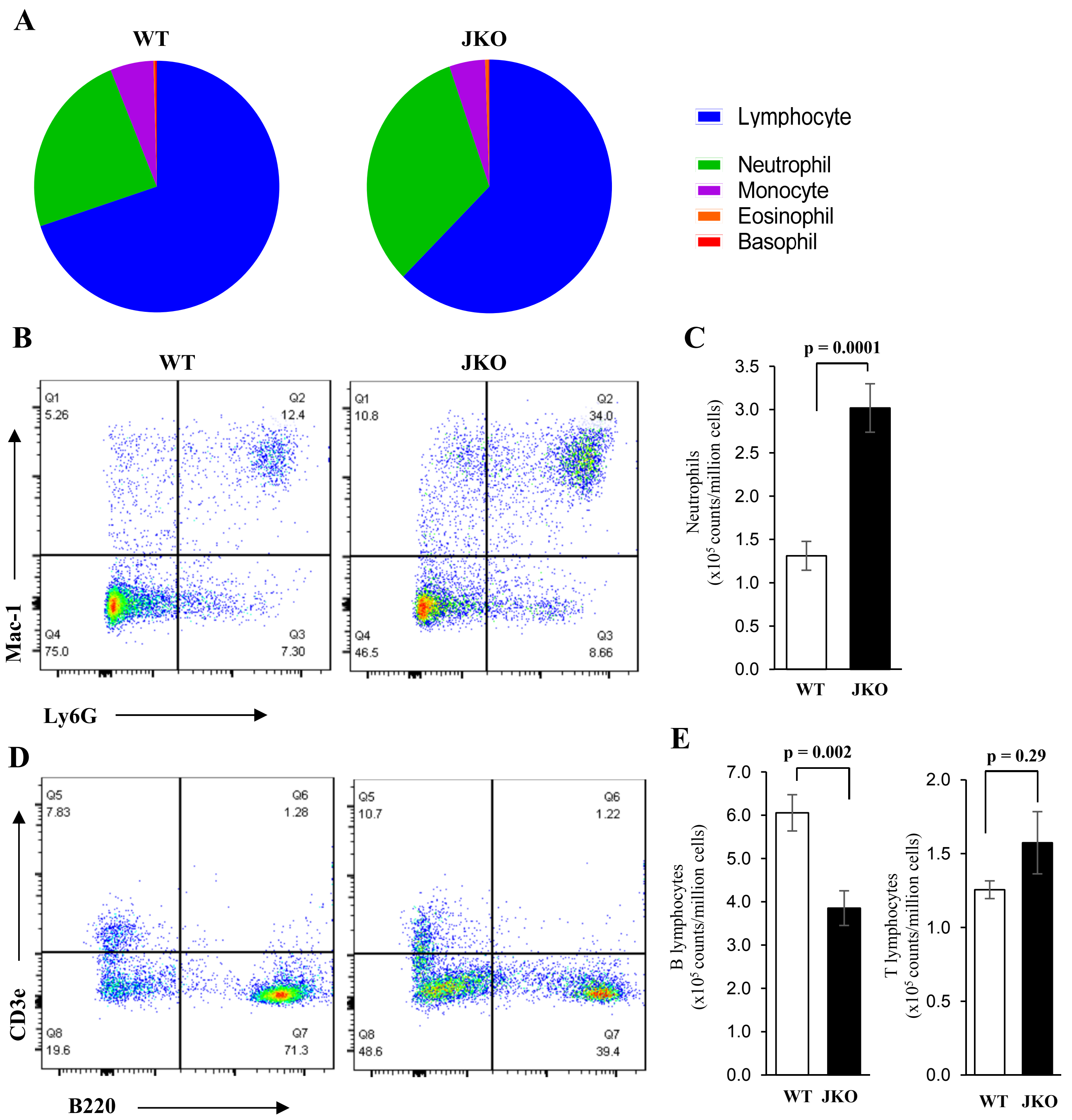

Supplemental Figure S5: Different blood cell populations in WT and JMJD1B-/- (JKO) mice. Related to Figure 7. (**A**) Complete blood count analysis and percentage distribution of lymphocytes, neutrophils, monocytes, eosinophils, and basophils in peripheral blood of wild-type (WT, n = 10) and JKO mice (n =14). Representative flow cytometry analysis of (**B, C**) granulocyte population (Gr-1^+, Mac-1^+) and total number for each genotype (mean ± s.e.m., n = 9), **(D, E)** B lymphocytes ($B220^+$) and T lymphocytes ($CD3e^+$) and total number for each genotype (mean ± s.e.m., n = 9). P values were calculated using Student's t-test.

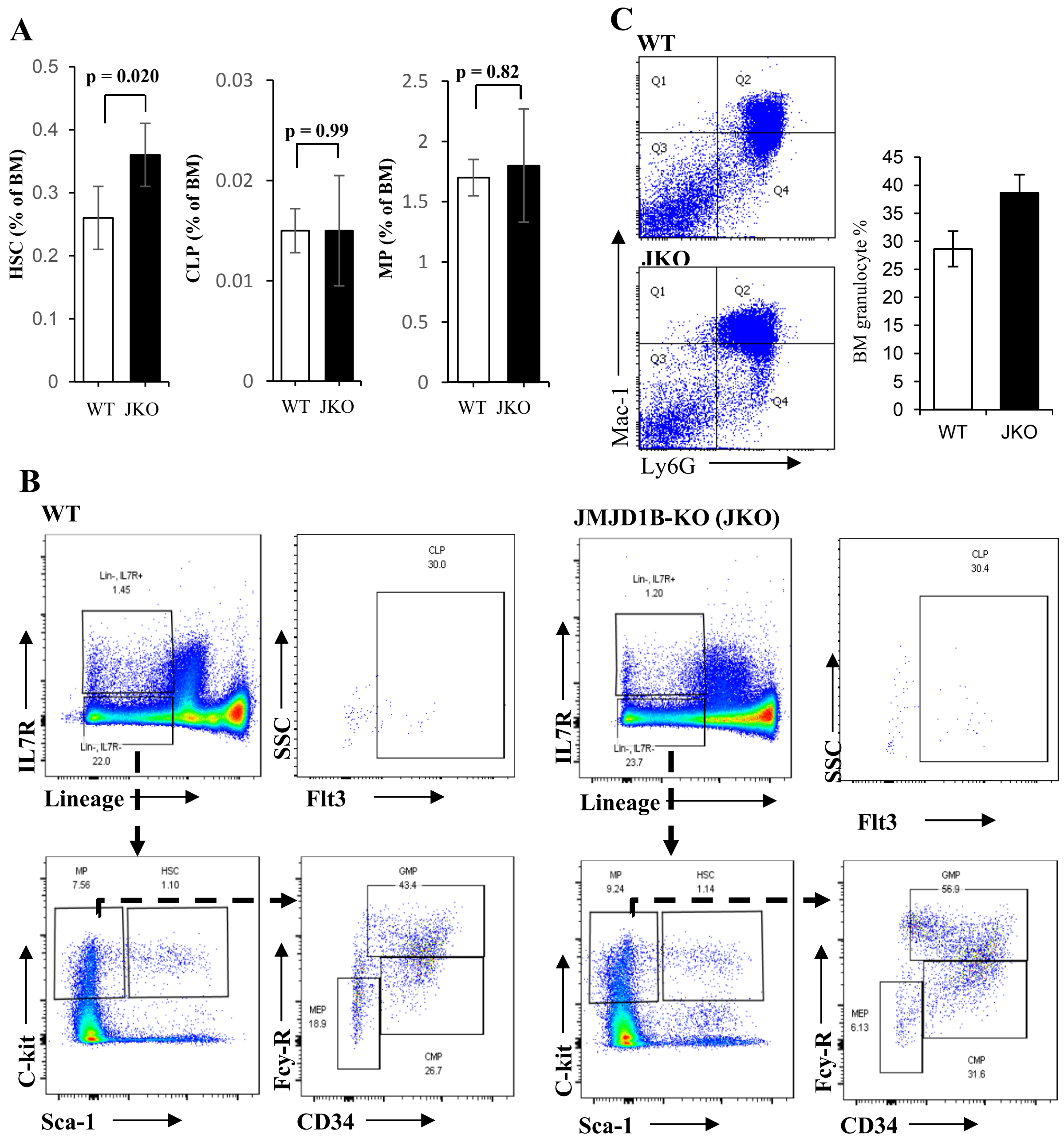

Supplemental Figure S6: Bone marrow cell population analysis by flow cytometry. Related to Figure 7. (**A**) Percentages of HSC and CLP (common lymphoid progenitor) and MP (myeloid progenitor) relative to input bone marrow cells. HSC are shown as $Lin^{-}c\text{-}Kit^{+}Sca\text{-}1^{+}$ and CLP as Lin^{-} $c\text{-}Kit^{+}Sca\text{-}1^{+}IL7Ra^{+}$ cell populations by flow cytometry. Values are means ± s.e.m of five biological replicates. P values were calculated using Student's t-test. (**B**) Representative flow cytometry analysis of bone marrow progenitor cell population compartments of age matched (22 weeks) WT (left 4 panels) and JMJD1B-/- mice (right 4 panels). Live bone marrow cells were stained for hematopoietic stem cell/progenitor cells expression marker (C-kit, Sca-1, Fcy-R, CD34, IL7R, Flt3, and CD150) along with lineage marker (Ter119, CD3, B220, NK1.1, Gr-1 and Mac-1). For CLP analysis, lineage negative and $IL7R^{+}$ were plotted for $c\text{-}kit^{+}$ and $Sca\text{-}1^{+}$ and then Flt3 positive (upper right plot). For MP analysis, lineage negative cells were divided for C-kit positive and SCA-1 negative cells (lower left plot) and Lin^{-}, $C\text{-}kit^{+}$ population was further analyze using Fcy-R and CD34 markers (lower right plot). Common myeloid progenitor (CMP) were defined as (Lin^{-}, $C\text{-}kit^{+}$, $CD34^{+}$, $Fcy\text{-}R^{mid}$), GMP as (Lin^{-}, $C\text{-}kit^{+}$, $CD34^{+}$, $Fcy\text{-}R^{hi}$) and MEP as (Lin^{-}, $C\text{-}kit^{+}$, $CD34^{-}$, $Fcy\text{-}R^{-}$). For hematopoietic stem cell (HSC) analysis, lineage negative cells were stained for C-kit and SCA-1 positive fraction (lower left plot). (**C**) Granulocyte population ($Gr\text{-}1^{+}$, $Mac\text{-}1^{+}$) in WT and JMJD1B-/- mice (means ± s.e.m of two biological replicates)

Supplemental Experimental Procedures

Cell culture

The 293T or primary MEF cells were cultured at 37°C in a 5% CO_2 standard tissue culture incubator. Dulbecco's modified Eagle's medium (DMEM, Corning Life Sciences, Corning, NY), supplemented with 10% fetal bovine serum (FBS, GE Healthcare Life Sciences, Logan, UT) and 1% penicillin-streptomycin (Thermo Fisher Scientific, Waltham, MA), was used for all routine maintenance of culture.

JMJD1B knockout in mice

JMJD1B gene expression was disrupted via gene trap technology (Supplemental Figure S3A). Male chimeric mice generated via JMJD1B mutant embryonic stem cells (*neo*$^{+}$) were crossed with female WT mice (C56BL/6 genetic background) to transmit the mutation through the germline, producing heterozygous mice on the B6.129P2 genetic background. Heterozygous (JMJD1B$^{+/-}$) and homozygous (JMJD1B$^{-/-}$) mice were selected with PCR (Supplemental Figure S3B and S3C). Homozygous knockout of JMJD1B in mice was verified by quantitative PCR to measure the mRNA levels in mouse endothelial fibroblast (MEF) cells, as well as tissue staining and western blotting to measure protein levels. No JMJD1B RNA or protein was detected *in vivo* (Supplemental Figure S3D and 3E). For phenotype characterization, we used nQuery to estimate the minimal number of mice needed for the study and Fisher exact test to calculate the p value. WT and JMJD1B$^{-/-}$ mice were randomly selected for phenotype characterization in a blind fashion. All protocols involving animals have been approved by the Institution Animal Care and

Use Committee (IACUC) of City of Hope in compliance with the Public Health Service Policy on Use of Laboratory Animals.

In vitro methylation, demethylation, and formaldehyde release assays

Recombinant WT and mutant (H1560A/D1562A/H1689A) JMJD1B were expressed as FLAG fusions using the 3xFLAG expression vector (pEZ-M12). After expression in HEK293T cells (ATCC), the proteins were purified using M2 beads, according to the manufacturer's instructions (Sigma-Aldrich). The biotin-tagged methylated histone peptide sequences and antibody resources for western blotting analysis are listed in Supplemental Tables S1 and S2. Other antibodies used in this study are also commercially available, including anti-H4 (Abcam, Cat No: ab187521), anti-JMJD1B (Cell Signaling, Cat No: 3314), HRP-labeled Streptavidin (Beyotime, Cat No: A0303), goat anti-rabbit IgG HRP (Pierce, Cat No: 31460), and goat anti-mouse IgG HRP (Pierce, Cat No: 31430). The specificity of histone antibodies was confirmed with dot blotting analysis using synthetic histone peptides (Supplemental Figure S1B).

For the demethylation reactions, the bulk histones, methylated recombinant histone H3 and H4, or synthetic peptide substrates were incubated with purified JMJD1B (0.5 μM) in demethylation buffer in a final volume of 20 μl at 37 °C. The reaction products were assayed either by western blot analysis or by using a formaldehyde release assay.

A modified version of formaldehyde release assay (Walport et al., 2016) was conducted to analyze the kinetic parameters of JMJD1B-catalyzed H4R3me2s and H3K9me2 demethylation. Briefly, JMJD1B (0.5 μM) was incubated with varying concentrations of H4R3me2s and

H3K9me2 peptide substrates in a demethylation buffer at 37°C for 0, 10, 20, 30, 40, 50, 60 minutes. After stopping the reaction with addition of EDTA to a final concentration of 10 mM, the formaldehyde concentration in each reaction was measured using a fluorescence-based formaldehyde assay kit (Sigma, St. Louis, MO) and the Km, kcat, and kcat/Km were calculated following the previously published protocol (Walport et al., 2016).

Mass spectrometry analysis

34 μM each of synthetic H4R3me2s, H4R3me2a, or H4R3me1 or 18 μM of H3K9me2 peptide substrates (Supplemental Table S1) were incubated in the absence or presence of WT (0.5 μM) or mutant (0.5 μM) JMJD1B in a demethylation buffer. After 2 or 4 h of incubation, the reaction mixtures were desalted and lyophilized. The peptide (5 μg in 100 μl demethylation buffer), was dissolved in 1 ml of 2% acetonitrile/0.1% TFA and desalted by passing through tC18 cartridges (Waters). The cartridges were activated and equilibrated using 1 ml 80% acetonitrile/0.1% TFA, followed by 1 ml 0.1% TFA (2X). The reaction mixture was loaded onto the cartridges, then washed with 1 ml 0.1% TFA (2X). The cartridges were eluted with 1 ml 80% acetonitrile/0.1% TFA and lyophilized. The lyophilized samples were then dissolved in 50% methanol/0.1% FA and analyzed by a 9.4T Q- FT-ICR MS hybrid (Solarix, Bruker Daltonics Bremen, Germany) at the National Center of Biomedical Analysis (Beijing, China).

JMJD1B overexpression and knockdown in HEK293T cells

To overexpress JMJD1B and observe its impact on arginine demethylation of H4R3me2s within the cells, PEZ-M12-JMJD1B was transfected into HEK293T cells (ATCC) using the Polyjet transfection reagent (Signagen Laboratories), according to the manufacturer's instruction. The

absence of contamination by bacteria, yeast, or other microorganisms in the cell culture was confirmed using a mycoplasma detection kit (Sigma). After 48 h, the cells were harvested. JMJD1B overexpression was confirmed by western blotting, and the levels of H3K9me2, H4R3me2s, H4R3me1, and total histone 3 or histone 4 were determined by western blotting.

To knock down JMJD1B and to observe the impact on arginine demethylation of H4R3me2s within cells, a shRNA fragment targeting the coding sequence of JMJD1B was generated using a pair of oligonucleotides, 5'CCGGCCCTAGTTCATCGCAACCTTTCTCGAG AAAGGTTGCGATGAACTAGGGTTTTTG3' and 5'AATTCAAAAACCCTAGT TCATCGCAACCTTTC TCGAGAAAGGTTGCGATGAACTAGGG3'. A scrambled shRNA fragment was produced using the oligonucleotides, 5’CCGGCCTAAGGTTAAGTCGCCCTCG CTCGAGCGAGGGCGACTTAACCTTAGGTTTTT3’ and 5’AATTCAAAAACCTAAG GTTAAGTCGCCCTCGCTCGAGCGA GGGCGACTTAACCTTAGG3’. The DNA fragments were cloned into the PLKO.1-EGFP plasmid. The HEK293T cells were co-transfected with the PLKO.1-shJMJD1B or PLKO.1-shScramble plasmid, together with the lentiviral packaging vectors pMD2.G (Addgene) and psPAX2 (Addgene), using the Polyjet transfection reagent (Signagen Laboratories). The absence of contamination by bacteria, yeast, or other microorganisms in the cell culture was confirmed using a mycoplasma detection kit (Sigma). After 48 h, the supernatants containing the released viral particles were collected and centrifuged at 2,500 x g to remove the cell debris. The viral particles were then added to nascent HEK293T cells and cultured overnight. The virus-infected HEK293T cells were replaced with fresh virus-free medium and cultured for 36 h. Individual clones of GFP-positive cells were selected by diluting the cells into 96-well plates at one cell per well.

Analysis and sorting of hematopoietic cells from bone marrow and peripheral blood

Complete blood count (CBC) analysis was conducted for whole blood using a Hemavet 950FS hematology analyzer (Drew Scientific, Miami Lake, FL). BMCs were harvested from femur, tibia and humerus and flow cytometry analysis of lineages in peripheral blood and progenitors in bone marrow were performed as described previously (Akashi et al., 2000). Briefly, the blood cells from WT and JMJD1B$^{-/-}$ mice were stained with fluorochrome conjugated antibodies (B220, Gr-1, Mac-1, CD3e, CD4). Similarly, BMCs from WT and JMJD1B$^{-/-}$ mice were stained with fluorochrome conjugated antibodies (C-kit, SCA-1, CD34, Flt3, Fcγ-Rα, IL7Ra, CD150, Biotin-lineage). The stained cells were analyzed using a BD LSRFortessa (BD Biosciences, San Jose, CA). Antibodies specific for the following surface antigens were purchased from BD Bioscience: B220 (RA3-6B2), Mac-1 (M1/70), Gr-1 (RB6-8C5), CD4 (GK1.5), CD3e (145-2C11), IL7Rα (SB/199), Sca-1 (D7), TER119(TER-119), CD34 (RAM34), Flt3 (A2F10.1), c-Kit (2B8) FcγRII/III (2.4G2), CD150 (Q38.480), Biotin-Ter119 (TER-119), Biotin-CD3e (145-2C11), Biotin Gr-1 (RB6-8C5), Biotin-Mac-1 (M1/70), Biotin-B220 (RA3-6B2) and Biotin-NK1.1 (PK136).

Hematopoietic stem cell/progenitor/mature populations were sorted based on cell surface marker. Granulocyte-monocyte progenitors were sorted as (Lin$^-$, C-kit$^+$, CD34$^+$, Fcy-R^{hi}), common myeloid progenitors as (Lin$^-$, C-kit$^+$, CD34$^+$, Fcy-R^{mid}), megakaryocyte-erythrocytes progenitors as (Lin$^-$, C-kit$^+$, CD34$^-$, Fcy-R$^-$), hematopoietic stem cell (Lin$^-$, SCA-1$^+$, C-kit$^+$), common lymphoid progenitors as (Lin$^-$, IL7Ra$^+$, c-Kit$^+$, Sca-1$^+$,Flt3$^+$). Mature granulocyte cells populations from both blood and bone marrow were sorted as Gr-1$^+$ cells. B lymphocytes and T

lymphocytes were sorted as $B220^+$ and $CD3e^+$ cell populations, respectively. If the purity of the sorted population did not reach more than 90%, the sorting was repeated.

RNA-Seq

Sequencing libraries were prepared with the TruSeq RNA Sample Prep Kit V2 (Illumina; San Diego, CA) according to the manufacturer's instruction. Briefly, poly(A) mRNA was enriched using oligo dT magnetic beads, and reverse transcribed. The resulting double-stranded cDNAs were sheared with Covaris S220 (Covaris, Spalding, UK) with the 200 bp peak, 50 μl volume setting. The fragmented cDNA underwent end repair, 3′ end adenylation. Finally, the bar-coded adapters (Illumina) were ligated to the cDNA fragments, and 10 cycles of PCR were performed to produce the final sequencing library. Library templates were prepared for sequencing using the cBot cluster generation system with the HiSeq SR Cluster Kit V4 (Illumina, San Diego, CA). The sequencing run was performed in single read mode for 51 cycles of read 1 and 7 cycles of index read using the HiSeq 2500 platform with the HiSeq SBS Kit V4 (Illumina, San Diego, CA). The real-time analysis (RTA) 2.2.38 software package was used to process the image analysis and base calling. Raw sequence reads were mapped to the mouse genome (mm9) using TopHat v2.0.8b, and the frequency of Refseq genes was counted using HTSeq-0.6.1

ChIP-Seq

ChIP on native chromatin from HSPCs and BMCs was performed following previously published protocols (Barski et al., 2007; Cuddapah et al., 2009; Girardot et al., 2014) with the following modifications using ChIP-grade Magna protein A/G beads (Cat. No: 16-663, EMB

Millipore) and ChIP-grade antibodies against H4R3me2s (Active motif 61187), H3K9me2 (Abcam, ab1220), and histone H4 (Abcam, ab10158). BMCs were isolated from age-matched WT or JMJD1B$^{-/-}$ littermates (n = 4 for each background, 20-24 weeks). Total BMCs and isolated HSPCs were pooled together and nuclei were isolated. The isolated nuclei were incubated for 15 min at 37°C with 4 units/µl of Micrococcal nuclease (M0247, New England Biolabs) to produce >90% mononucleosomes and a small portion of di-nucleosome, as verified by agarose gel electrophoresis. Nucleosomes were precipitated with control IgG or a specific histone antibody. ChIP on histone H4 was used as the reference for total nucleosomes. ChIPed or input DNA was purified with Ampure XP beads (Cat No: A63880, Beckman Coulter).

The input DNA fragments and H4R3me2s, H3K9me2, or histone H4 ChIPed DNA fragments from each sample were barcoded and sequenced on the Illumina HiSeq 2500 to produce 51 bp reads. Reads were based-called using standard Illumina software and aligned to mouse reference genome (mm9) using NovoAlign (http://www.novocraft.com/) (Supplemental Table S3). The enrichment of H4R3me2s and H3K9me2 across the genome was analyzed by MACS2 program using p value 0.001 to identify peaks as previously described (Zhang et al., 2008). The enrichment of H4R3me2s or H3K9me2 relative to histone H4 [log 2(ChIP/ChIP H4)] or relative to the input [Log2(ChIP/input)] at the promoter regions throughout the genome was calculated. Briefly, the reads in the window from 1kb upstream of the TSS to the TSS were counted and normalized with the total uniquely mapped reads. The normalized ChIP H4R3me2 or H3K9me2 was divided by the normalized H4 reads or the normalized input reads and log2FC was calculated for each gene. A pseudo read was added to each window to avoid abnormally high normalization. In both cases, RefSeq genes (mm9) was used for the gene annotation (Quinlan

and Hall, 2010). The same approach was used to calculate the Log2FC(ChIP JMJD1B/Input) and Log2FC(ChIP PRMT5/Input).

Real-time quatitiative-PCR

For mRNA level measurement, the total RNA was extracted using the RNeasy micro Kit (Qiagen, Valencia, CA). The cDNA was synthesized from 2 µg of RNA using SuperScript III RT (Thermo Fisher Scientific). Quantitative real time PCR was performed with Power SYBER Green Master Mix (Applied Biosystems) on a Bio-Rad CFX96 real time system (Bio-Rad, Hercules, CA). ATCB was used as housekeeping loading controls to normalize gene expression using the ΔΔCt method.

For ChIP-qPCR, the input genomic DNA or ChIPed DNA was used as a template. Quantitative real time PCR was performed with Power SYBER Green Master Mix (Applied Biosystems) on a Bio-Rad CFX96 real time system (Bio-Rad, Hercules, CA). The input DNA or ChIPed H4 was used as a normalization control.

Western blot

Cells were lysed in whole cell extraction buffer (50 mM Tris pH 7.5, 150 mM NaCl, 0.5% NP-40) containing protease inhibitor cocktails (Cat#: 11873580001, Sigma). The protein concentration of each sample was quantified using the Bio-Rad protein assay reagent (Cat#: 5000006, Bio-Rad). For detection of histone and histone modifications, total histones were isolated using the Histone Extraction Kit (Cat#: ab113476, Abcam). Equal amounts of whole cell extracts or total histones were resolved by 4-15% or 15% SDS-PAGE and immunoblotted with

specified antibodies. To detect the loading control including β-actin, GAPDH, histone H3, or histone H4, the blot was stripped and reprobed with an indicated antibody.

Resources Table

REAGENT or RESOURCE	SOURCE	IDENTIFIER
Antibodies		
Mouse monoclonal anti-H3K9me2	Abcam	Cat#: Ab1220<br>RRID: AB_449854
Mouse monoclonal anti-H3K4me2	Active Motif	Cat#: 39679<br>RRID:n/a
Rabbit polyclonal anti- H3R2me2s	Active Motif	Cat#: 39703<br>RRID:n/a
Rabbit polyclonal anti- H3R2me2a	Abcam	Cat#: Ab80075<br>RRID: AB_1603562
Rabbit polyclonal anti- H3R2me1	Abcam	Cat# Ab15584<br>RRID: AB_880446
Rabbit polyclonal anti- H3R8me2s	Abcam	Cat# Ab130740<br>RRID:n/a
Rabbit polyclonal anti- H3R8me1	Active Motif	Cat#: 39673<br>RRID:n/a
Rabbit polyclonal anti- H3R17me2a	Active Motif	Cat#: 39709<br>RRID:n/a
Rabbit polyclonal anti- H3R26 me2a	EMD Millipore	Cat#: 07-215<br>RRID: AB_310435
Rabbit polyclonal anti- H4R3me1	Abcam	Cat#: Ab17339<br>RRID: AB_873863
Rabbit polyclonal anti- H4R3me2s	Active Motif	Cat#:61187<br>RRID:n/a
Rabbit polyclonal anti- H4R3me2a	Active Motif	Cat#:39706<br>RRID:n/a
Rabbit polyclonal anti- H3	Abcam	Cat#:Ab1791<br>RRID: AB_302613
Rabbit polyclonal anti- H4	Abcam	Cat#:Ab10158<br>RRID: AB_296888
BUV395 Rat Anti-Mouse CD117	BD Biosciences	Cat#: 564011<br>RRID: n/a
APC-Cy™7 Rat Anti-Mouse Ly-6A/E	BD Biosciences	Cat#: 560654<br>RRID: AB_1727552
Alexa Fluor® 647 Rat anti-Mouse CD34	BD Biosciences	Cat#:560230<br>RRID: AB_1645200
PE Rat Anti-Mouse CD135	BD Biosciences	Cat#: 553842<br>RRID: AB_395079
PE-Cy™7 Rat Anti-Mouse CD16/CD32	BD Biosciences	Cat#: 560829<br>RRID: AB_10563207
BV711 Rat Anti-Mouse CD127	BD Biosciences	Cat#: 565490<br>RRID: n/a
BV421 Rat anti-Mouse CD150	BD Biosciences	Cat#: 562811<br>RRID: n/a

FITC Hamster Anti-Mouse CD48	BD Biosciences	Cat#: 557484<br>RRID: AB_396724
Biotin Rat Anti-Mouse TER-119/Erythroid Cells	BD Biosciences	Cat#: 553672<br>RRID: AB_394985
Biotin Hamster Anti-Mouse CD3e	BD Biosciences	Cat#: 553059<br>RRID: AB_394592
Biotin Rat Anti-Mouse Ly-6G and Ly-6C	BD Biosciences	Cat#: 553124<br>RRID: AB_394640
Biotin Rat Anti-Mouse CD45R/B220	BD Biosciences	Cat#: 553085<br>RRID: AB_394615
Biotin Rat Anti-CD11b	BD Biosciences	Cat#: 557395<br>RRID: AB_2296385
Biotin Mouse Anti-Mouse NK-1.1	BD Biosciences	Cat#: 553163<br>RRID: AB_394675
PE-CF594 Streptavidin	BD Biosciences	Cat#: 562284<br>RRID: AB_11154598
FITC Rat Anti-Mouse Ly-6G and LY-6C	BD Biosciences	Cat#: 553126<br>RRID: AB_394642
BV421 Rat Anti-CD11b	BD Biosciences	Cat#: 562605<br>RRID: AB_11152949
BUV395 Hamster Anti-Mouse CD3e	BD Biosciences	Cat#: 563565<br>RRID: n/a
BV711 Rat Anti-Mouse CD4	BD Biosciences	Cat#: 563050<br>RRID: n/a
PE-Cy™7 Rat Anti-Mouse CD45R/B220	BD Biosciences	Cat#: 552772<br>RRID: AB_394458
Alexa Fluor® 488 anti-mouse CD117 (c-Kit)	BioLegend	Cat#: 105815<br>RRID: AB_493473
Chemicals, Peptides, and Recombinant Proteins		
Recombinant protein human JMJD1B	Active motif	Cat#: 31429<br>RRID:
Propidium iodide	SIGMA-ALDRICH	Cat#: 11348639001<br>RRID: n/a
DAPI	SIGMA-ALDRICH	Cat#: D9542<br>RRID: n/a
Power SYBR™ Green PCR Master Mix	Thermo Fisher Scientific	Cat#: 4367659<br>RRID: n/a
Applied Biosystems™ TaqMan™ Reverse Transcription Reagents	Applied Biosystems	Cat#: N8080234<br>RRID: n/a
Critical Commercial Assays		
Direct Lineage Cell Depletion Kit, mouse	Miltenyi Biotech	Cat#: 130-110-470<br>RRID: n/a
Deposited Data		
Raw RNA-seq and ChIP-seq data	This paper	GSE94966
Experimental Models: Cell Lines		
293T cells	ATCC	CRL-3216
Experimental Models: Organisms/Strains		
JMJD1B knockout mice B6.129P2 background	This paper	n/a
Oligonucleotides		

shRNA against JMJD1B	Sigma	Cat#:<br>TRCN0000017093<br>RRID: n/a
Scramble shRNA	Sigma	Cat# SHC001<br>RRID: n/a
Recombinant DNA		
pEZ-M12-JMJD1B	This paper	n/a

Supplemental References

Akashi, K., Traver, D., Miyamoto, T., and Weissman, I.L. (2000). A clonogenic common myeloid progenitor that gives rise to all myeloid lineages. Nature *404*, 193-197.

Barski, A., Cuddapah, S., Cui, K., Roh, T.Y., Schones, D.E., Wang, Z., Wei, G., Chepelev, I., and Zhao, K. (2007). High-resolution profiling of histone methylations in the human genome. Cell *129*, 823-837.

Cuddapah, S., Barski, A., Cui, K., Schones, D.E., Wang, Z., Wei, G., and Zhao, K. (2009). Native chromatin preparation and Illumina/Solexa library construction. Cold Spring Harbor protocols *2009*, pdb prot5237.

Girardot, M., Hirasawa, R., Kacem, S., Fritsch, L., Pontis, J., Kota, S.K., Filipponi, D., Fabbrizio, E., Sardet, C., Lohmann, F., *et al.* (2014). PRMT5-mediated histone H4 arginine-3 symmetrical dimethylation marks chromatin at G + C-rich regions of the mouse genome. Nucleic acids research *42*, 235-248.

Quinlan, A.R., and Hall, I.M. (2010). BEDTools: a flexible suite of utilities for comparing genomic features. Bioinformatics *26*, 841-842.

Walport, L.J., Hopkinson, R.J., Chowdhury, R., Schiller, R., Ge, W., Kawamura, A., and Schofield, C.J. (2016). Arginine demethylation is catalysed by a subset of JmjC histone lysine demethylases. Nature communications *7*, 11974.

Zhang, Y., Liu, T., Meyer, C.A., Eeckhoute, J., Johnson, D.S., Bernstein, B.E., Nusbaum, C., Myers, R.M., Brown, M., Li, W., *et al.* (2008). Model-based analysis of ChIP-Seq (MACS). Genome biology *9*, R137.
