# Supplementary material for: JMJD1B Demethylates H4R3me2s and H3K9me2 to Facilitate Gene Expression for Development of Hematopoietic Stem and Progenitor Cells
Source: Cell Rep. Author manuscript; Available in PMC 2018 May 3. (PMC5933860; doi:10.1016/j.celrep.2018.03.051)
Supplement: 2 [file NIHMS961949-supplement-2.pdf]

Cell Reports

Article

JMJD1B Demethylates H4R3me2s and H3K9me2 to Facilitate Gene Expression for Development of Hematopoietic Stem and Progenitor Cells

Graphical Abstract

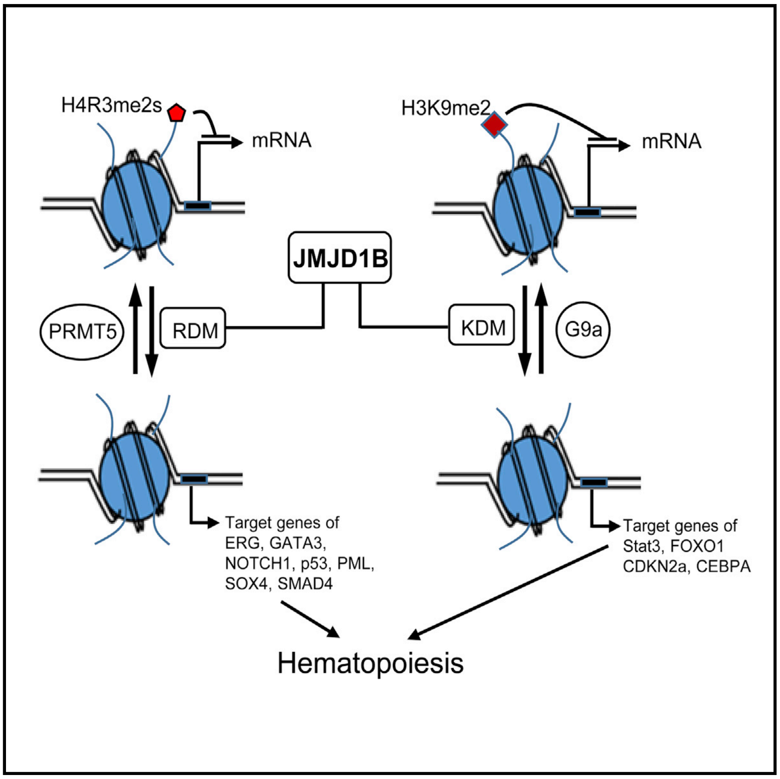

Authors

Sihui Li, Shafat Ali, Xiaotao Duan, ...,
Yanzhong Yang, Li Zheng, Binghui Shen

Correspondence

lzheng@coh.org (L.Z.),
bshen@coh.org (B.S.)

In Brief

Li et al. identify the arginine demethylase (RDM) activity of JMJD1B, a known lysine demethylase (KDM). They reveal that JMJD1B actively mediates demethylation of histone markers H4R3me2s and H3K9me2 in hematopoietic stem/progenitor cells (HSPCs).

Highlights

- JMJD1B is an arginine and lysine demethylase for H4R3me2s and H3K9me2
- JMJD1B and PRMT5 control the H4R3me2s dynamics at promoters of hematopoietic genes
- JMJD1B deficiency alters gene expression profile and impairs hematopoietic development

Data and Software Availability

GSE94966

Li et al., 2018, Cell Reports *23*, 389–403
April 10, 2018 © 2018 The Author(s).
https://doi.org/10.1016/j.celrep.2018.03.051

Cell Reports
Article

OPEN ACCESS
CellPress

JMJD1B Demethylates H4R3me2s and H3K9me2 to Facilitate Gene Expression for Development of Hematopoietic Stem and Progenitor Cells

Sihui Li,[1,2,12] Shafat Ali,[2,12] Xiaotao Duan,[3,12] Songbai Liu,[2,10] Juan Du,[4,5] Changwei Liu,[2] Huifang Dai,[2] Mian Zhou,[2] Lina Zhou,[1,2] Lu Yang,[5] Peiguo Chu,[6] Ling Li,[7] Ravi Bhatia,[7,11] Dustin E. Schones,[4] Xiwei Wu,[5] Hong Xu,[8] Yuejin Hua,[8] Zhigang Guo,[9] Yanzhong Yang,[2] Li Zheng,[2,*] and Binghui Shen[2,13,*]
[1]College of Life Sciences, Zhejiang University, Hangzhou, Zhejiang, China
[2]Departments of Cancer Genetics and Epigenetics, Beckman Research Institute, City of Hope, Duarte, CA, USA
[3]State Key Laboratory of Toxicology and Medical Countermeasures, Beijing Institute of Pharmacology and Toxicology, Beijing, China
[4]Department of Diabetes Complications & Metabolism, Beckman Research Institute, City of Hope, Duarte, CA, USA
[5]Department of Molecular and Cellular Biology, Beckman Research Institute, City of Hope, Duarte, CA, USA
[6]Department of Pathology, Beckman Research Institute, City of Hope, Duarte, CA, USA
[7]Department of Hematologic Malignancy Translational Science, Beckman Research Institute, City of Hope, Duarte, CA, USA
[8]Colleges of Life Sciences and Agriculture and Biotechnology, Zhejiang University, Hangzhou, Zhejiang, China
[9]College of Life Sciences, Nanjing Normal University, Nanjing, Jiangsu, China
[10]Present address: Suzhou Key Laboratory of Biotechnology for Laboratory Medicine, Suzhou Health College, Suzhou, Jiangsu, China
[11]Present address: School of Medicine, University of Alabama at Birmingham, Birmingham, AL 35294, USA
[12]These authors contributed equally
[13]Lead Contact
*Correspondence: lzheng@coh.org (L.Z.), bshen@coh.org (B.S.)
https://doi.org/10.1016/j.celrep.2018.03.051

SUMMARY

The arginine methylation status of histones dynamically changes during many cellular processes, including hematopoietic stem/progenitor cell (HSPC) development. The arginine methyltransferases and the readers that transduce the histone codes have been defined. However, whether arginine demethylation actively occurs in cells and what enzyme demethylates the methylarginine residues during various cellular processes are unknown. We report that JMJD1B, previously identified as a lysine demethylase for H3K9me2, mediates arginine demethylation of H4R3me2s and its intermediate, H4R3me1. We show that demethylation of H4R3me2s and H3K9me2s in promoter regions is correlated with active gene expression. Furthermore, knockout of JMJD1B blocks demethylation of H4R3me2s and/or H3K9me2 at distinct clusters of genes and impairs the activation of genes important for HSPC differentiation and development. Consequently, JMJD1B$^{-/-}$ mice show defects in hematopoiesis. Altogether, our study demonstrates that demethylase-mediated active arginine demethylation process exists in eukaryotes and that JMJD1B demethylates both H4R3me2s and H3K9me2 for epigenetic programming during hematopoiesis.

INTRODUCTION

The arginine methylation status of core histones dynamically changes during many essential cellular processes, particularly during embryonic and hematopoietic stem cell (HSC) development (Bedford and Clarke, 2009; Blanc and Richard, 2017; Chen et al., 2011; Yang and Bedford, 2013). There are nine arginine methyltransferase (PRMT) genes encoded in the human genome, and their protein products catalyze the methylation reaction in three different forms: ω-NG-monomethylarginine (MMA, me1), ω-NG,NG-asymmetric dimethylarginine (ADMA, me2a), and ω-NG,N′G-symmetric dimethylarginine (SDMA, me2s) (Fuhrmann et al., 2015; Yang and Bedford, 2013). These modifications often serve as docking sites for Tudor domain-containing effector molecules, which transduce post-translational modification signals to produce biological outcomes (Chen et al., 2011). Accumulating evidence suggests that arginine methylation is dynamically regulated in cells during transcription (Le Romancer et al., 2008; Sims et al., 2011), replication (Guo et al., 2010), DNA damage response (Jansson et al., 2008), and cell cycle progression (Guo et al., 2010; Yu et al., 2009). Accordant with this, dysregulation of histone arginine methylation has been linked to many human diseases, including neurological disorders, autoimmunity, and cancer (Yang and Bedford, 2013).

Although arginine methyltransferases have been identified and their function in cells has been well documented (Fuhrmann et al., 2015; Yang and Bedford, 2013), arginine demethylases have not yet been identified. JMJD6 (JmjC domain containing 6) was previously reported as a putative arginine demethylase for ADMA and SDMA histone substrates (Chang et al., 2007).

This is an open access article under the CC BY license (http://creativecommons.org/licenses/by/4.0/).

However, additional structural and functional studies showed that lysine, rather than arginine, is the substrate of JMJD6 (Mantri et al., 2010; Webby et al., 2009). More recently, a study showed that certain lysine demethylases possess arginine demethylase activity on methylated histone peptide model substrates (Walport et al., 2016). However, an arginine demethylase with a biological role has not been discovered *in vivo* (Blanc and Richard, 2017; Greer and Shi, 2012; Yang and Bedford, 2013). Identification of the histone arginine demethylase will not only fill a fundamental gap in our understanding of the field of epigenetics but also provide additional targets for therapeutic regimens.

Here, we report that JMJD1B possesses arginine demethylase activity on H4R3me2s. We establish that the demethylation of H4R3me2s in promoter and gene body regions is inversely correlated with gene expression in hematopoietic stem/progenitor cells (HSPCs). Furthermore, we observed that knockout of JMJD1B causes defects in demethylation of H4R3me2s, leading to downregulation of genes important for hematopoietic cell differentiation and development. Consequently, JMJD1B$^{-/-}$ mice display defective hematopoiesis, showing moderate anemia and remarkable leukocytosis phenotypes. Altogether, our study demonstrates that arginine demethylases exist in cellular systems and that JMJD1B demethylates H4R3me2s for proper epigenetic programming during development.

RESULTS

JMJD1B Protein Possesses Both Lysine and Arginine Demethylase Activities

JMJD1B, which is a typical JmjC domain-containing protein, was previously characterized as one of the lysine demethylases targeting H3K9 dimethylation (Kim et al., 2012; Mikhaleva et al., 2011). We recently found that flap endonuclease 1 (FEN1), which undergoes SDMA at the R192 residue in S phase or in response to DNA damage (Guo et al., 2010), is in complex with JMJD1B. This prompted us to hypothesize that JMJD1B may be an arginine demethylase acting on both histone and non-histone proteins. To determine whether JMJD1B is a histone arginine demethylase, we expressed and purified FLAG-tagged full-length wild-type (WT) JMJD1B (isoform 1) or a catalytically inactive JMJD1B mutant (mut), which carries mutations in the conserved cofactor (Fe^{2+}) binding site (H1560A/D1562A/H1689A) (purity > 90%; Figure S1A). We validated the JMJD1B lysine demethylase activity toward H3K9me2 (Figure 1A) and also evaluated arginine demethylase activity toward H3 and H4, which are known methylation targets (Huang et al., 2014). With the synthetic histone peptides and antibodies (Table S1) against a specific form and histone modification, whose specificity was verified (Figure S1B), we conducted dot-blot-based *in vitro* demethylation assays in the absence or presence of recombinant JMJD1B. JMJD1B had no activity toward the synthesized lysine-methylated and arginine-methylated histone peptides, including H3R2me1, H3R2me2a, H3R2me2s, H3R8me1, H3R8me2s, H3R17me2a, H3R26me2a, H4R3me2a, and H3K4me2. In contrast, JMJD1B exhibited demethylase activity specifically toward H3K9me2, H4R3me1, and H4R3me2s peptides (Figure 1A). We next performed *in vitro* demethylation assays using bulk histones purified from HEK293T cells as substrates and confirmed that JMJD1B preferentially demethylated H3K9me2, H4R3me1, and H4R3me2s (Figure 1B). Mutant JMJD1B showed no activity toward H3K9me2, H4R3me2s, or H4R3me1 (Figure 1C). For optimal activity, lysine demethylases require three cofactors: Fe^{2+}, α-ketoglutarate, and ascorbate (Klose et al., 2006; Shi et al., 2004). These three cofactors were also required for the arginine demethylase activity (Figure 1D). Together, these data support a model in which JMJD1B demethylates both lysine (H3K9me2) and arginine (H4R3me2s and H4R3me1) residues.

To confirm the arginine demethylase activity of JMJD1B, we conducted *in vitro* demethylation using purified WT or mutant JMJD1B, and used Fourier-transform ion cyclotron resonance (FT-ICR) mass spectrometry to analyze the reaction products. Synthetic histone H4 tail peptide H4R3me1, H4R3me2s, and H4R3me2a were used as arginine demethylation substrates (Table S2). The known JMJD1B lysine demethylation substrate, H3K9me2, was used as a positive control (Figures S2A–S2C). In the absence of JMJD1B, we observed peaks in the H4R3me2s substrate at 1,443 and 1,465 mass-to-charge ratio (m/z), which correspond to the H4R3me2s peptide molecular ion plus a proton ($[M+H]^+$) and the molecular ion plus Na^+ ($[M+Na]^+$), respectively (Figure 2A). After incubating JMJD1B with the H4R3me2s peptide substrate for 2 hr, the intensity of these two peaks was reduced, and two new peaks appeared, which were shifted 14 Da from the 1,443 ($[M+H]^+$) peak and the 1,465 ($[M+Na]^+$) peak, corresponding to the molecular ions of H4R3me1 (Figure 2B). As we increased the incubation time to 4 hr, we observed an additional peak with a shift of 28 Da from the original $[M+H]^+$ or $[M+Na]^+$ peak, which corresponds to the molecular ion of the non-modified H4 tail peptide (Figure 2C). In contrast, incubating mutant JMJD1B with the H4R3me2s substrate did not resulted in any peak shift (Figure 2D). Similarly, in the absence of JMJD1B, we observed $[M+H]^+$ and $[M+Na]^+$ peaks in the H4R3me1 substrate (Figure 2E). Addition of WT JMJD1B but not mutant JMJD1B resulted in peaks that were shifted 14 Da from the original $[M+H]^+$ and $[M+Na]^+$ peaks (Figures 2F and 2G), corresponding to the molecular ion of the non-modified H4 tail peptide. The mass spectrometry revealed no arginine demethylation products when WT or mutant JMJD1B was incubated with the H4R3me2a substrate (Figures S2D–S2F). Altogether, these biochemical data indicate that JMJD1B specifically demethylates H4R3me2s but not H4R3me2a.

The Rate of JMJD1B Arginine Demethylase Activity Is Similar to That of Its Lysine Demethylase Activity *In Vitro*

We determined the kinetics of JMJD1B-mediated arginine demethylation of H4R3me2s. We first evaluated the intermediates of the JMJD1B-mediated arginine demethylation reaction and found that the amount of H4R3me2s decreased with time (Figure 3A). In contrast, the H4R3me1 level first increased, and after reaching its maximum at 20 min, it started to decrease. This is consistent with a model in which H4R3me2s is the substrate of JMJD1B, and H4R3me1 is an intermediate in the JMJD1B-mediated demethylation of H4R3me2s. The rate of release of the methyl group from H4R3me2s was further measured by the release of formaldehyde, which showed that the JMJD1B-catalyzed H4R3me2s demethylation rate was slightly lower than the demethylation rate for H3K9me2 (Figure 3B). The preliminary

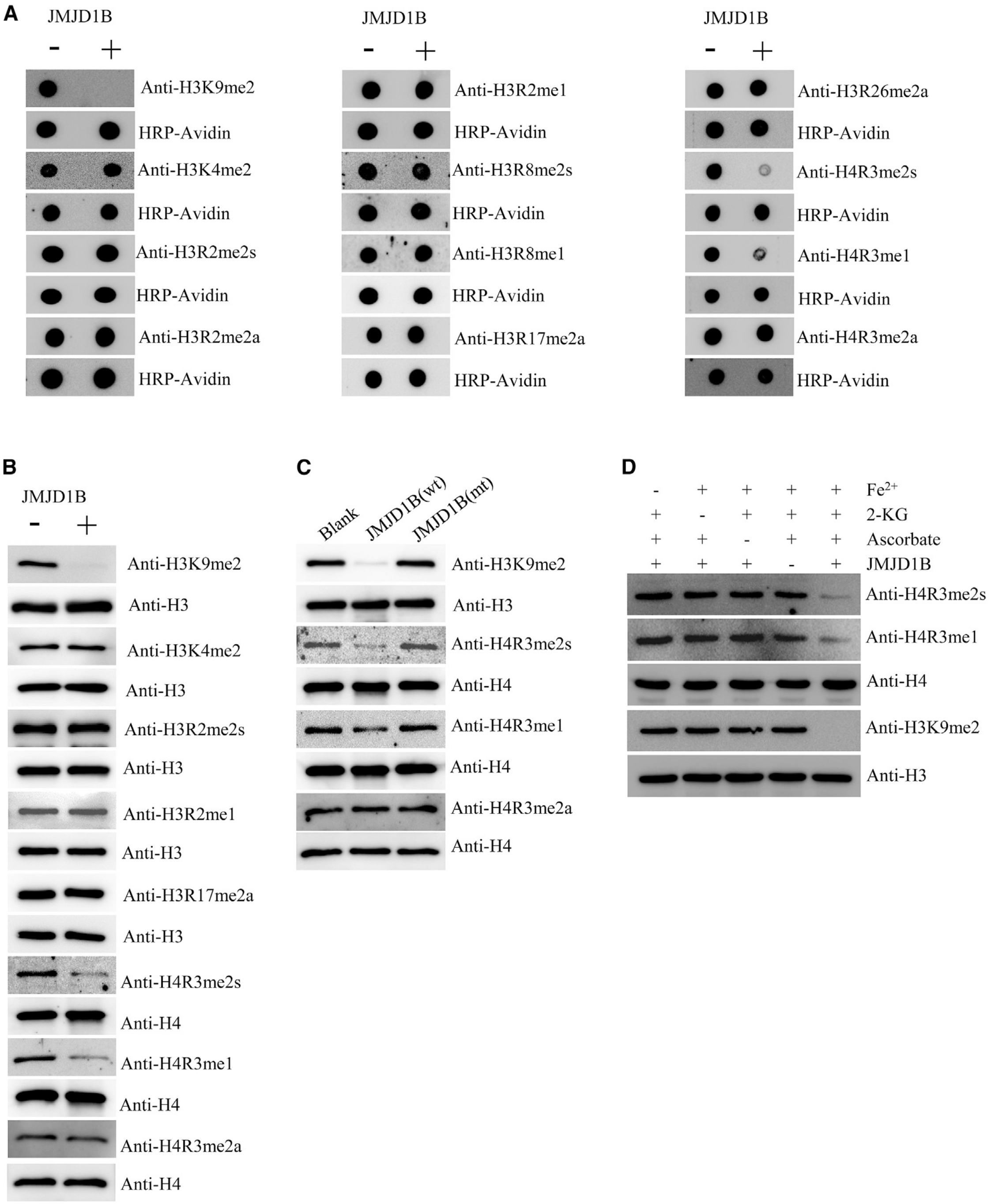

Figure 1. JMJD1B Protein Possesses Both Lysine and Arginine Demethylase Activities *In Vitro*

(A) Synthetic histone tails (22–25 amino acid residues, 18 μM) were incubated in a demethylation buffer (50 mM HEPES-KOH [pH 7.5], 1 mM 2-KG, 2 mM ascorbate, 1 mM TCEP, 500 μM $[NH_4]_2Fe[SO_4]_2 \cdot 6H_2O$) in the absence (-) or presence (+) of purified recombinant JMJD1B (0.25 μM) at 37°C for 2 hr and analyzed using dot blot with indicated antibodies. Blots were stripped and reprobed for HRP-avidin as loading control.

(B) Bulk histones (5 μg) were incubated in a demethylation buffer as in (A) in the absence (-) or presence (+) of purified recombinant JMJD1B (0.25 μM) at 37°C for 2 hr and analyzed using western blotting. Blots were stripped and reprobed for total levels of H3 and H4 as loading controls.

(C) Bulk histones (5 μg) were incubated in absence (Blank) or presence of WT JMJD1B (wt; 0.25 μM) or a catalytically inactive mutant (mut; JMJD1B [H1560A/D1562A/H1689A], 0.25 μM) at 37°C for 2 and 4 hr and analyzed using western blotting. The total level of H4 was used as an internal loading control.

(D) Three cofactors, Fe^{2+}, 2-KG, and ascorbate, are required for the full enzyme activity to demethylate both H4R3me2s and H3K9me2. The same reaction conditions as in (B) and (C) were used.

See also Table S1 and Figure S1.

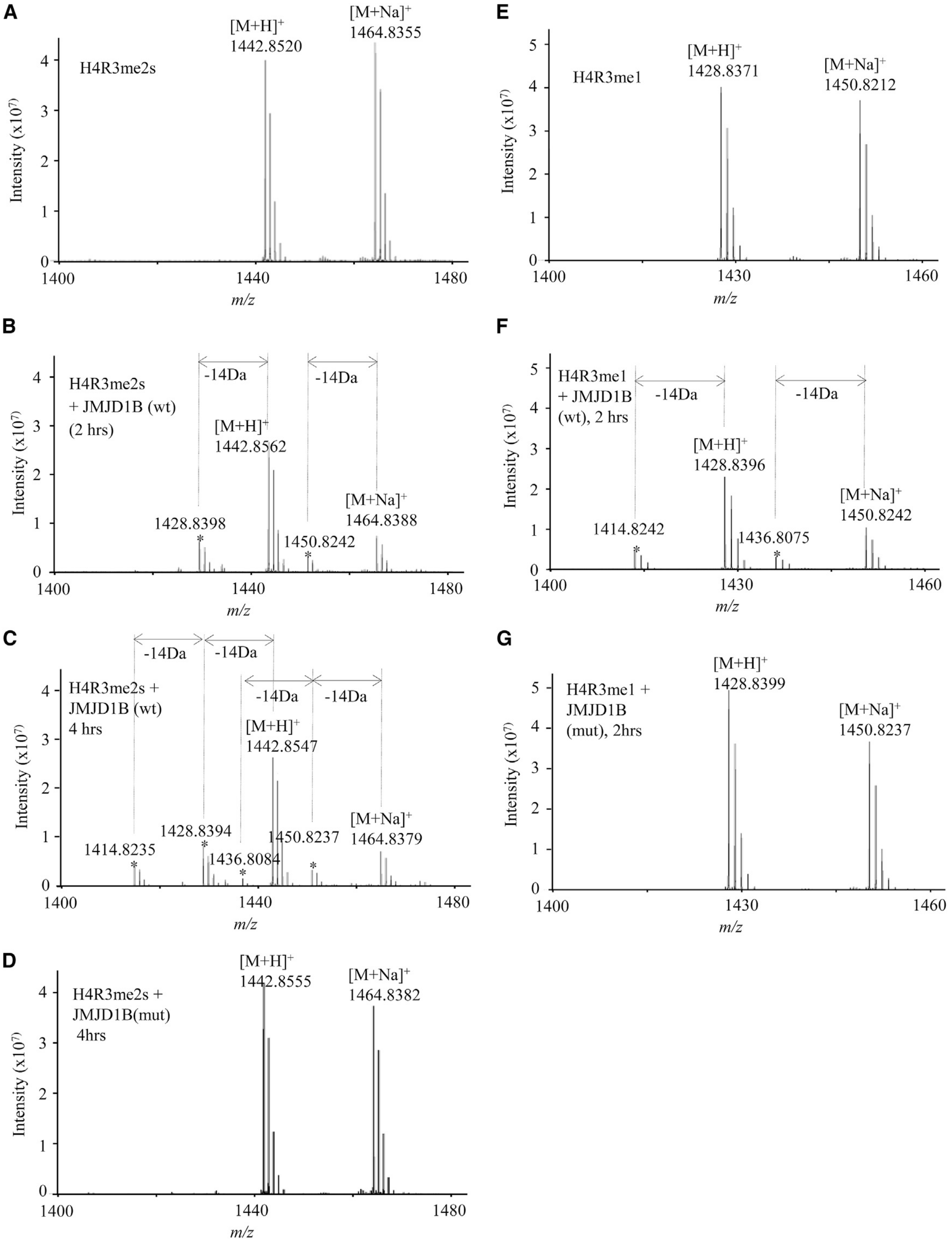

Figure 2. Mass Spectrometry Confirms that JMJD1B Demethylates H4R3me2s and H4R3me1 Peptide Model Substrates

(A–D) Representative mass spectra for H4R3me2s demethylation by JMJD1B. The H4R3me2s 16 aa peptide substrate (34 μM) was incubated with buffer only (α-ketoglutarate [2-KG, 1 mM], ascorbate [2 mM], and Fe^{2+} [500 μM]) for 4 hr (A), with wild-type enzyme (JMJD1B [wt]; 0.5 μM) at 37°C for 2 hr (B) and 4 hr (C), or with inactive mutant enzyme for 4 hr (JMJD1B [mutant]; 0.5 μM) (D). Asterisk indicates products from the H4R3me2s peptide (B and C).

(E–G) Representative mass spectra for H4R3me1 demethylation by JMJD1B. The H4R3me1 peptide substrate (34 μM) was incubated with demethylation buffer (E), 0.5 μM JMJD1B (wt) (F), or 0.5 μM JMJD1B (mutant) (G) at 37°C for 2 hr. Asterisk indicates the demethylation products.

See also Table S2 and Figure S2.

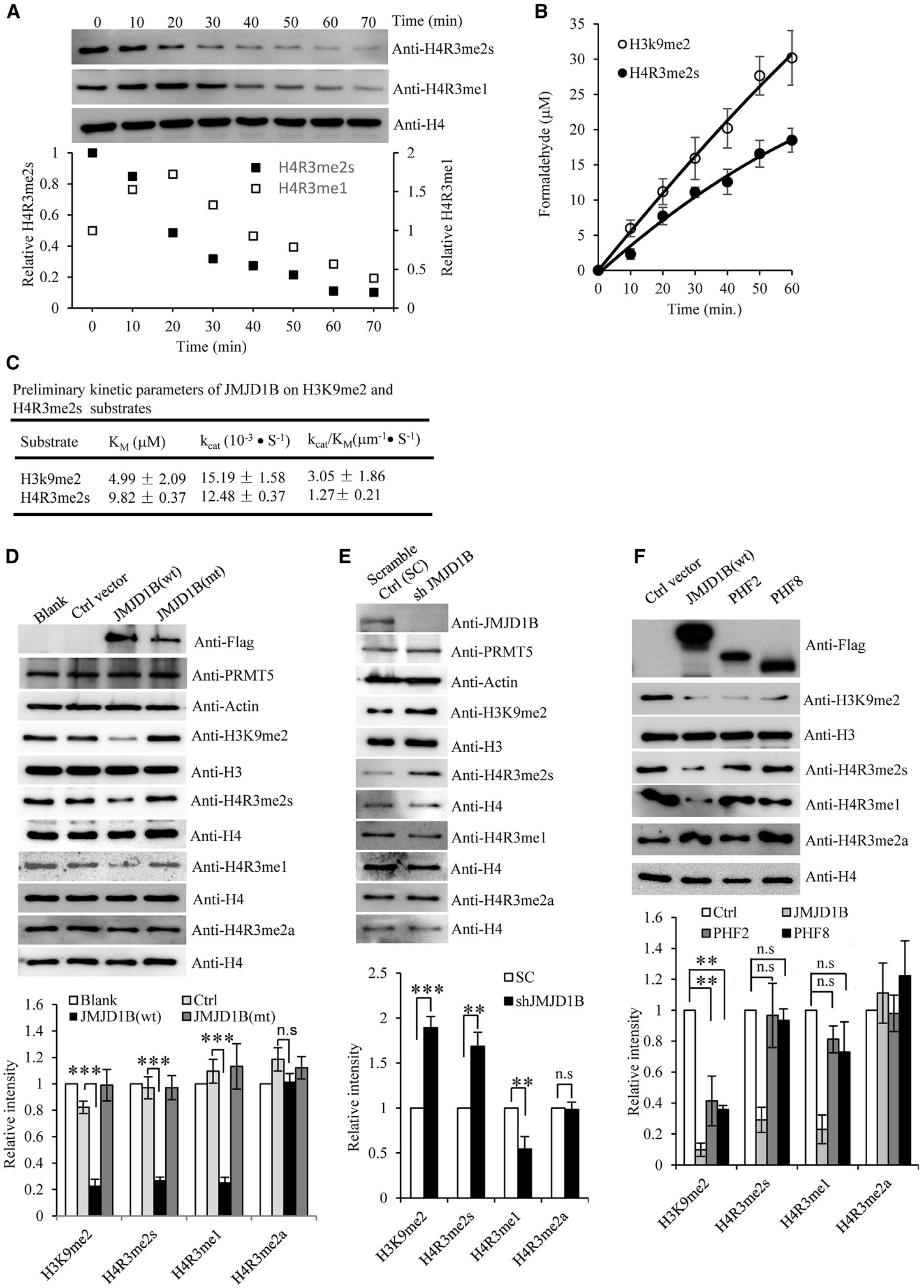

Figure 3. JMJD1B Demethylates Histone H4R3me2s and H3K9me2 *In Vitro* and in Cells

(A) Time dependence of JMJD1B histone arginine demethylase activity. Bulk histones (5 μg) were incubated with JMJD1B (0.5 μM) in the demethylation at 37°C for 0–70 min, as indicated, and analyzed by western blotting. Top: representative western blotting images; bottom: semi-quantification of H4R3me2s and

(legend continued on next page)

kinetic parameters suggested that although purified recombinant JMJD1B has a similar turnover rate on the H3K9me2 and H4R3me2s peptide substrates (i.e., k_{cat} = 15.19 and 12.48), it had higher affinity for the H3K9me2 peptide substrate than the H4R3me2s peptide substrate (i.e., K_m = 4.99 μM versus 9.82 μM; Figure 3C).

Cellular Level of JMJD1B Corresponds to H4R3me2s and H3K9me2 Methylation Status

To determine if JMJD1B mediates arginine demethylation of H4R3me2s and H4R3me1 in cells, we overexpressed WT or mutant JMJD1B in HEK293T cells and measured H4R3me2s and H4R3me1 levels. As anticipated, overexpression of WT, but not mutant, JMJD1B significantly reduced the H3K9me2, H4R3me2s, and H4R3me1 levels, compared with parental control cells (Figure 3D). However, overexpression of JMJD1B did not affect the H4R3me2a level (Figure 3D). Conversely, knockdown of JMJD1B (Figure S1C) resulted in H3K9me2 and H4R3me2s accumulation in the cells (Figure 3E). The H4R3me1 level was reduced in JMJD1B-knockdown cells (Figure 3E), consistent with a model in which H4R3me1 is an intermediate of JMJD1B-mediated H4R3me2s demethylation. To demonstrate that JMJD1B does not affect H4R3me2s via regulation of PRMT5, we assessed the level of PRMT5 in JMJD1B-overexpressing or JMJD1B-knockdown cells; we noted little change compared with control cells (Figures 3D and 3E). To determine whether other H3K9me2 demethylases could also catalyze arginine demethylation, we overexpressed PHF2 and PHF8, which have been reported to show H3K9me2 demethylase activity (Lee et al., 2014; Qi et al., 2010). Overexpression of PHF2 or PHF8 reduced the level of H3K9me2, as expected, but did not change the level of H4R3me2s, H4R3me1, or H4R3me2a (Figure 3F). This is consistent with the possibility that JMJD1B is a unique H4R3me2s and H4R3me1 demethylase.

Highly Expressed JMJD1B Maintains the Demethylation Status of H3K9me2 and H4R3me2s in HSPCs

Human JMJD1B is highly expressed in hematopoietic cells, retinal tissue, and pineal glands (http://biogps.org), suggesting that it plays a role in development and/or maintenance of proper cellular functions in these cells. Similar to human hematopoietic cells, mouse HSPCs also expressed elevated JMJD1B (Figure 4A) compared with other cell types. In contrast, JMJD1B expression reduced significantly in mature bone marrow cells (BMCs) such as bone marrow neutrophils (Figure 4A). Consistently, HSPCs had relatively low levels of H4R3me2s and H3K9me2 compared with BMCs (Figure 4B).

We then determined if high JMJD1B expression is associated with low H4R3me2s and H3K9me2 density in HSPCs. We performed MACS2 analysis of native chromatin immunoprecipitation-DNA sequencing (ChIP-seq) on H3K9me2 and H4R3me2s and identified 9,822 and 622 H3K9me2 and H4R3me2s peaks ($p < 0.0001$) in BMCs (Figure 4C). In contrast, H3K9me2 and H4R3me2s had only 1 and 28 significantly enriched peaks in HSPCs, respectively (Figure 4C). Real-time PCR on representative genomic loci confirmed that in many genomic loci, H4R3me2s and H3K9me2 occupancy in HSPCs was much lower than in BMCs (Figure 4D). These findings suggest that during hematopoiesis, H3K9me2s and H4R3me2s histone markers are not established at most loci in HSPCs. This is consistent with previous findings that the H3K9me2 marker is not established in human HSCs (Chen et al., 2012; Schones et al., 2014). In addition, both PRMT5 and G9a (EHMT2), the methyltransferases for H4R3me2s and H3K9me2 respectively were actively expressed (log_2RPKM [reads per kilobase of transcript per million mapped reads] > 4). Thus, the non-establishment status of H4R3me2s and H3K9me2 in HSPCs is likely due to the activity of demethylases, rather than low levels of methyltransferases.

Even though the currently available software was not able to call peaks from the ChIP-seq data in HSPCs, there are significant differences in the H3K9me2 and H4R3me2s occupancy densities across the genome. To determine the impact of H3K9me2 and H4R3me2s density on gene expression in HSPCs, we conducted RNA sequencing (RNA-seq) and analyzed the relationship between the density of these two histone markers and the levels of gene expression. We categorized genes across the genome into clusters on the basis of gene expression levels and compared them with the H4R3me2s and H3K9me2 occupancy density levels across the gene bodies

H4R3me1levels. The H4R3me1 and H4R3me2s levels were normalized with corresponding total H4 levels. The normalized H4R3me1 and H4R3me2s levels at 0 hr were arbitrarily set as 1. The relative levels of H4R3me1 and H4R3me2s at other time points were calculated relative to the value at 0 min. Values are the average of two independent assays.

(B) Time-dependent formaldehyde release in JMJD1B-mediated demethylation of H4R3me2s and H3K9me2 peptide substrates. The H4R3me2s or H3K9me2 peptide substrates (50 μM) were incubated with JMJD1B (0.5 μM) in the demethylation buffer at 37°C, and the release of formaldehyde was measured at 0, 10, 20, 30, 40, 50, and 60 min. Values are mean ± SEM of three independent assays.

(C) Preliminary kinetic parameters of JMJD1B-mediated demethylation of H4R3me2s or H3K9me2. The formaldehyde release assay on different substrate concentrations was used to generate K_m, k_{cat}, and k_{cat}/K_m. Values are mean ± SEM of three independent assays.

(D–F) Alteration of JMJD1B expression level corresponds to H4R3me2s and H4R3me1 demethylation status in HEK293T cells.

(D) JMJD1B was expressed in HEK293T cells as a FLAG fusion protein, and histones were analyzed by western blotting with antibodies against the indicated modifications. Cells with no transfection (Blank) or transfected with an empty vector (Ctrl) were used as controls.

(E) Stable JMJD1B-knockdown cells (Sh-JMJD1B) were lysed and immuno-blotted with the indicated antibodies.

(F) JMJD1B, PHF2, and PHF8 were expressed in HEK293T cells, and histones were analyzed by western blotting with antibodies against the indicated modifications. Histone H3 and H4 were used as a loading control in all panels. In each panel, the upper section shows the representative western blotting images, and the lower section shows the semi-quantification of western blotting (i.e., the relative intensities of H4R3me2s, H4R3me1, and H3K9me2 bands). Band intensity was normalized to corresponding loading control (histone H3 or H4).

The normalized H4R3me2s, H4R3me1, or H3K9me2 levels in the cells with no transfection, or transfected with control short hairpin RNA (shRNA) (SC) or empty vector were arbitrarily set as 1 in (D), (E), and (F), respectively. The relative intensity was calculated by comparing the normalized band intensity of each sample to that of the control. Values are means ± SEM of three independent assays. *$p < 0.05$, **$p < 0.01$, and ***$p < 0.0001$ (Student's t test). See also Figure S1.

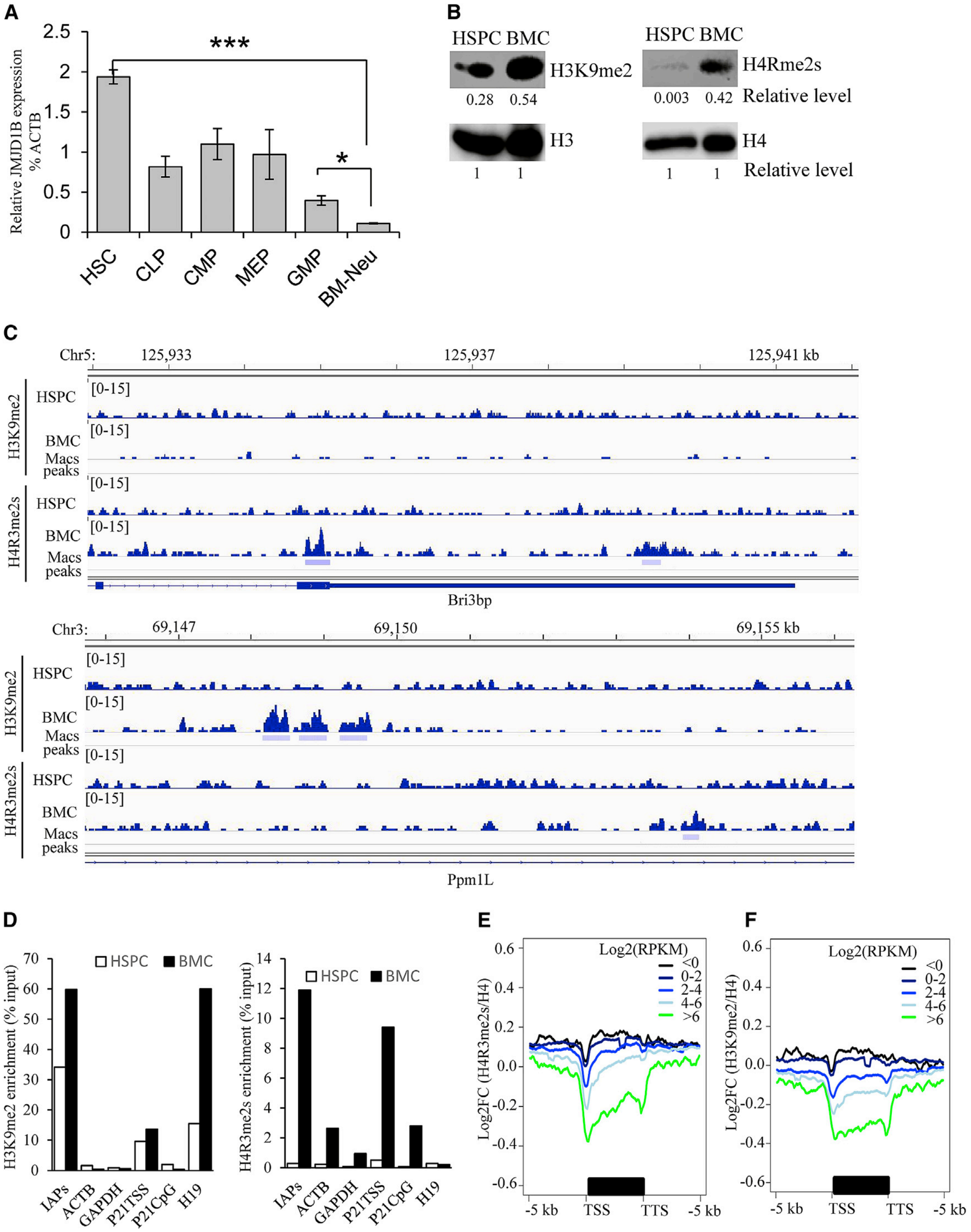

Figure 4. Elevated JMJD1B Expression Maintains the Demethylation Status of H3K9me2 and H4R3me2s in HSPCs

(A) JMJD1B gene expression in hematopoietic stem cells and various progenitors, BMCs, and blood cells. HSC, hematopoietic stem cell; CLP, common lymphoid progenitor; CMP, common myeloid progenitor; MEP, megakaryocyte-erythrocyte progenitor; GMP, granulocyte-monocytes progenitor; BM-Neu, bone marrow neutrophils. JMJD1B and the house keeping gene β-actin (ATCB) were measured using real-time RT-PCR. The relative levels of JMJD1B mRNA were calculated by comparing it with the level corresponding β-actin mRNA, which is arbitrarily set as 100, in each type of cell. Values are mean ± SEM of three mice. *$p < 0.05$, **$p < 0.01$, and ***$p < 0.001$ (Student's t test).

(B) Total chromatin-associated histone H3 and H4, H4R3me2s, and H3K9me2 in WT HSPCs and BMCs were detected by western blot. The intensity of each band was semi-quantified using ImageJ. The intensity of histone H3 or H4 for each sample was arbitrarily set as 1, and the relative level of H4R3me2s or H3K9me2 in HSPCs or BMCs was calculated by comparing their intensity with corresponding H3 or H4 control.

(legend continued on next page)

(transcription start site [TSS] ± 5 kb). The aggregate plots showed an inverse correlation of H4R3me2s and H3K9me2 occupancy and gene expression levels: the reduction of H4R3me2s or H3K9me2 occupancy was associated with an increase in gene expression (Figures 4E and 4F). This suggests that maintaining the demethylation status of the suppressive markers H4R3me2s and H3K9me2 is important for induction of gene expression in HSPCs.

JMJD1B Demethylates H4R3me2s and H3K9me2 to Regulate Genes Important for HSPC Survival, Proliferation, and Differentiation

A key question is whether JMJD1B plays an active role in maintaining H4R3me2s and H3K9me2 markers at low levels for gene expression in HSPCs. To this aim, we established JMJD1B knockout (JMJD1B$^{-/-}$; JKO) mice (Figure S3), using validated JMJD1B$^{-/-}$ embryonic stem cells (129P2 genetic background) from the European Mouse Mutant Cell Repository (EuMMCR). We performed H4R3me2s and H3K9me2 ChIP-seq experiments using HSPCs that were purified from the bone marrow of age-matched WT and JMJD1B$^{-/-}$ littermates. We then analyzed the H4R3me2s and H3K9me2 ChIP signals at the promoter region (TSS –1 kb upstream of TSS), and normalized them to corresponding H4 ChIP signals at the same region across the genome in the WT and JMJD1B$^{-/-}$ HSPCs. On the basis of the status of H3K9me2 or H4Rme2s signals relative to H4 in WT HSPCs, we divided the genes across the genome into four groups: the 2-fold or more depletion group (log_2 fold change [FC] [H3K9me2 or H4R3me2s over H4] ≤ -1), the 1- to 2-fold depletion group ($-1 <$ log_2FC [H3K9me2 or H4R3me2s over H4] $\leq$ 0), the 1- to 2-fold enrichment group (0 $<$ log_2FC [H3K9me2 or H4R3me2s over H4] $\leq$ 1), and the 2-fold or more enrichment group (0 $<$ log_2FC [H3K9me2 or H4R3me2s over H4]). We found that JMJD1B knockout led to a significant increase (FC of JMJD1B$^{-/-}$ over WT $\geq$ 1.5, $p < 0.05$) in the mean density of H4R3me2s and H3K9me2 in the genes of the 2-fold or more depletion group (Figure 5A). qPCR analysis confirmed that JMJD1B knockout significantly increased the H4R3me2s signals at H4R3m2s-depleted genes (Figure 5B) but not in the non-depleted genes (Figure 5C).

Figure 5D shows a representative gene, NOTCH1, with depletion (in green) of H3K9me2 and H4R3me2 at the promoter region in WT HSPCs; in contrast, JMJD1B knockout resulted in significant gain (in red) of H4R3me2s and H3K9me2 signals. Similar to the NOTCH1 gene, 2,421 and 3,001 genes, respectively, had significant H3K9me2 or H4R3me2s occupancy density increases, which we defined as JMJD1B knockout ChIP reads $\geq$ 15 and the FC of JMJD1B$^{-/-}$ over WT $\geq$ 1.5 at the promoter regions. The identified genes with gains in H4R3me2s and/or H3K9me2 ChIP signals could be categorized into three types on the basis of the methylation markers in JMJD1B$^{-/-}$ versus WT HSPCs: (1) 967 genes showed a $\geq$1.5-fold increase in both H4R3me2s and H3K9me2 density, (2) 1,454 genes had a $\geq$1.5-fold increase in H3K9me2 but not H4R3me2s, and (3) 2,034 genes displayed a $\geq$1.5-fold increase in H4R3me2s but not H3K9me2. Therefore, these two genetic markers influence gene expression distinctively. Of note, JMJD1B$^{-/-}$ did not change the level of H3K9me2/H4R3me2s at PRMTs or G9a loci or methyltransferase gene expression levels (Figures S4A and S4B). Thus, the elevation of H4R3me2s or H3K9me2 level in the JMJD1B$^{-/-}$ HSPCs is likely not due to change of protein methyl transferase activities.

We sought to define the genes, corresponding pathways, and cellular functions for hematopoiesis that are controlled by JMJD1B-mediated demethylation of H4R3me2s and H3K9me2 in HSPCs for hematopoiesis. RNA-seq analysis revealed that 937 genes with statistically decreased expression ($p < 0.05$) and 444 genes with statistically increased expression ($p < 0.05$) in JMJD1B$^{-/-}$ HSPCs, compared with the WT (Figures S4C–S4E). We characterized the intersection of genes with increases in H4R3me2s or H3K9me2 markers and the genes with altered expression (downregulation or upregulation) in JMJD1B$^{-/-}$ HSPCs. A total of 140 and 99 genes, respectively, of relatively high H4R3me2s and H3K9me2 density were downregulated genes (Figure S4D). Fifty-six and 40 genes, respectively, of relatively high H4R3me2s or H3K9me2 genes were upregulated genes in JMJD1B$^{-/-}$ HSPCs (Figure S4E). Considering H4R3me2s and H3K9me2 are repressive markers, it is likely that H4R3me2s and H3K9me2 might inhibit the expression of inhibitory transcription regulators, leading to upregulation of the target genes of the transcription repressors. In addition, JMJD1B knockout might also affect the activity of non-histone proteins such as transcript factors, leading to relatively complex gene expression alterations.

Next, we determined what transcription factors are affected by JMJD1B-mediated demethylation of H4R3me2s or H3K9me2, leading to downregulation or upregulation of the genes in JMJD1B$^{-/-}$ HSPCs. Ingenuity Pathway Analysis (IPA) causal analysis on these overlapped genes from the intersection of the RNA-seq and ChIP-seq datasets predicted that an increase in H4R3me2s occupancy due to JMJD1B knockout specifically affected the target genes of CTNNB1, ERG, SOX2, FOS, GATA3, and PML signaling pathways for hematopoiesis (Ito et al., 2012; Ku et al., 2012; Liebermann et al., 1998; Ng et al., 2011; Sarkar and Hochedlinger, 2013; Scheller et al., 2006) (Figure 5E), while the increase in H3K9me2 by JMJD1B knockout was predicted to affect expression of target genes of CDKN2a, STAT3, SMAD3, WT1, and FOXO1 for regulating proper hematopoietic processes (Chung et al., 2006; Cunningham et al., 2013; Larsson and Karlsson, 2005; Liu et al., 2009; Tothova et al., 2007) (Figure 5E). Both H4R3me2s and H3K9me2 affected NOTCH1 and SOX4, which are critical for

(C) IGV views of ChIP-seq show representative enriched H3K9me2 and H4R3me2s peaks ($p < 0.0001$) in BMCs but not in HSPCs.
(D) ChIP-qPCR for verifying H4R3me2s and H3K9me2 occupancy at selected gene loci. The ChIPed signals were normalized with corresponding input and were expressed as percentage of input. Values are the mean of three ChIP experiments.
(E and F) Aggregate plot of H4R3me2s (E) or H3K9m2 (F) abundance (Log_2FC[ChIP/ChIP H4]) and gene expression levels (log[RPKM+0.1]) for WT HSPCs. TSS, transcription start site; TTS, transcription termination site.
See also Table S3.

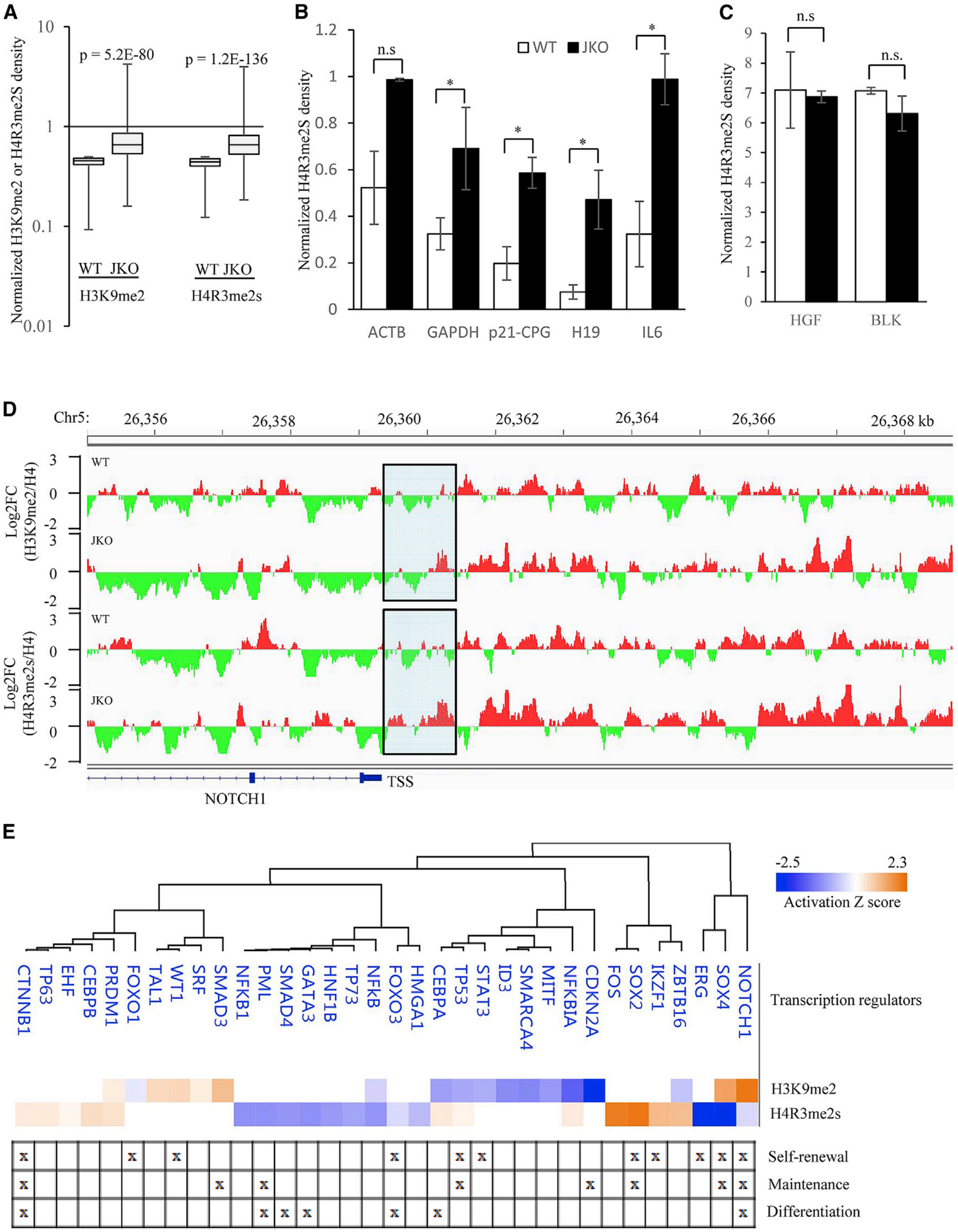

Figure 5. JMJD1B Maintains Demethylation Status of H4R3me2s and H3K9me2 at the Promoter Regions of Distinct Clusters of Genes in HSPCs for Hematopoiesis

(A) Box-and-whisker plots of H3K9me2/H4 and H4R3me2s/H4 signal changes between the mutant and WT HSPCs at the promoter regions of genes that are depleted of H3K9me2 or H4R3me2s in the WT HSPCs. The depleted genes are defined as those with $\log_2FC$ (H3K9me2/H4) or $\log_2FC$ (H4R3me2s/H4) ≤ -1 in the WT HSPCs.

(B and C) ChIP-qPCR measurement of H4R3me2s occupancy at H4R3me2s-depleted genes (B) and the non-depleted genes (C) in WT and JMJD1B$^{-/-}$ cells. The ChIPed H4R3me2s signal was normalized with the corresponding ChIPed H4 signal and was expressed as percentage of ChIPed H4. Values are mean ± SEM of three ChIP experiments. *$p < 0.05$ (Student's t test).

(legend continued on next page)

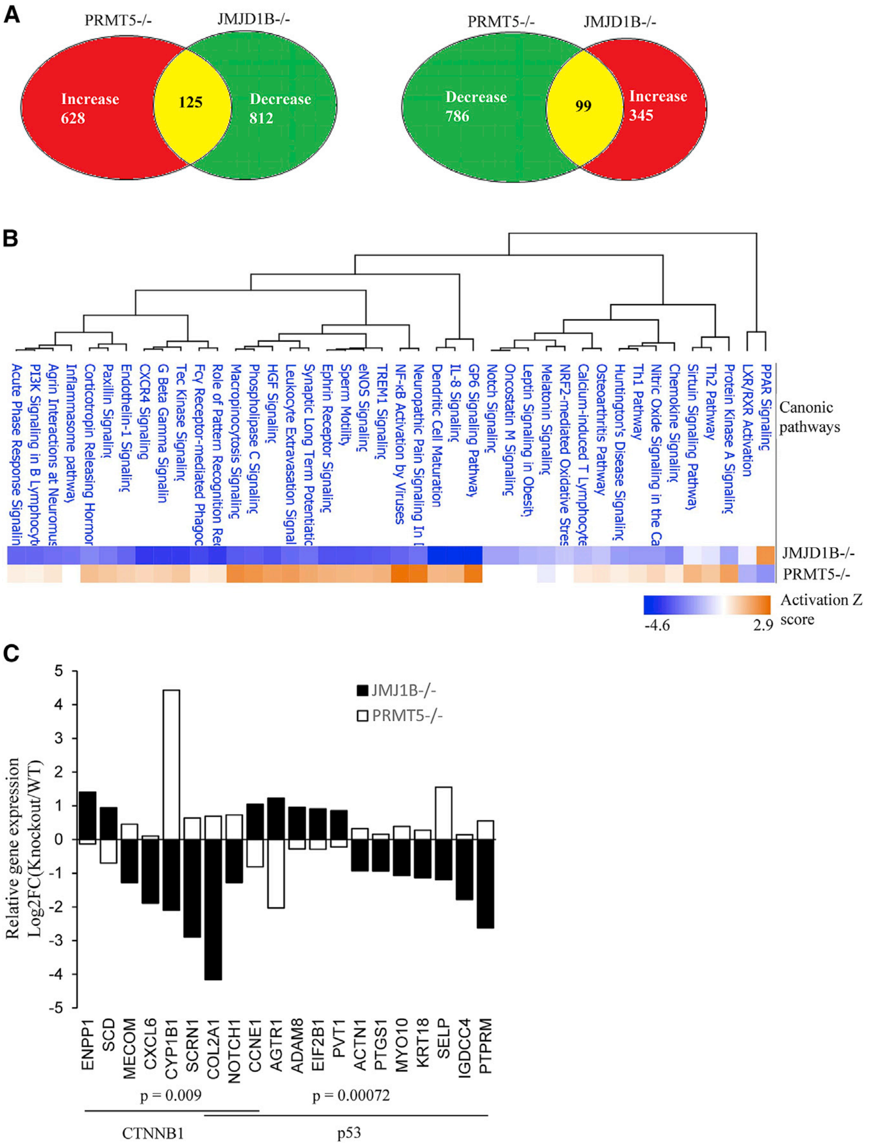

Figure 6. JMJD1B and PRMT5 Oppositely Regulate Genes Important for Hematopoietic Development

(A) Number of genes with statistically significant changes ($p < 0.05$) in JMJD1B or PRMT5 knockout HSPCs, compared with corresponding WT control. The PRMT5 RNA-seq dataset was download from the GEO database (GEO: GSE69937) (Liu et al., 2015a).

(B) IPA to identify the canonic pathways that are altered in JMJD1B and PRMT5 knockout cells. The canonic pathway with $p < 0.01$ and a calculated Z score are shown.

(C) Top predicted transcription factor signaling and corresponding affected genes that were of relatively high H4R3me2s density ($\log_2$FC [JMJD1B knockout ChIP/WT ChIP] $\geq$ 1.5) in JMJD1B$^{-/-}$ HSPCs and overlapped with genes whose expression was oppositely regulated by JMJD1B and PRMT5.

(D) Representative IGV view of the H4R3me2s and H3K9me2 ChIP signals normalized to the H4 ChIP signal in the promoter region of a depleted gene (e.g., NOTCH1 gene). The promoter region was defined as the region from the transcription start site (TSS) to 1 kb upstream of the TSS (highlighted). The depleted or enriched regions are specified by green or red color.

(E) IPA upstream analysis predicts different transcription factors corresponding to the overlapped genes as defined in (D). The transcription factors with $p < 0.01$ and a calculated Z score are shown. The roles of different transcription factors in the self-renewal, maintenance, and differentiation are specified.

See also Figures S3 and S4.

hematopoiesis (Sarkar and Hochedlinger, 2013; Stier et al., 2002).

JMJD1B and PRMT5 Work Cooperatively to Regulate the Dynamics of H4R3me2s and Hematopoietic Gene Expression

Given that JMJD1B is an arginine demethylase and PRMT5 is the only identified methyltransferase for SDMA on H4R3, it is plausible to hypothesize that they work in opposing fashion to regulate the dynamics of H4R3me2s and resultant gene expression. Thus, we expected that their knockout would have opposite effects on expression of at least certain genes. Supporting this hypothesis, expression of 125 genes increased ($p < 0.05$) in PRMT5$^{-/-}$ HSPCs but decreased ($p < 0.05$) in JMJD1B$^{-/-}$ HSPCs (Figure 6A). On the other hand, expression of 99 genes decreased ($p < 0.05$) in PRMT5$^{-/-}$ HSPCs but increased ($p < 0.05$) in JMJD1B$^{-/-}$ HSPCs (Figure 6A). Altogether, knockout of PRMT5 or JMJD1B altered gene expression of 224 genes in opposite direction, in contrast to 15 genes in the same direction. We conducted pathway analysis on the 1,631 genes with altered gene expression in PRMT5$^{-/-}$ HSPCs and on 1,385 genes with altered gene expression in JMJD1B$^{-/-}$ HSPCs. We found that JMJD1B$^{-/-}$ HSPCs showed inhibition in 39 canonical pathways, 34 of which were activated in PRMT5$^{-/-}$ HSPCs (Figure 6B). At the same time, JMJD1B knockout resulted in inhibition of only one canonical pathway (PPAR), while PRMT5 knockout led to its activation (Figure 6B). This finding clearly supports our assertion that JMJD1B and PRMT5 function in an opposite way in HSPCs. Finally, we showed the predicted transcription factor signaling ($p < 0.05$) and gene expression levels of corresponding affected genes, whose H4R3me2s density was

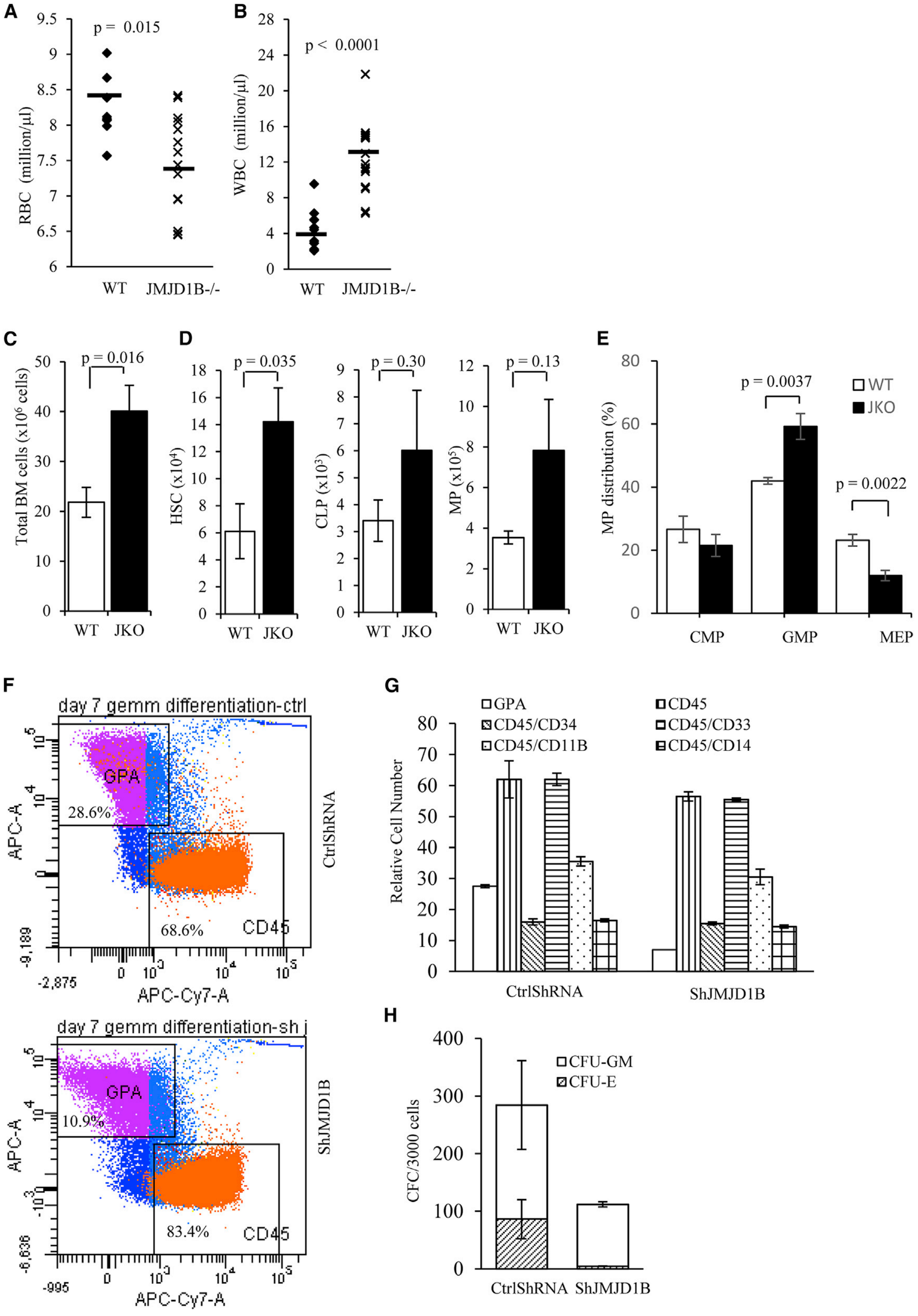

(legend on next page)

considerably increased in JMJD1B$^{-/-}$ versus WT HSPCs (Figure 6C). The most outstanding deduced transcription factors included p53 and CTNNB1, which are closely relevant to HSPC development.

JMJD1B Knockout Causes Defective Hematopoiesis

To test if defects in these pathways cause defective hematopoietic phenotypes in JMJD1B$^{-/-}$ mice, we first conducted a complete blood cell count (CBC) analysis of peripheral blood. JMJD1B$^{-/-}$ mice (~50%) developed anemia and showed a statistically significant decrease ($p = 0.015$) in total red blood cells compared with WT littermates (Figure 7A). The hemoglobin of JMJD1B$^{-/-}$ mice was 12.3 ± 0.2 g/dL, compared with 13.2 ± 0.2 g/dL in WT mice ($p = 0.013$). On the other hand, JMJD1B$^{-/-}$ mice (~80%) showed a 2.7-fold increase in white blood cells in CBC analysis ($p < 0.0001$; Figure 7B). The cell numbers of neutrophils and lymphocytes were 4.00 ± 0.47 and 7.80 ± 0.84 k/μL, respectively, in JMJD1B$^{-/-}$ mice, compared with 0.92 ± 0.15 and 3.44 ± 0.58 k/μL in WT mice ($p < 0.001$ for both cases). JMJD1B$^{-/-}$ mice also displayed a distinct blood cell lineage distribution, with an increase of neutrophils, from the WT (Figure S5A). These results were further confirmed by flow cytometry analysis (Figures S5B and S5C). In addition, JMJD1B$^{-/-}$ mice showed a decrease in the percentage of B lymphocytes and an increase in the T lymphocytes (Figures S5D and S5E).

We next conducted analysis of steady-state hematopoiesis on the bone marrow to identify the origin of the biased lineage. Consistent with downregulation of genes for cell-cycle arrest and cell death, the total numbers of BMCs and HSCs significantly increased in JMJD1B$^{-/-}$ mice compared with the WT (Figures 7C and 7D). The percentage of HSC but not common lymphoid progenitor (CLP) or myeloid progenitor (MP) population in JMJD1B$^{-/-}$ bone marrow increased, compared with the WT (Figure S6A). Although the number or percentage of MP cells had no statistically significant differences (Figures 7D and S6A), the distribution of MP in JMJD1B$^{-/-}$ bone marrow was distinct from that of the WT (Figure 7E). The megakaryocyte-erythrocyte progenitor (MEP) population was significantly reduced in JMJD1B$^{-/-}$ mice compared with WT littermates ($p = 0.0022$; Figure 7E). Also, the granulocyte-monocyte progenitor (GMP) population in JMJD1B$^{-/-}$ mice was significantly increased ($p = 0.0037$; Figures 7E and S6B). MEP and GMP cells differentiate into erythrocytes and neutrophils, respectively. Consistent with this, we observed relatively higher granulocyte in JMJD1B$^{-/-}$ bone marrow than in the WT (Figure S6C). These findings were consistent with the observation of less red blood cells and more white blood cells in JMJD1B$^{-/-}$ peripheral blood than in WT (Figures 7A and 7B).We further determined if JMJD1B similarly regulates human CD34^{+} HSPC differentiation. Knockdown of JMJD1B expression in human HSPCs reduced the proportion of glycophorin A (GPA)-expressing erythroid cells and thus increased the proportion of CD45^{+} leukocytes and CD33^{+} myeloid cells (Figures 7F and 7G). Inhibition of JMJD1B also completely constrained BFU-E/CFU-E (erythroid colony) formation from HSPC cells (Figure 7H). These results suggest that JMJD1B regulates the growth or differentiation of human primitive hematopoietic cells.

DISCUSSION

Our present study reveals that arginine demethylation of H4R3me2s is an active cellular process in HSPCs during hematopoiesis. The abundance of H4R3m2s as well as H3K9me2 markers inversely correlates with gene expression in HSPCs. This is consistent with previous findings, which implied that both H4R3me2s and H3K9me2 markers are repressive epigenetic markers for gene expression, possibly via modulation of the DNA methylation status and recruitment of transcription regulators (Chen et al., 2012; Girardot et al., 2014; Kim et al., 2012; Schones et al., 2014; Zhao et al., 2009). The evidence from our present study demonstrates the existence of arginine demethylases *in vivo* and a role for JMJD1B in demethylation of H4R3me2s to induce gene expression for hematopoiesis. Deficiency in JMJD1B disrupts the dynamics of H4R3me2s and H3K9me2 at the promoter regions of distinct groups of the genes that are important for development and differentiation of HSPCs, altering their expression. Consequently, JMJD1B$^{-/-}$ mice displayed abnormal phenotypes in the hematopoietic system, which are correlated with myelodysplastic syndrome (MDS), including leukocytosis, mild anemia, and granulocytosis. The JMJD1B gene is located at a locus (chromosome 5q arm) commonly deleted in a subcategory of MDS patients (5q syndrome) (Beurlet et al., 2013). Our results suggest that JMJD1B might be a tumor suppressor gene and contribute to MDS through regulation of the H4R3me2s marker to regulate gene

Figure 7. JMJD1B Deficiency Results in Abnormal Hematopoietic Development

(A–E) Abnormal hematopoiesis in JMJD1B knockout mice.

(A and B) Red blood cell (RBC) and white blood cell (WBC) count analysis of peripheral blood of WT ($n = 10$) and JMJD1B$^{-/-}$ ($n = 14$) mice.

(C) Total bone marrow (BM) cells from WT and JMJD1B$^{-/-}$ (JMJD1B knockout) mice.

(D) Total number of HSC (hematopoietic stem cell), CLP (common lymphoid progenitor), and MP (myeloid progenitor) cells in the BM of WT and JMJD1B$^{-/-}$ mice. They were calculated by multiplying the total bone marrow cell number in WT and JMJ1B$^{-/-}$ by the percentage of HSC, CLP, and MP (Figure S6A).

(E) Percentage distribution of different types of MPs: CMP (common myeloid progenitor), GMP (granulocyte-monocyte progenitor), and MEP (megakaryocyte-erythrocyte progenitor) cells relative to the MP population were analyzed by flow cytometry (Figure S6B).

In (C)–(E), values are mean ± SEM of five replicates. All p values were calculated using Student's t test (two-tailed).

(F–H). Knockdown of JMJD1B affects differentiation of human hematopoietic stem/progenitor cells (HSPCs). Cord blood CD34^{+} cells transduced with JMJD1B shRNA and control vector ($n = 2$) were selected by flow cytometry and cultured in GEMM for 7 days. Then the percentages of GPA-, CD45-, CD34-, CD11b-, CD33-, and CD14-positive cells were analyzed using flow cytometry. Representative results of flow cytometry analysis of GPA and CD45 expression are shown in (F). The total numbers of cells that expressed these markers at day 7 (G) are shown. The number of CFU-GM and BFU-E/CFU-E colonies generated from selected CD34^{+} GFP^{+} cells after culture in methylcellulose are shown in (H).

See also Figures S3, S6, and S7.

expression. One likely mechanism is that JMJD1B is required for maintenance of the H4R3me2s demethylation status for the induction of p53-mediated cell cycle checkpoint and cell death genes. This induction would allow HSPC cell cycle progression and proliferation to continue in a properly controlled manner. Supporting this hypothesis, we show that p53 signaling and corresponding affected genes that were of high H4R3me2s density in JMJD1B$^{-/-}$ HSPCs and were overlapped with genes whose expression was oppositely regulated by JMJD1B and PRMT5. On the other hand, we consider that JMJD1B-mediated H4R3me2s demethylation is only one of the mechanisms, by which JMJD1B regulates hematopoietic gene expression. We observed that many genes, which are regulated by both JMJD1B and PRMT5, displayed little change in H4R3me2s density in the WT and JMJD1B$^{-/-}$ HSPCs. It suggests that JMJD1B and PRMT5 may also cooperatively regulate the dynamics of arginine methylation status of non-histone proteins such as transcription regulators to control gene expression for hematopoiesis and MDS avoidance.

We have demonstrated that JMJD1B demethylates both H3K9me2 and H4R3me2s substrates with similar efficiency *in vitro*. However, JMJD1B distinctly mediates H3K9me2 and H4R3me2s demethylation at different loci *in vivo*. It may simultaneously demethylate H3K9me2 and H4R3me2s in groups of genes, including the target genes of NF-kB. It may primarily demethylate H3K9me2 but not H4R3me2s at certain groups of genes, including the target genes of STAT3, SMAD3, and WT1, and it may only demethylate H4R3me2 but not H3K9me2s in the groups of genes such as those regulated by transcription factors CTNNB1, ERG, and SOX2. We hypothesize that during hematopoiesis and other development processes, other factors including nearby histone modification status, JMJD1B modifications, proteins bound to chromosome and/or JMJD1B may determine whether JMJD1B demethylate H3K9me2, H4R3me2s, or both.

An interesting question is whether inhibition of PRMT5 may rescue the phenotype because of JMJD1B deletion. PRMT5 and JMJD1B possess the reversal enzyme activities, and we revealed that knockout of PRMT5 resulted in opposed molecular changes to JMJD1B knockout. For instance, PRMT5 deletion resulted in p21 (Cdkn 1a) upregulation in HSPCs (Liu et al., 2015a), but JMJD1B knockout led to p21 downregulation in HSPCs. Consequently, PRMT5 knockout mice had a decrease in the number of HSPCs and white blood cells (Liu et al., 2015a), but JMJD1B$^{-/-}$ knockout mice had an increase in the number of HSPCs and white blood cells. Likewise, JMJD1B deficiency may promote cancer cell proliferation and survival via inhibition of p53-p21 signaling. Such alterations might be corrected by inhibition of PRMT5 to induce p21 expression. However, inhibition of PRMT5 may not completely rescue the phenotypes of the JMJD1B$^{-/-}$ mice because the arginine demethylation is not simple reversal of methylation in cells. We suggest that JMJD1B works with PRMT5 to program the dynamic histone arginine methylation balance for turning hematopoietic development genes on/off in a sequential manner. Deficiency in either PRMT5 or JMJD1B would disrupt such an epigenetic program and lead to abnormal development of germ cells (Liu et al., 2015b, 2015c).

JmjC domain-containing proteins are the largest class of potential histone demethylases, and the JmjC domains of several proteins have been shown to demethylate mono-, di-, and tri-methyl lysine residues via an oxidative reaction that requires iron and α-ketoglutarate (Cloos et al., 2008; Klose et al., 2006; Klose and Zhang, 2007). However, the enzyme activities and specificities of most JmjC domain-containing proteins have not been experimentally tested (Greer and Shi, 2012), and it is possible that additional JmjC domain-containing arginine demethylases exist and are important in demethylating methylarginine in different histones or non-histone proteins to regulate different cellular processes. Indeed, JMJD1B knockout only increased the H4R3me2s signal in a group of genes depleted of H4R3me2s. It is consistent with our suggestion that additional arginine demethylases are important in maintaining the demethylation status of H4R3me2s and other histone arginine methylation markers. Our present study describes an important function for JmjC domain-containing enzymes in regulating arginine methylation and demonstrates the need to more clearly define the biochemical properties and *in vivo* functions of other JmjC domain containing enzymes.

EXPERIMENTAL PROCEDURES

Further details and an outline of resources used in this work can be found in Supplemental Experimental Procedures.

Animal Studies

Twenty to 40 weeks of male and female mice were used in this study. All protocols that involved animals were approved by the Institutional Animal Care and Use Committee of City of Hope in compliance with the Public Health Service Policy of the United States and all other federal, state, and local regulations. All aspects of the animal study were adequately reported following the NIH guideline.

Quantification and Statistical Analyses

The band intensity of western blot analysis was quantified using ImageJ software (NIH). All data are expressed as mean ± SEM, and significance (p value) was calculated using two-tailed Student's t test for between-group differences; p values < 0.05 were considered to indicate statistical significance.

DATA AND SOFTWARE AVAILABILITY

The accession number for the genomic data reported in this paper is GEO: GSE94966. Raw image data were deposited in Mendeley (https://doi.org/10.17632/8f8f53ph8m.1).

SUPPLEMENTAL INFORMATION

Supplemental Information includes Supplemental Experimental Procedures, six figures, and three tables and can be found with this article online at https://doi.org/10.1016/j.celrep.2018.03.051.

ACKNOWLEDGMENTS

The Core Facilities at City of Hope are supported by the National Cancer Institute of the NIH under award number P30CA033572. This work was supported by NIH grants R01CA073764 to B.S. and R50CA211397 to L. Zheng. The content is solely the responsibility of the authors and does not necessarily represent the official views of the NIH. This work was also supported by the National Natural Science Foundation of China (grant 31401085) and grant 2014M560477 from the China Postdoctoral Science Foundation to S. Li. We thank Nancy J. Linford, PhD, and Sarah T. Wilkinson, PhD, for editorial assistance.

AUTHOR CONTRIBUTIONS

S. Li, L. Zheng, S.A., and X.D. designed and conducted *in vitro* and *in vivo* experiments and contributed to manuscript preparation. S. Liu, C.L., L. Zhou, Z.G., H.X., Y.H., and Y.Y. conducted biochemical assays. S.A., H.D., L.L., P.C., and R.B. conducted analysis on hematopoietic development and other mouse phenotypes. L. Zheng, S.A., J.D., L.Y., M.Z., D.E.S., and X.W. conducted RNA-seq, ChIP-seq, and data analysis. L. Zheng, Z.G., and Y.Y. also contributed to manuscript preparation. B.S. supervised the entire project, designed and coordinated most of the experiments, and coordinated manuscript preparation.

DECLARATION OF INTERESTS

The authors declare no competing interests.

Received: June 23, 2017
Revised: January 23, 2018
Accepted: March 13, 2018
Published: April 10, 2018

REFERENCES

Bedford, M.T., and Clarke, S.G. (2009). Protein arginine methylation in mammals: who, what, and why. Mol. Cell *33*, 1–13.

Beurlet, S., Chomienne, C., and Padua, R.A. (2013). Engineering mouse models with myelodysplastic syndrome human candidate genes; how relevant are they? Haematologica *98*, 10–22.

Blanc, R.S., and Richard, S. (2017). Arginine methylation: the coming of age. Mol. Cell *65*, 8–24.

Chang, B., Chen, Y., Zhao, Y., and Bruick, R.K. (2007). JMJD6 is a histone arginine demethylase. Science *318*, 444–447.

Chen, C., Nott, T.J., Jin, J., and Pawson, T. (2011). Deciphering arginine methylation: Tudor tells the tale. Nat. Rev. Mol. Cell Biol. *12*, 629–642.

Chen, X., Skutt-Kakaria, K., Davison, J., Ou, Y.L., Choi, E., Malik, P., Loeb, K., Wood, B., Georges, G., Torok-Storb, B., and Paddison, P.J. (2012). G9a/GLP-dependent histone H3K9me2 patterning during human hematopoietic stem cell lineage commitment. Genes Dev. *26*, 2499–2511.

Chung, Y.J., Park, B.B., Kang, Y.J., Kim, T.M., Eaves, C.J., and Oh, I.H. (2006). Unique effects of Stat3 on the early phase of hematopoietic stem cell regeneration. Blood *108*, 1208–1215.

Cloos, P.A., Christensen, J., Agger, K., and Helin, K. (2008). Erasing the methyl mark: histone demethylases at the center of cellular differentiation and disease. Genes Dev. *22*, 1115–1140.

Cunningham, T.J., Palumbo, I., Grosso, M., Slater, N., and Miles, C.G. (2013). WT1 regulates murine hematopoiesis via maintenance of VEGF isoform ratio. Blood *122*, 188–192.

Fuhrmann, J., Clancy, K.W., and Thompson, P.R. (2015). Chemical biology of protein arginine modifications in epigenetic regulation. Chem. Rev. *115*, 5413–5461.

Girardot, M., Hirasawa, R., Kacem, S., Fritsch, L., Pontis, J., Kota, S.K., Filipponi, D., Fabbrizio, E., Sardet, C., Lohmann, F., et al. (2014). PRMT5-mediated histone H4 arginine-3 symmetrical dimethylation marks chromatin at G + C-rich regions of the mouse genome. Nucleic Acids Res. *42*, 235–248.

Greer, E.L., and Shi, Y. (2012). Histone methylation: a dynamic mark in health, disease and inheritance. Nat. Rev. Genet. *13*, 343–357.

Guo, Z., Zheng, L., Xu, H., Dai, H., Zhou, M., Pascua, M.R., Chen, Q.M., and Shen, B. (2010). Methylation of FEN1 suppresses nearby phosphorylation and facilitates PCNA binding. Nat. Chem. Biol. *6*, 766–773.

Huang, H., Sabari, B.R., Garcia, B.A., Allis, C.D., and Zhao, Y. (2014). SnapShot: histone modifications. Cell *159*, 458–458.e1.

Ito, K., Carracedo, A., Weiss, D., Arai, F., Ala, U., Avigan, D.E., Schafer, Z.T., Evans, R.M., Suda, T., Lee, C.H., and Pandolfi, P.P. (2012). A PML–PPAR-δ pathway for fatty acid oxidation regulates hematopoietic stem cell maintenance. Nat. Med. *18*, 1350–1358.

Jansson, M., Durant, S.T., Cho, E.C., Sheahan, S., Edelmann, M., Kessler, B., and La Thangue, N.B. (2008). Arginine methylation regulates the p53 response. Nat. Cell Biol. *10*, 1431–1439.

Kim, J.Y., Kim, K.B., Eom, G.H., Choe, N., Kee, H.J., Son, H.J., Oh, S.T., Kim, D.W., Pak, J.H., Baek, H.J., et al. (2012). KDM3B is the H3K9 demethylase involved in transcriptional activation of lmo2 in leukemia. Mol. Cell. Biol. *32*, 2917–2933.

Klose, R.J., and Zhang, Y. (2007). Regulation of histone methylation by demethylimination and demethylation. Nat. Rev. Mol. Cell Biol. *8*, 307–318.

Klose, R.J., Kallin, E.M., and Zhang, Y. (2006). JmjC-domain-containing proteins and histone demethylation. Nat. Rev. Genet. *7*, 715–727.

Ku, C.J., Hosoya, T., Maillard, I., and Engel, J.D. (2012). GATA-3 regulates hematopoietic stem cell maintenance and cell-cycle entry. Blood *119*, 2242–2251.

Larsson, J., and Karlsson, S. (2005). The role of Smad signaling in hematopoiesis. Oncogene *24*, 5676–5692.

Le Romancer, M., Treilleux, I., Leconte, N., Robin-Lespinasse, Y., Sentis, S., Bouchekioua-Bouzaghou, K., Goddard, S., Gobert-Gosse, S., and Corbo, L. (2008). Regulation of estrogen rapid signaling through arginine methylation by PRMT1. Mol. Cell *31*, 212–221.

Lee, K.H., Ju, U.I., Song, J.Y., and Chun, Y.S. (2014). The histone demethylase PHF2 promotes fat cell differentiation as an epigenetic activator of both C/EBPα and C/EBPδ. Mol. Cells *37*, 734–741.

Liebermann, D.A., Gregory, B., and Hoffman, B. (1998). AP-1 (Fos/Jun) transcription factors in hematopoietic differentiation and apoptosis. Int. J. Oncol. *12*, 685–700.

Liu, Y., Elf, S.E., Miyata, Y., Sashida, G., Liu, Y., Huang, G., Di Giandomenico, S., Lee, J.M., Deblasio, A., Menendez, S., et al. (2009). p53 regulates hematopoietic stem cell quiescence. Cell Stem Cell *4*, 37–48.

Liu, F., Cheng, G., Hamard, P.J., Greenblatt, S., Wang, L., Man, N., Perna, F., Xu, H., Tadi, M., Luciani, L., and Nimer, S.D. (2015a). Arginine methyltransferase PRMT5 is essential for sustaining normal adult hematopoiesis. J. Clin. Invest. *125*, 3532–3544.

Liu, Z., Chen, X., Zhou, S., Liao, L., Jiang, R., and Xu, J. (2015b). The histone H3K9 demethylase Kdm3b is required for somatic growth and female reproductive function. Int. J. Biol. Sci. *11*, 494–507.

Liu, Z., Oyola, M.G., Zhou, S., Chen, X., Liao, L., Tien, J.C., Mani, S.K., and Xu, J. (2015c). Knockout of the histone demethylase Kdm3b decreases spermatogenesis and impairs male sexual behaviors. Int. J. Biol. Sci. *11*, 1447–1457.

Mantri, M., Krojer, T., Bagg, E.A., Webby, C.J., Butler, D.S., Kochan, G., Kavanagh, K.L., Oppermann, U., McDonough, M.A., and Schofield, C.J. (2010). Crystal structure of the 2-oxoglutarate- and Fe(II)-dependent lysyl hydroxylase JMJD6. J. Mol. Biol. *401*, 211–222.

Mikhaleva, I.I., Prudchenko, I.A., Ivanov, V.T., and Voitenkov, V.B. (2011). JmjC-domain-containing histone demethylases of the JMJD1B type as putative precursors of endogenous DSIP. Peptides *32*, 826–831.

Ng, A.P., Loughran, S.J., Metcalf, D., Hyland, C.D., de Graaf, C.A., Hu, Y., Smyth, G.K., Hilton, D.J., Kile, B.T., and Alexander, W.S. (2011). Erg is required for self-renewal of hematopoietic stem cells during stress hematopoiesis in mice. Blood *118*, 2454–2461.

Qi, H.H., Sarkissian, M., Hu, G.Q., Wang, Z., Bhattacharjee, A., Gordon, D.B., Gonzales, M., Lan, F., Ongusaha, P.P., Huarte, M., et al. (2010). Histone H4K20/H3K9 demethylase PHF8 regulates zebrafish brain and craniofacial development. Nature *466*, 503–507.

Sarkar, A., and Hochedlinger, K. (2013). The sox family of transcription factors: versatile regulators of stem and progenitor cell fate. Cell Stem Cell *12*, 15–30.

Scheller, M., Huelsken, J., Rosenbauer, F., Taketo, M.M., Birchmeier, W., Tenen, D.G., and Leutz, A. (2006). Hematopoietic stem cell and multilineage defects generated by constitutive beta-catenin activation. Nat. Immunol. *7*, 1037–1047.

Schones, D.E., Chen, X., Trac, C., Setten, R., and Paddison, P.J. (2014). G9a/GLP-dependent H3K9me2 patterning alters chromatin structure at CpG islands in hematopoietic progenitors. Epigenetics Chromatin *7*, 23.

Shi, Y., Lan, F., Matson, C., Mulligan, P., Whetstine, J.R., Cole, P.A., Casero, R.A., and Shi, Y. (2004). Histone demethylation mediated by the nuclear amine oxidase homolog LSD1. Cell *119*, 941–953.

Sims, R.J., 3rd, Rojas, L.A., Beck, D.B., Bonasio, R., Schüller, R., Drury, W.J., 3rd, Eick, D., and Reinberg, D. (2011). The C-terminal domain of RNA polymerase II is modified by site-specific methylation. Science *332*, 99–103.

Stier, S., Cheng, T., Dombkowski, D., Carlesso, N., and Scadden, D.T. (2002). Notch1 activation increases hematopoietic stem cell self-renewal in vivo and favors lymphoid over myeloid lineage outcome. Blood *99*, 2369–2378.

Tothova, Z., Kollipara, R., Huntly, B.J., Lee, B.H., Castrillon, D.H., Cullen, D.E., McDowell, E.P., Lazo-Kallanian, S., Williams, I.R., Sears, C., et al. (2007). FoxOs are critical mediators of hematopoietic stem cell resistance to physiologic oxidative stress. Cell *128*, 325–339.

Walport, L.J., Hopkinson, R.J., Chowdhury, R., Schiller, R., Ge, W., Kawamura, A., and Schofield, C.J. (2016). Arginine demethylation is catalysed by a subset of JmjC histone lysine demethylases. Nat. Commun. *7*, 11974.

Webby, C.J., Wolf, A., Gromak, N., Dreger, M., Kramer, H., Kessler, B., Nielsen, M.L., Schmitz, C., Butler, D.S., Yates, J.R., 3rd., et al. (2009). Jmjd6 catalyses lysyl-hydroxylation of U2AF65, a protein associated with RNA splicing. Science *325*, 90–93.

Yang, Y., and Bedford, M.T. (2013). Protein arginine methyltransferases and cancer. Nat. Rev. Cancer *13*, 37–50.

Yu, Z., Chen, T., Hébert, J., Li, E., and Richard, S. (2009). A mouse PRMT1 null allele defines an essential role for arginine methylation in genome maintenance and cell proliferation. Mol. Cell. Biol. *29*, 2982–2996.

Zhao, Q., Rank, G., Tan, Y.T., Li, H., Moritz, R.L., Simpson, R.J., Cerruti, L., Curtis, D.J., Patel, D.J., Allis, C.D., et al. (2009). PRMT5-mediated methylation of histone H4R3 recruits DNMT3A, coupling histone and DNA methylation in gene silencing. Nat. Struct. Mol. Biol. *16*, 304–311.

Cell Reports, Volume *23*

Supplemental Information

JMJD1B Demethylates H4R3me2s and H3K9me2 to Facilitate Gene Expression for Development of Hematopoietic Stem and Progenitor Cells

Sihui Li, Shafat Ali, Xiaotao Duan, Songbai Liu, Juan Du, Changwei Liu, Huifang Dai, Mian Zhou, Lina Zhou, Lu Yang, Peiguo Chu, Ling Li, Ravi Bhatia, Dustin E. Schones, Xiwei Wu, Hong Xu, Yuejin Hua, Zhigang Guo, Yanzhong Yang, Li Zheng, and Binghui Shen

Supplemental Table S1: Methylated histone tail peptide substrates* and antibodies used in western blotting analysis. Related to Figure 1 and Figure 3

Substrates	Sequences	Peptide Resources	Antibody Resources
H3K9me2	ARTKQTAR K(Me2) - STGGKAPRKQLAGGK(Biotin)	AnaSpec 64627-025	Abcam Ab1220
H3K4me2	ARTK(Me2)QTARKSTGGKAPRKQLA-GGK(Biotin)	AnaSpec 64356-025	Active Motif 39679
H3R2me2s	AR(Me2s)TKQTARKSTGGKAPRKQLA-GGK(Biotin)	AnaSpec 64631-025	Active Motif 39703
H3R2me2a	AR(Me2a)TKQTARKSTGGKAPRKQLA-GGK(Biotin)	AnaSpec 64630-025	Abcam Ab80075
H3R2me1	AR(Me1)TKQTARKSTGGKAPRKQLAGG K(Biotin)	AnaSpec 64629-025	Abcam ab15584
H3R8me2s	ARTKQTAR(Me2s)KSTGGKAPRKQLA GGK(Biotin)	AnaSpec 65351-025	Said Sif Ohio State Univ.
H3R8me1	ARTKQTAR(Me1)KSTGGKAPRKQ-LAK(Biotin)	AnaSpec 64607-025	Active Motif 39673
H3R17me2a	ARTKQTARKSTGGKAPR(Me2a)-KQLAGGK(Biotin	AnaSpec 64634-025	Active Motif 39709
H3R26me2a	APRKQLATKAAR(Me2a)KSAPATGGVK-GGK(Biotin)	AnaSpec 65261-025	EMD Millipore 07-215
H4R3me2s	SGR(Me2s)GKGGKGLGKGGAKRHRKV-GGK(Biotin)	AnaSpec 65424-025	Active Motif 61187
H4R3me2a	SGR(Me2a)GKGGKGLGKGGAKRHRKV-GGK(Biotin)	AnaSpec 64976-025	Active Motif 39706
H4R3me1	SGR(Me1)GKGGKGLGKGGAKRHRKV-GGK(Biotin)	AnaSpec 64977-025	Abcam Ab17339

*Custom peptide synthesis

Supplemental Table S2: Peptides* for arginine demethylation assay by mass spectrometry. Related to Figure 2

Peptides	Sequences	Resources
H3K9me2	ARTKQTAR K(Me2) STGGKAPRKQLATKAA	Anaspec 62622-5
H4R3me2s	SGR(Me2s)GKGGKGLGKGGAK	Anaspec 62392-1
H4R3me2a	SGR(Me2a)GKGGKGLGKGGAK	Anaspec 62622-3
H3R2me1	SGR(Me1)GKGGKGLGKGGAK	Anaspec 62392-2

*Custom peptide synthesis

Supplemental Table S3: Summary of ChIP-Seq and alignment. Related to Figure 4 and Figure 5.

Sample	Total Reads	Unique Aligned Reads	% Aligned	Non-redundant Reads
WT-HSPC-INPUT	58,792,986	49,010,628	83.4%	46,407,639
WT-HSPC-H4R3ME2S	117,604,443	92,976,262	79.1%	83,718,326
WT-HSPC-H3K9ME2	115,014,233	87,872,149	76.4%	81,224,010
WT-HSPC-H4	112,649,572	81,278,139	72.2%	71,254,082
WT-BMC-INPUT	62,493,414	51,494,573	82.4%	48,205,709
WT-BMC-H4R3ME2S	40,909,933	33,873,424	82.8%	27,724,518
WT-BMC-H3K9me2	50,164,784	38,526,554	77.0%	36,238,249
JKO-HSPC-INPUT	54,501,763	45,248,507	83.4%	42,962,961
JKO-HSPC-H4R3ME2S	102,856,353	83,608,487	81.3%	76,948,453
JKO-HSPC-H3K9ME2	101,544,449	83,127,518	81.9%	77,417,891
JKO-HSPC-H4	107,300,436	77,308,068	72.0%	67,765,370
JKO-BMC-INPUT	63,118,338	51,441,445	82.0%	48,083,779
JKO-BMC-H4R3ME2S	42,059,087	32,848,146	78.0%	30,027,216
JKO-BMC-H3K9ME2	53,731,355	43,200,009	80.0%	40,995,991

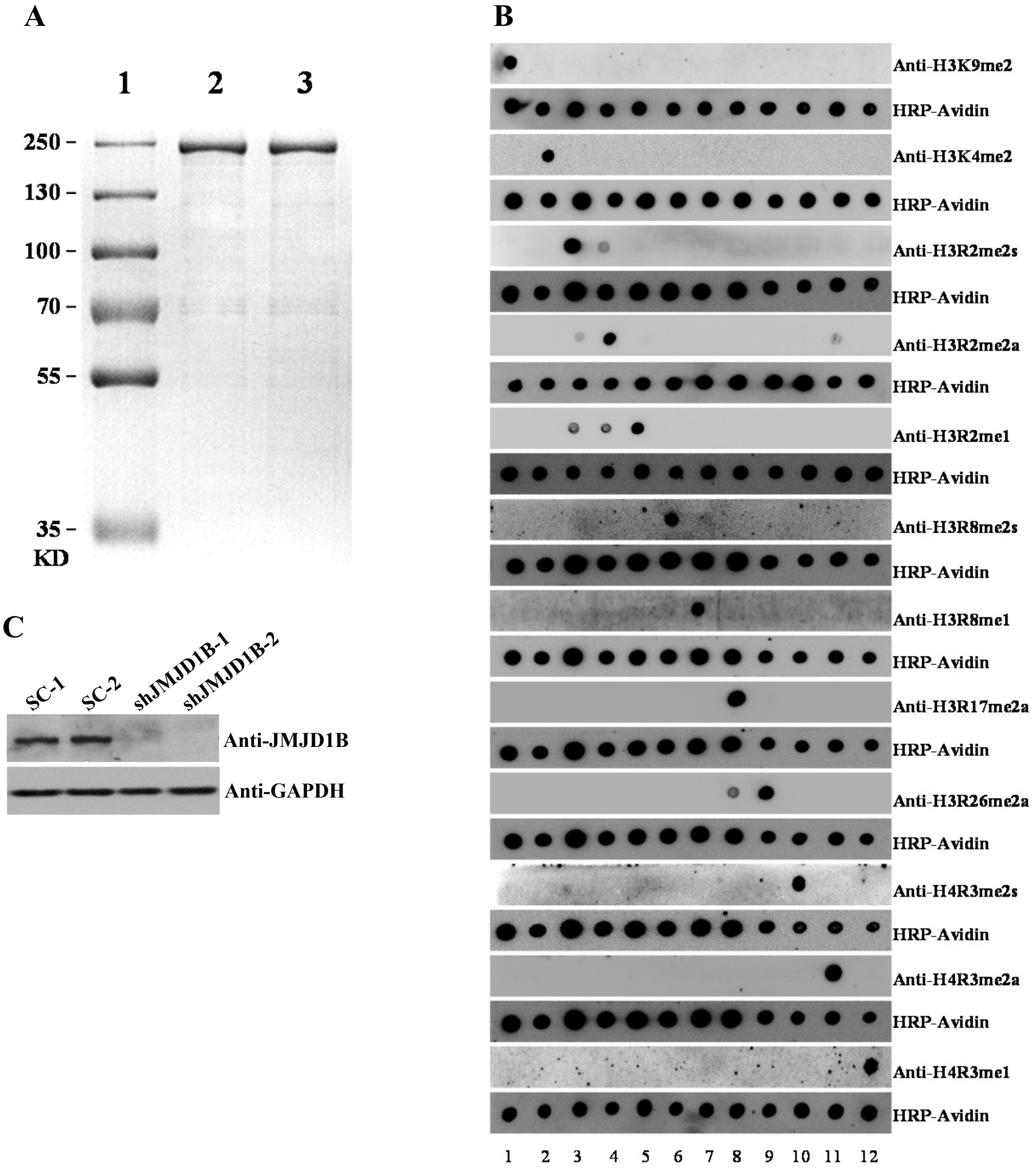

Supplemental Figure S1. Validation of purity of recombinant JMJD1B proteins, specificity of histone antibodies, and siRNA efficiency. Related to Figures 1-3. (A) JMJD1B expression and purification. PEZ-M12-JMJD1B (isoform 1) was purchased from GeneCopoeia (EX-E1505-M12) with a 3 x FLAG tag. JMJD1B protein After expression in HEK293T cells, the recombinant proteins were purified using M2 magnetic beads (Sigma). Shown is the Coomassie Brilliant Blue-stained gel demonstrating the purity of the WT (lane 2) and mutant (H1560A/D1562A/H1689A) (lane 3) JMJD1B proteins, along with molecular weight markers (Lane 1). **(B)** Dot blot analyses verify the specificity of histone antibodies. Equal amount (1 μg) of biotin-synthetic modified histone peptides were dotted on the nitrocellulose membrane at the following order from left to right: 1. H3K9me2; 2. H3K4me2; 3. H3R2me2s; 4. H3R2me2a; 5. H3R2me1; 6. H3R8me2s; 7. H3R8me1; 8. H3R17me2a; 9. H3R26me2a; 10. H4R3me2s; 11. H4R3me2a; 12. H4R3me1. Standard dot blot protocol was performed using indicated antibodies against specific forms of modified histones. For controls, membranes were stripped and reprobed with HRP-avidin. **(C)** Knockdown of JMJD1B by shRNA in 293T cells. 293T cells were transfected with scrambled shRNA vectors (SC-1: Cat No. SHC001 and SC-2: SHC002) or shRNA vectors against JMJD1B [shJMJD1B-1: Cat No. TRCN0000017093 and shJMJD1B-2: (Sigma, Cat No. TRCN0000017093). 48 h post-transfection, the JMJD1B level was analyzed with western blot using the anti-JMJD1B antibody. GAPDH was used as a loading control.

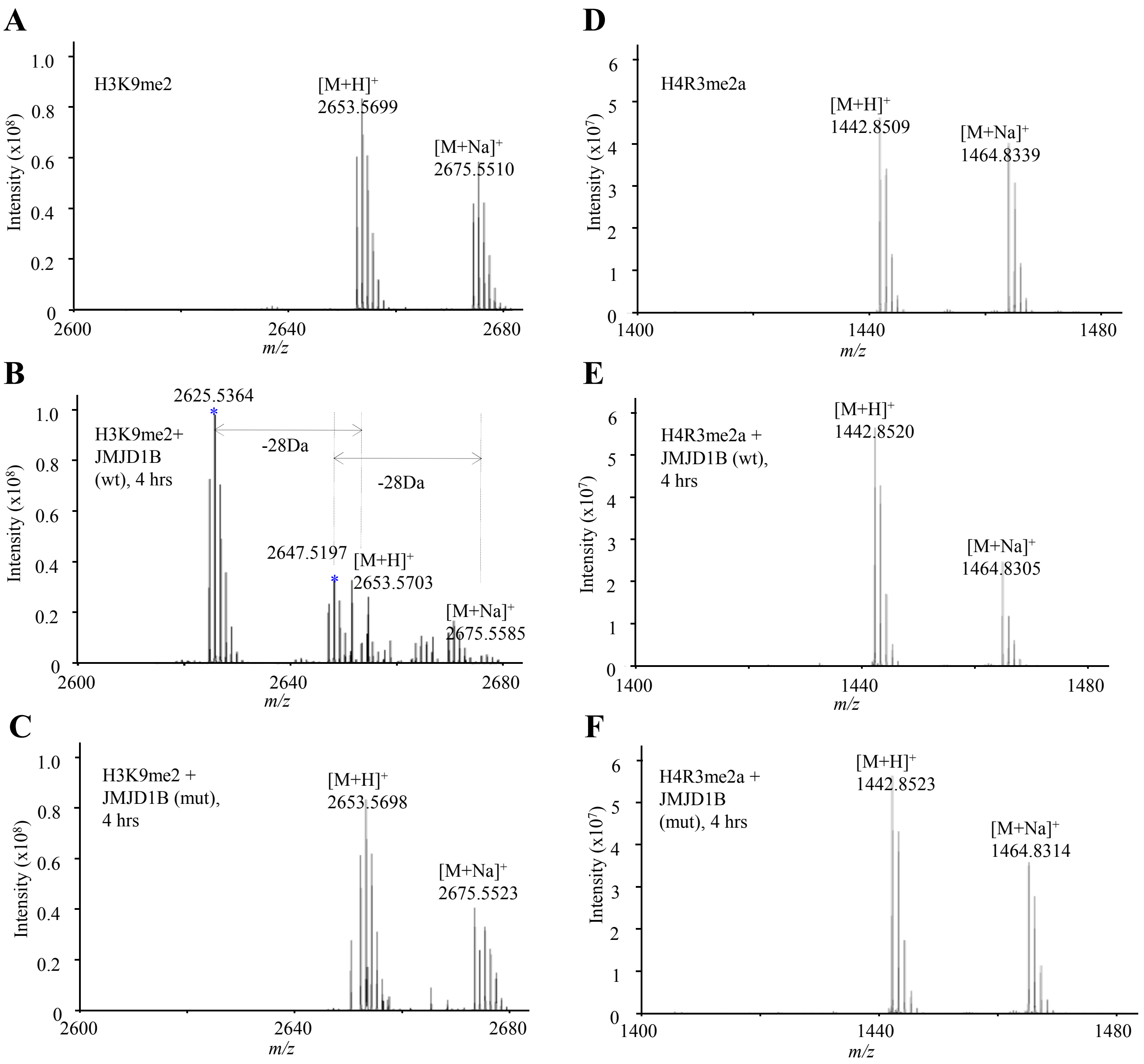

Supplemental Figure S2. JMJD1B demethylase activity assay on H3K9me2 and H4R3me2a by mass spectrometry. Related to Figure 2. (**A**)-(**C**) H3K9me2 is demethylated by JMJD1B. The H3K9me2 peptide substrate (18 μM) was incubated at 37ºC for 4 h in demethylation buffer alone (**A**), or with 0.5 μM wild-type JMJD1B (wt) (**B**) or 0.5 μM mutant JMJD1B (mut) (**C**). The reaction mixtures were analyzed by mass spectrometry. Representative mass spectrometry images are shown, and (*) indicates the demethylation products in the reactions containing the H3K9me2 peptide and WT JMJD1B (B). No product was found in the reaction with the mutant JMJD1B (**C**). (**D**)-(**F**) H4R3me2a is not demethylated by JMJD1B. The H4R3me2a peptide substrate (34 μM) was incubated at 37ºC for 4 h in demethylation buffer alone (**D**), or with 0.5 μM of JMJD1B (wt) (**E**) or 0.5 μM of JMJD1B (mut) (**F**). The reaction mixtures were analyzed by mass spectrometry. Representative mass spectrometry images are shown, demonstrating the absence of products in the reactions containing JMJD1B and the H4R3me2a peptide (**E**).

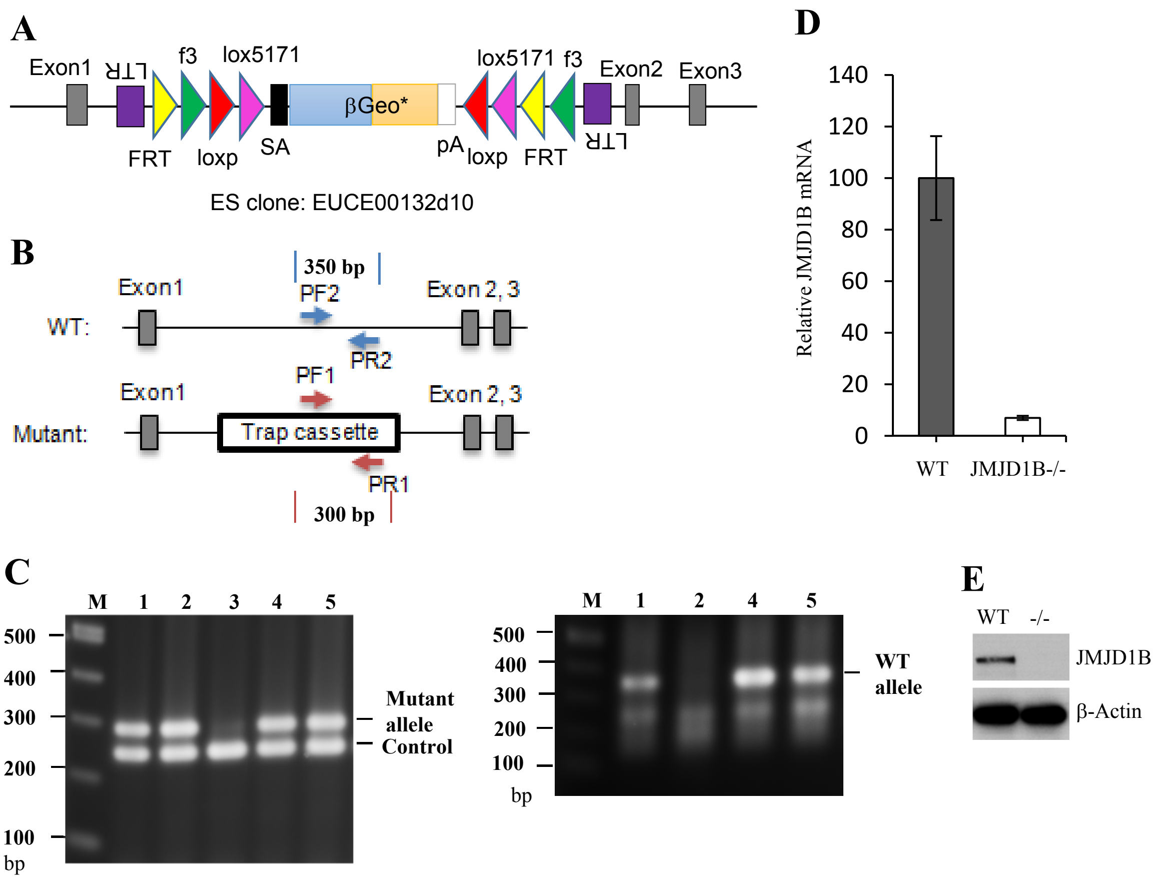

Supplemental Figure S3. Knockout of JMJD1B via gene trap technology. Related to Figures 5 and Figure 7. (**A**) Schematic map indicating insertion of the gene trap cassette (rFlipROSAbeta-Geo*) into intron 1 of JMJD1B. The gene cassette carrying a splicing acceptor (SA) at the 5' end and a polyA sequence at the 3' end causes premature termination of transcription and splicing, abolishing JMJD1B expression. (**B**) and (**C**) Screening of JMJD1B mutant mice by PCR. Panel b shows the strategy and primers for PCR amplification. The mutant allele was detected by PCR using the PF1/PR1 primers, which correspond to the DNA sequences of the trap cassette. The WT allele, at the trap cassette insertion site, was detected by PCR using the PF2/PR2 primers. Panel c shows representative images of agarose gel electrophoresis of PCR products amplified by PF1/PR1 (left) and PF2/PR2 (right). In the first PCR reaction (left), a pair of control primers corresponding to the actin gene was used as an internal control to ensure the quality of the PCR reactions. The first PCR reaction detected the mutant allele, and the second PCR reaction detected the WT allele. Mouse #3 (WT) carried no mutant allele; Mice #1, 4, and 5 (heterozygous) carried both the WT and mutant alleles; and mouse #2 (mutant) carried only the mutant allele. (**D**) mRNA levels of JMJD1B in WT and JMJD1B-/- MEF cells (E13.5). Total RNA was extracted from WT and JMJD1B-/- cells and real-time RT-PCR was used to quantify the levels of JMJD1B mRNA and control β-actin mRNA. The JMJD1B mRNA level was normalized to the corresponding β-actin mRNA level. The normalized JMJD1B mRNA level in the WT cells was arbitrarily set to 100. The values are the means ± s.d. of three independent assays. (**E**) Western blot to detect JMJD1B protein levels in WT and JMJD1B-/- embryos (E11.5). Whole cell extracts were prepared from WT and JMJD1B embryos and resolved by 4-20% SDS-PAGE. The JMJD1B protein was detected by western blotting using an anti-JMJD1B antibody (Bethyl).

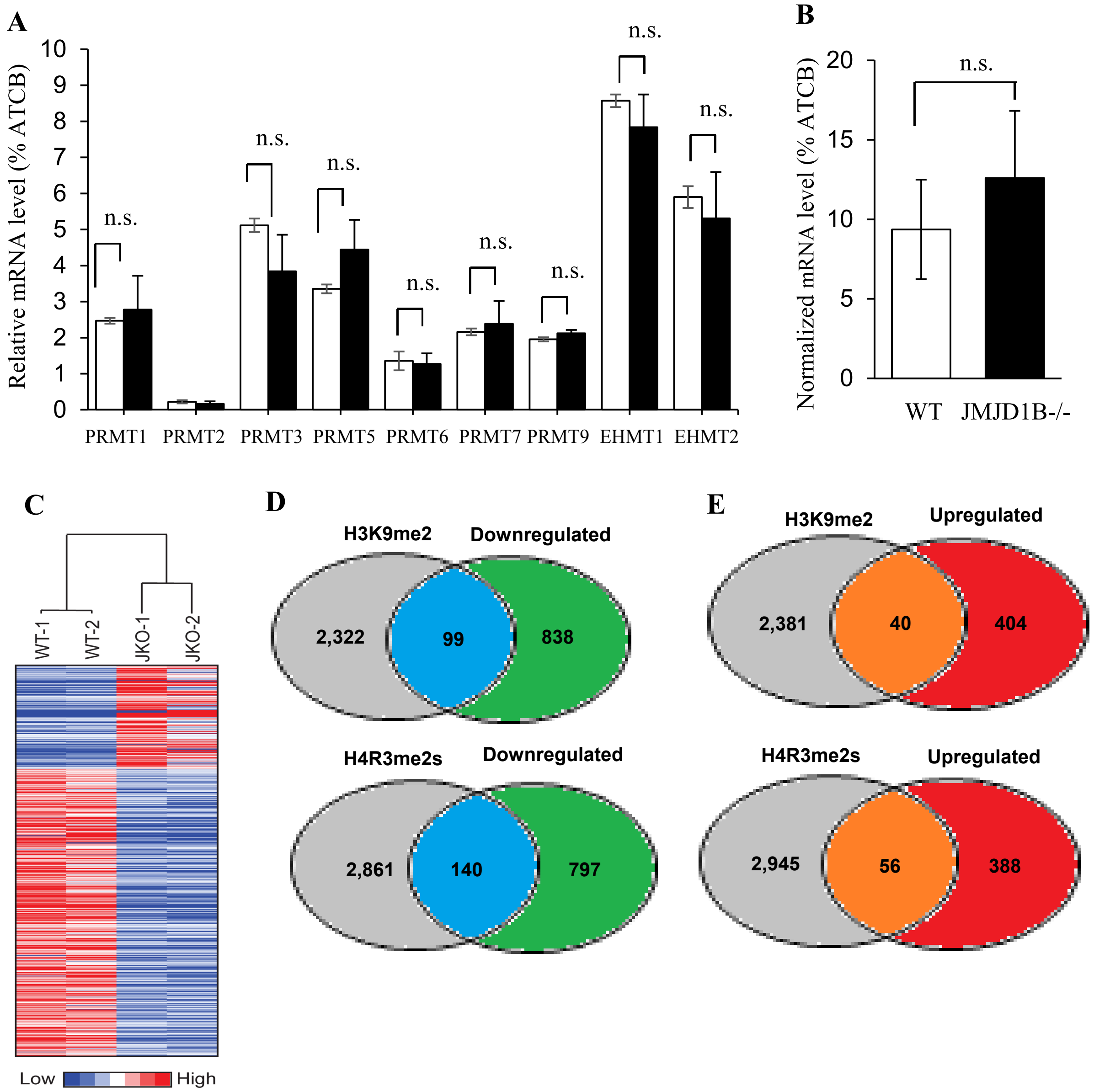

Supplemental Figure S4. Gene expression profiles in WT and JMJD1B-/- HSPCs. Related to Figure 5. (A) and (B) Relative expression of protein arginine and lysine methyltransferases in WT and JMJD1B-/- HSPCs. **(A)** The mRNA levels of PRMTs and EHMTs were determined by RNA-Seq. The relative mRNA level of PRMTs and EHMTs was normalized with corresponding mRNA level of β-actin, which is arbitrarily set as 100. Values are means ± s.d. for two biological replicates for each genetic background. (**B)** The mRNA levels of PRMT5 were determined by quantitative PCR. The relative mRNA level of PRMTs was normalized with corresponding mRNA level of β-actin, which was arbitrarily set as 100. Values are means ± s.d. for three biological replicates for each genetic background. p value: Student's t-test. **(C)** Heatmap of gene expression profilings in WT and JMJD1B$^{-/-}$ (JKO) HSPCs. **(D) and (E)** Intersection of the gene lists with increased H3K9me2 or H4R3me2s density and the gene lists of down- (Panel D) or up-regulated genes (Panel E) (JMJD1B$^{-/-}$ vs WT). Genes with FC of JMJD1B$^{-/-}$/WT in H4R3me2s or H3K9me2 signal no less than 1.5-fold and the JKO ChIP reads in H4R3me2s or H3K9me2 ≥ 15 were selected as increased H3K9me2 or H4R3me2s genes. Genes with FC of JMJD1B$^{-/-}$/WT in gene expression being less than or greater than 0 and $p < 0.05$ were selected as down- and up-regulated genes.

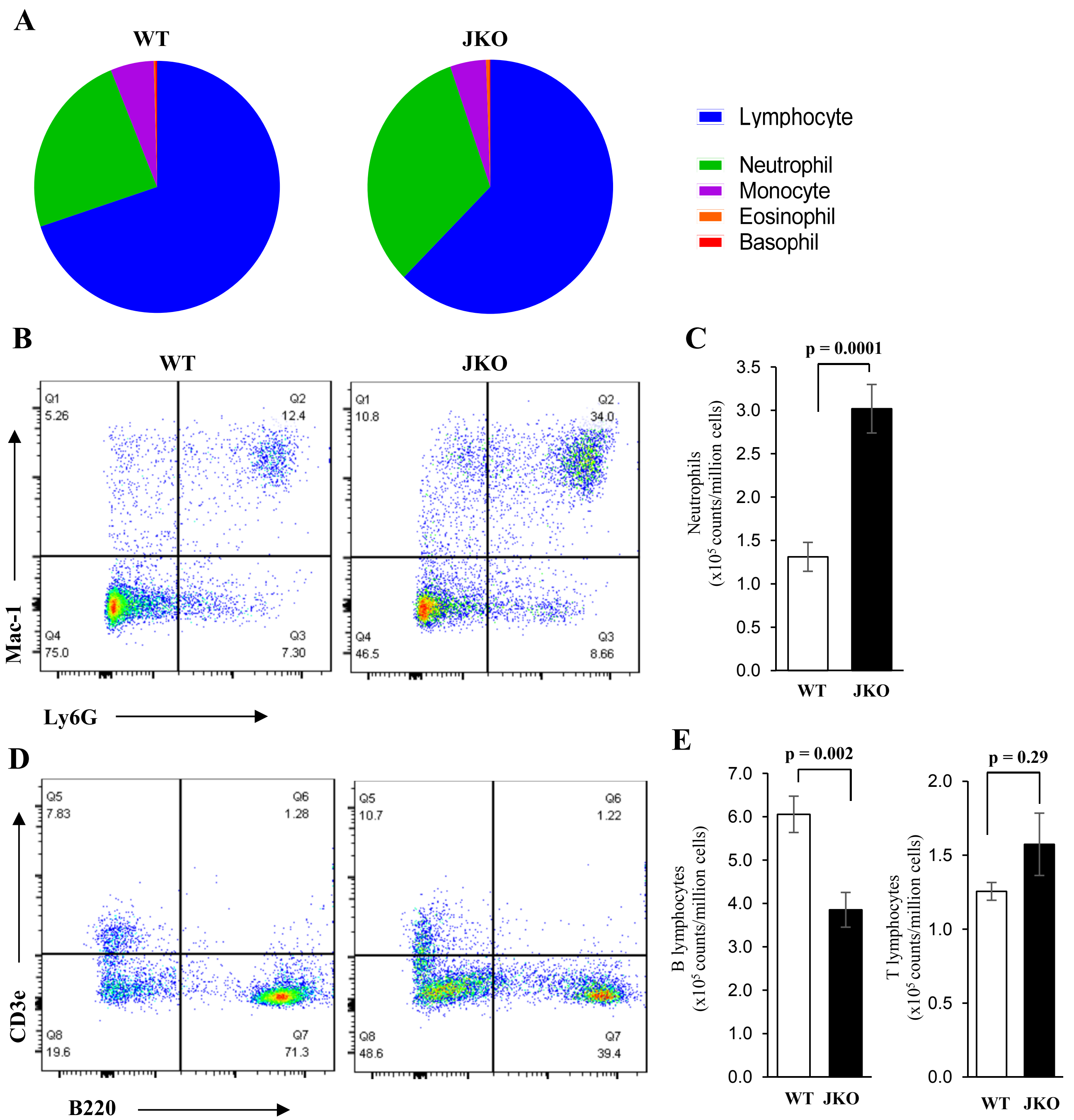

Supplemental Figure S5: Different blood cell populations in WT and JMJD1B-/- (JKO) mice. Related to Figure 7. (**A**) Complete blood count analysis and percentage distribution of lymphocytes, neutrophils, monocytes, eosinophils, and basophils in peripheral blood of wild-type (WT, n = 10) and JKO mice (n =14). Representative flow cytometry analysis of (**B, C**) granulocyte population (Gr-1^+, Mac-1^+) and total number for each genotype (mean ± s.e.m., n = 9), **(D, E)** B lymphocytes ($B220^+$) and T lymphocytes ($CD3e^+$) and total number for each genotype (mean ± s.e.m., n = 9). P values were calculated using Student's t-test.

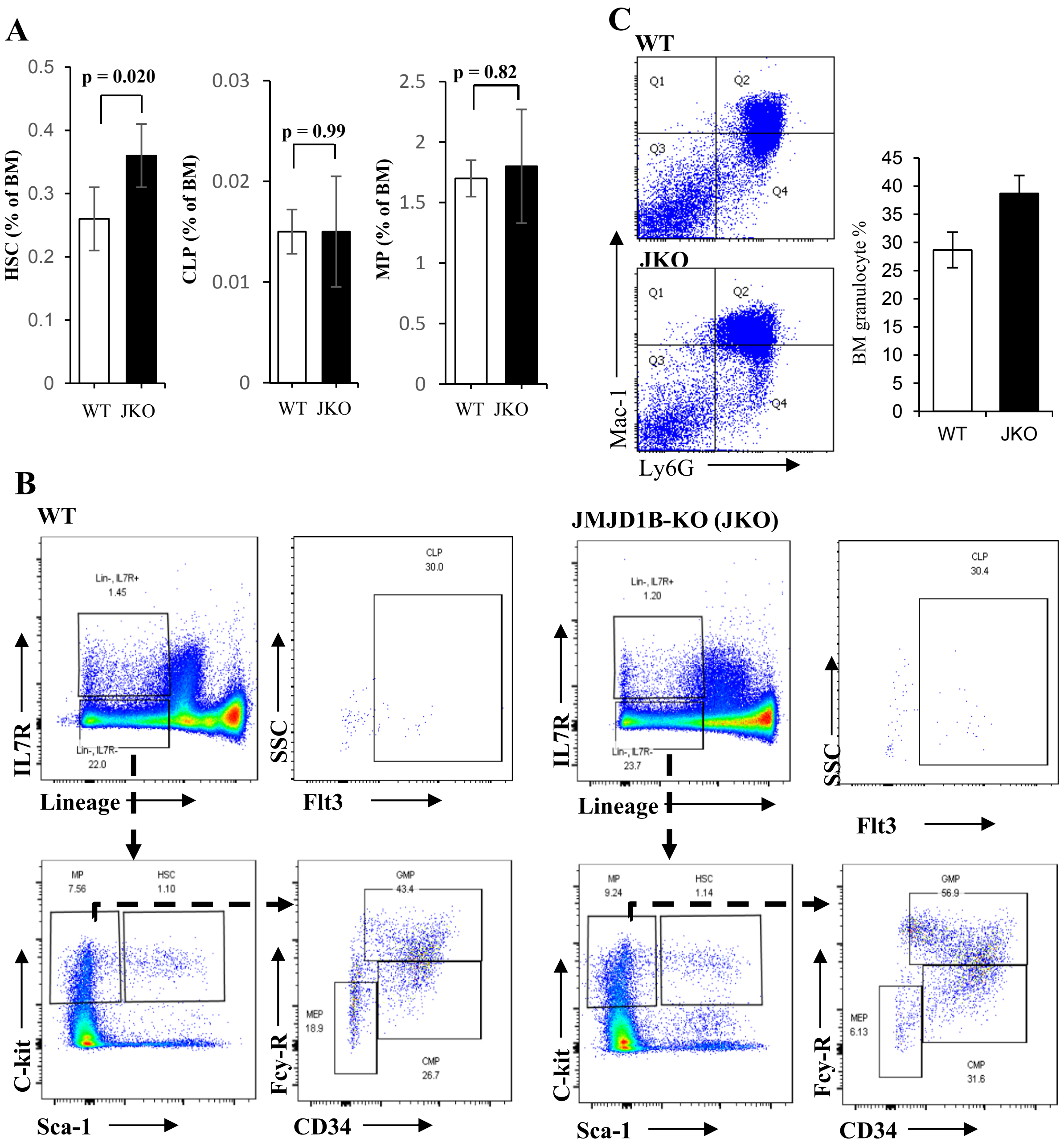

Supplemental Figure S6: Bone marrow cell population analysis by flow cytometry. Related to Figure 7. (**A**) Percentages of HSC and CLP (common lymphoid progenitor) and MP (myeloid progenitor) relative to input bone marrow cells. HSC are shown as $Lin^{-}c\text{-}Kit^{+}Sca\text{-}1^{+}$ and CLP as Lin^{-} $c\text{-}Kit^{+}Sca\text{-}1^{+}IL7Ra^{+}$ cell populations by flow cytometry. Values are means ± s.e.m of five biological replicates. P values were calculated using Student's t-test. (**B**) Representative flow cytometry analysis of bone marrow progenitor cell population compartments of age matched (22 weeks) WT (left 4 panels) and JMJD1B-/- mice (right 4 panels). Live bone marrow cells were stained for hematopoietic stem cell/progenitor cells expression marker (C-kit, Sca-1, Fcy-R, CD34, IL7R, Flt3, and CD150) along with lineage marker (Ter119, CD3, B220, NK1.1, Gr-1 and Mac-1). For CLP analysis, lineage negative and $IL7R^{+}$ were plotted for $c\text{-}kit^{+}$ and $Sca\text{-}1^{+}$ and then Flt3 positive (upper right plot). For MP analysis, lineage negative cells were divided for C-kit positive and SCA-1 negative cells (lower left plot) and Lin^{-}, $C\text{-}kit^{+}$ population was further analyze using Fcy-R and CD34 markers (lower right plot). Common myeloid progenitor (CMP) were defined as (Lin^{-}, $C\text{-}kit^{+}$, $CD34^{+}$, $Fcy\text{-}R^{mid}$), GMP as (Lin^{-}, $C\text{-}kit^{+}$, $CD34^{+}$, $Fcy\text{-}R^{hi}$) and MEP as (Lin^{-}, $C\text{-}kit^{+}$, $CD34^{-}$, $Fcy\text{-}R^{-}$). For hematopoietic stem cell (HSC) analysis, lineage negative cells were stained for C-kit and SCA-1 positive fraction (lower left plot). (**C**) Granulocyte population ($Gr\text{-}1^{+}$, $Mac\text{-}1^{+}$) in WT and JMJD1B-/- mice (means ± s.e.m of two biological replicates)

Supplemental Experimental Procedures

Cell culture

The 293T or primary MEF cells were cultured at 37°C in a 5% CO_2 standard tissue culture incubator. Dulbecco's modified Eagle's medium (DMEM, Corning Life Sciences, Corning, NY), supplemented with 10% fetal bovine serum (FBS, GE Healthcare Life Sciences, Logan, UT) and 1% penicillin-streptomycin (Thermo Fisher Scientific, Waltham, MA), was used for all routine maintenance of culture.

JMJD1B knockout in mice

JMJD1B gene expression was disrupted via gene trap technology (Supplemental Figure S3A). Male chimeric mice generated via JMJD1B mutant embryonic stem cells (*neo*$^{+}$) were crossed with female WT mice (C56BL/6 genetic background) to transmit the mutation through the germline, producing heterozygous mice on the B6.129P2 genetic background. Heterozygous (JMJD1B$^{+/-}$) and homozygous (JMJD1B$^{-/-}$) mice were selected with PCR (Supplemental Figure S3B and S3C). Homozygous knockout of JMJD1B in mice was verified by quantitative PCR to measure the mRNA levels in mouse endothelial fibroblast (MEF) cells, as well as tissue staining and western blotting to measure protein levels. No JMJD1B RNA or protein was detected *in vivo* (Supplemental Figure S3D and 3E). For phenotype characterization, we used nQuery to estimate the minimal number of mice needed for the study and Fisher exact test to calculate the p value. WT and JMJD1B$^{-/-}$ mice were randomly selected for phenotype characterization in a blind fashion. All protocols involving animals have been approved by the Institution Animal Care and

Use Committee (IACUC) of City of Hope in compliance with the Public Health Service Policy on Use of Laboratory Animals.

In vitro methylation, demethylation, and formaldehyde release assays

Recombinant WT and mutant (H1560A/D1562A/H1689A) JMJD1B were expressed as FLAG fusions using the 3xFLAG expression vector (pEZ-M12). After expression in HEK293T cells (ATCC), the proteins were purified using M2 beads, according to the manufacturer's instructions (Sigma-Aldrich). The biotin-tagged methylated histone peptide sequences and antibody resources for western blotting analysis are listed in Supplemental Tables S1 and S2. Other antibodies used in this study are also commercially available, including anti-H4 (Abcam, Cat No: ab187521), anti-JMJD1B (Cell Signaling, Cat No: 3314), HRP-labeled Streptavidin (Beyotime, Cat No: A0303), goat anti-rabbit IgG HRP (Pierce, Cat No: 31460), and goat anti-mouse IgG HRP (Pierce, Cat No: 31430). The specificity of histone antibodies was confirmed with dot blotting analysis using synthetic histone peptides (Supplemental Figure S1B).

For the demethylation reactions, the bulk histones, methylated recombinant histone H3 and H4, or synthetic peptide substrates were incubated with purified JMJD1B (0.5 μM) in demethylation buffer in a final volume of 20 μl at 37 °C. The reaction products were assayed either by western blot analysis or by using a formaldehyde release assay.

A modified version of formaldehyde release assay (Walport et al., 2016) was conducted to analyze the kinetic parameters of JMJD1B-catalyzed H4R3me2s and H3K9me2 demethylation. Briefly, JMJD1B (0.5 μM) was incubated with varying concentrations of H4R3me2s and

H3K9me2 peptide substrates in a demethylation buffer at 37°C for 0, 10, 20, 30, 40, 50, 60 minutes. After stopping the reaction with addition of EDTA to a final concentration of 10 mM, the formaldehyde concentration in each reaction was measured using a fluorescence-based formaldehyde assay kit (Sigma, St. Louis, MO) and the Km, kcat, and kcat/Km were calculated following the previously published protocol (Walport et al., 2016).

Mass spectrometry analysis

34 μM each of synthetic H4R3me2s, H4R3me2a, or H4R3me1 or 18 μM of H3K9me2 peptide substrates (Supplemental Table S1) were incubated in the absence or presence of WT (0.5 μM) or mutant (0.5 μM) JMJD1B in a demethylation buffer. After 2 or 4 h of incubation, the reaction mixtures were desalted and lyophilized. The peptide (5 μg in 100 μl demethylation buffer), was dissolved in 1 ml of 2% acetonitrile/0.1% TFA and desalted by passing through tC18 cartridges (Waters). The cartridges were activated and equilibrated using 1 ml 80% acetonitrile/0.1% TFA, followed by 1 ml 0.1% TFA (2X). The reaction mixture was loaded onto the cartridges, then washed with 1 ml 0.1% TFA (2X). The cartridges were eluted with 1 ml 80% acetonitrile/0.1% TFA and lyophilized. The lyophilized samples were then dissolved in 50% methanol/0.1% FA and analyzed by a 9.4T Q- FT-ICR MS hybrid (Solarix, Bruker Daltonics Bremen, Germany) at the National Center of Biomedical Analysis (Beijing, China).

JMJD1B overexpression and knockdown in HEK293T cells

To overexpress JMJD1B and observe its impact on arginine demethylation of H4R3me2s within the cells, PEZ-M12-JMJD1B was transfected into HEK293T cells (ATCC) using the Polyjet transfection reagent (Signagen Laboratories), according to the manufacturer’s instruction. The

absence of contamination by bacteria, yeast, or other microorganisms in the cell culture was confirmed using a mycoplasma detection kit (Sigma). After 48 h, the cells were harvested. JMJD1B overexpression was confirmed by western blotting, and the levels of H3K9me2, H4R3me2s, H4R3me1, and total histone 3 or histone 4 were determined by western blotting.

To knock down JMJD1B and to observe the impact on arginine demethylation of H4R3me2s within cells, a shRNA fragment targeting the coding sequence of JMJD1B was generated using a pair of oligonucleotides, 5'CCGGCCCTAGTTCATCGCAACCTTTCTCGAG AAAGGTTGCGATGAACTAGGGTTTTTG3' and 5'AATTCAAAAACCCTAGT TCATCGCAACCTTTC TCGAGAAAGGTTGCGATGAACTAGGG3'. A scrambled shRNA fragment was produced using the oligonucleotides, 5’CCGGCCTAAGGTTAAGTCGCCCTCG CTCGAGCGAGGGCGACTTAACCTTAGGTTTTT3’ and 5’AATTCAAAAACCTAAG GTTAAGTCGCCCTCGCTCGAGCGA GGGCGACTTAACCTTAGG3’. The DNA fragments were cloned into the PLKO.1-EGFP plasmid. The HEK293T cells were co-transfected with the PLKO.1-shJMJD1B or PLKO.1-shScramble plasmid, together with the lentiviral packaging vectors pMD2.G (Addgene) and psPAX2 (Addgene), using the Polyjet transfection reagent (Signagen Laboratories). The absence of contamination by bacteria, yeast, or other microorganisms in the cell culture was confirmed using a mycoplasma detection kit (Sigma). After 48 h, the supernatants containing the released viral particles were collected and centrifuged at 2,500 x g to remove the cell debris. The viral particles were then added to nascent HEK293T cells and cultured overnight. The virus-infected HEK293T cells were replaced with fresh virus-free medium and cultured for 36 h. Individual clones of GFP-positive cells were selected by diluting the cells into 96-well plates at one cell per well.

Analysis and sorting of hematopoietic cells from bone marrow and peripheral blood

Complete blood count (CBC) analysis was conducted for whole blood using a Hemavet 950FS hematology analyzer (Drew Scientific, Miami Lake, FL). BMCs were harvested from femur, tibia and humerus and flow cytometry analysis of lineages in peripheral blood and progenitors in bone marrow were performed as described previously (Akashi et al., 2000). Briefly, the blood cells from WT and JMJD1B$^{-/-}$ mice were stained with fluorochrome conjugated antibodies (B220, Gr-1, Mac-1, CD3e, CD4). Similarly, BMCs from WT and JMJD1B$^{-/-}$ mice were stained with fluorochrome conjugated antibodies (C-kit, SCA-1, CD34, Flt3, Fcγ-Rα, IL7Ra, CD150, Biotin-lineage). The stained cells were analyzed using a BD LSRFortessa (BD Biosciences, San Jose, CA). Antibodies specific for the following surface antigens were purchased from BD Bioscience: B220 (RA3-6B2), Mac-1 (M1/70), Gr-1 (RB6-8C5), CD4 (GK1.5), CD3e (145-2C11), IL7Rα (SB/199), Sca-1 (D7), TER119(TER-119), CD34 (RAM34), Flt3 (A2F10.1), c-Kit (2B8) FcγRII/III (2.4G2), CD150 (Q38.480), Biotin-Ter119 (TER-119), Biotin-CD3e (145-2C11), Biotin Gr-1 (RB6-8C5), Biotin-Mac-1 (M1/70), Biotin-B220 (RA3-6B2) and Biotin-NK1.1 (PK136).

Hematopoietic stem cell/progenitor/mature populations were sorted based on cell surface marker. Granulocyte-monocyte progenitors were sorted as (Lin$^-$, C-kit$^+$, CD34$^+$, Fcy-R^{hi}), common myeloid progenitors as (Lin$^-$, C-kit$^+$, CD34$^+$, Fcy-R^{mid}), megakaryocyte-erythrocytes progenitors as (Lin$^-$, C-kit$^+$, CD34$^-$, Fcy-R$^-$), hematopoietic stem cell (Lin$^-$, SCA-1$^+$, C-kit$^+$), common lymphoid progenitors as (Lin$^-$, IL7Ra$^+$, c-Kit$^+$, Sca-1$^+$,Flt3$^+$). Mature granulocyte cells populations from both blood and bone marrow were sorted as Gr-1$^+$ cells. B lymphocytes and T

lymphocytes were sorted as $B220^+$ and $CD3e^+$ cell populations, respectively. If the purity of the sorted population did not reach more than 90%, the sorting was repeated.

RNA-Seq

Sequencing libraries were prepared with the TruSeq RNA Sample Prep Kit V2 (Illumina; San Diego, CA) according to the manufacturer's instruction. Briefly, poly(A) mRNA was enriched using oligo dT magnetic beads, and reverse transcribed. The resulting double-stranded cDNAs were sheared with Covaris S220 (Covaris, Spalding, UK) with the 200 bp peak, 50 μl volume setting. The fragmented cDNA underwent end repair, 3′ end adenylation. Finally, the bar-coded adapters (Illumina) were ligated to the cDNA fragments, and 10 cycles of PCR were performed to produce the final sequencing library. Library templates were prepared for sequencing using the cBot cluster generation system with the HiSeq SR Cluster Kit V4 (Illumina, San Diego, CA). The sequencing run was performed in single read mode for 51 cycles of read 1 and 7 cycles of index read using the HiSeq 2500 platform with the HiSeq SBS Kit V4 (Illumina, San Diego, CA). The real-time analysis (RTA) 2.2.38 software package was used to process the image analysis and base calling. Raw sequence reads were mapped to the mouse genome (mm9) using TopHat v2.0.8b, and the frequency of Refseq genes was counted using HTSeq-0.6.1

ChIP-Seq

ChIP on native chromatin from HSPCs and BMCs was performed following previously published protocols (Barski et al., 2007; Cuddapah et al., 2009; Girardot et al., 2014) with the following modifications using ChIP-grade Magna protein A/G beads (Cat. No: 16-663, EMB

Millipore) and ChIP-grade antibodies against H4R3me2s (Active motif 61187), H3K9me2 (Abcam, ab1220), and histone H4 (Abcam, ab10158). BMCs were isolated from age-matched WT or JMJD1B$^{-/-}$ littermates (n = 4 for each background, 20-24 weeks). Total BMCs and isolated HSPCs were pooled together and nuclei were isolated. The isolated nuclei were incubated for 15 min at 37°C with 4 units/µl of Micrococcal nuclease (M0247, New England Biolabs) to produce >90% mononucleosomes and a small portion of di-nucleosome, as verified by agarose gel electrophoresis. Nucleosomes were precipitated with control IgG or a specific histone antibody. ChIP on histone H4 was used as the reference for total nucleosomes. ChIPed or input DNA was purified with Ampure XP beads (Cat No: A63880, Beckman Coulter).

The input DNA fragments and H4R3me2s, H3K9me2, or histone H4 ChIPed DNA fragments from each sample were barcoded and sequenced on the Illumina HiSeq 2500 to produce 51 bp reads. Reads were based-called using standard Illumina software and aligned to mouse reference genome (mm9) using NovoAlign (http://www.novocraft.com/) (Supplemental Table S3). The enrichment of H4R3me2s and H3K9me2 across the genome was analyzed by MACS2 program using p value 0.001 to identify peaks as previously described (Zhang et al., 2008). The enrichment of H4R3me2s or H3K9me2 relative to histone H4 [log 2(ChIP/ChIP H4)] or relative to the input [Log2(ChIP/input)] at the promoter regions throughout the genome was calculated. Briefly, the reads in the window from 1kb upstream of the TSS to the TSS were counted and normalized with the total uniquely mapped reads. The normalized ChIP H4R3me2 or H3K9me2 was divided by the normalized H4 reads or the normalized input reads and log2FC was calculated for each gene. A pseudo read was added to each window to avoid abnormally high normalization. In both cases, RefSeq genes (mm9) was used for the gene annotation (Quinlan

and Hall, 2010). The same approach was used to calculate the Log2FC(ChIP JMJD1B/Input) and Log2FC(ChIP PRMT5/Input).

Real-time quatitiative-PCR

For mRNA level measurement, the total RNA was extracted using the RNeasy micro Kit (Qiagen, Valencia, CA). The cDNA was synthesized from 2 μg of RNA using SuperScript III RT (Thermo Fisher Scientific). Quantitative real time PCR was performed with Power SYBER Green Master Mix (Applied Biosystems) on a Bio-Rad CFX96 real time system (Bio-Rad, Hercules, CA). ATCB was used as housekeeping loading controls to normalize gene expression using the ΔΔCt method.

For ChIP-qPCR, the input genomic DNA or ChIPed DNA was used as a template. Quantitative real time PCR was performed with Power SYBER Green Master Mix (Applied Biosystems) on a Bio-Rad CFX96 real time system (Bio-Rad, Hercules, CA). The input DNA or ChIPed H4 was used as a normalization control.

Western blot

Cells were lysed in whole cell extraction buffer (50 mM Tris pH 7.5, 150 mM NaCl, 0.5% NP-40) containing protease inhibitor cocktails (Cat#: 11873580001, Sigma). The protein concentration of each sample was quantified using the Bio-Rad protein assay reagent (Cat#: 5000006, Bio-Rad). For detection of histone and histone modifications, total histones were isolated using the Histone Extraction Kit (Cat#: ab113476, Abcam). Equal amounts of whole cell extracts or total histones were resolved by 4-15% or 15% SDS-PAGE and immunoblotted with

specified antibodies. To detect the loading control including β-actin, GAPDH, histone H3, or histone H4, the blot was stripped and reprobed with an indicated antibody.

Resources Table

REAGENT or RESOURCE	SOURCE	IDENTIFIER
Antibodies		
Mouse monoclonal anti-H3K9me2	Abcam	Cat#: Ab1220<br>RRID: AB_449854
Mouse monoclonal anti-H3K4me2	Active Motif	Cat#: 39679<br>RRID:n/a
Rabbit polyclonal anti- H3R2me2s	Active Motif	Cat#: 39703<br>RRID:n/a
Rabbit polyclonal anti- H3R2me2a	Abcam	Cat#: Ab80075<br>RRID: AB_1603562
Rabbit polyclonal anti- H3R2me1	Abcam	Cat# Ab15584<br>RRID: AB_880446
Rabbit polyclonal anti- H3R8me2s	Abcam	Cat# Ab130740<br>RRID:n/a
Rabbit polyclonal anti- H3R8me1	Active Motif	Cat#: 39673<br>RRID:n/a
Rabbit polyclonal anti- H3R17me2a	Active Motif	Cat#: 39709<br>RRID:n/a
Rabbit polyclonal anti- H3R26 me2a	EMD Millipore	Cat#: 07-215<br>RRID: AB_310435
Rabbit polyclonal anti- H4R3me1	Abcam	Cat#: Ab17339<br>RRID: AB_873863
Rabbit polyclonal anti- H4R3me2s	Active Motif	Cat#:61187<br>RRID:n/a
Rabbit polyclonal anti- H4R3me2a	Active Motif	Cat#:39706<br>RRID:n/a
Rabbit polyclonal anti- H3	Abcam	Cat#:Ab1791<br>RRID: AB_302613
Rabbit polyclonal anti- H4	Abcam	Cat#:Ab10158<br>RRID: AB_296888
BUV395 Rat Anti-Mouse CD117	BD Biosciences	Cat#: 564011<br>RRID: n/a
APC-Cy™7 Rat Anti-Mouse Ly-6A/E	BD Biosciences	Cat#: 560654<br>RRID: AB_1727552
Alexa Fluor® 647 Rat anti-Mouse CD34	BD Biosciences	Cat#:560230<br>RRID: AB_1645200
PE Rat Anti-Mouse CD135	BD Biosciences	Cat#: 553842<br>RRID: AB_395079
PE-Cy™7 Rat Anti-Mouse CD16/CD32	BD Biosciences	Cat#: 560829<br>RRID: AB_10563207
BV711 Rat Anti-Mouse CD127	BD Biosciences	Cat#: 565490<br>RRID: n/a
BV421 Rat anti-Mouse CD150	BD Biosciences	Cat#: 562811<br>RRID: n/a

FITC Hamster Anti-Mouse CD48	BD Biosciences	Cat#: 557484<br>RRID: AB_396724
Biotin Rat Anti-Mouse TER-119/Erythroid Cells	BD Biosciences	Cat#: 553672<br>RRID: AB_394985
Biotin Hamster Anti-Mouse CD3e	BD Biosciences	Cat#: 553059<br>RRID: AB_394592
Biotin Rat Anti-Mouse Ly-6G and Ly-6C	BD Biosciences	Cat#: 553124<br>RRID: AB_394640
Biotin Rat Anti-Mouse CD45R/B220	BD Biosciences	Cat#: 553085<br>RRID: AB_394615
Biotin Rat Anti-CD11b	BD Biosciences	Cat#: 557395<br>RRID: AB_2296385
Biotin Mouse Anti-Mouse NK-1.1	BD Biosciences	Cat#: 553163<br>RRID: AB_394675
PE-CF594 Streptavidin	BD Biosciences	Cat#: 562284<br>RRID: AB_11154598
FITC Rat Anti-Mouse Ly-6G and LY-6C	BD Biosciences	Cat#: 553126<br>RRID: AB_394642
BV421 Rat Anti-CD11b	BD Biosciences	Cat#: 562605<br>RRID: AB_11152949
BUV395 Hamster Anti-Mouse CD3e	BD Biosciences	Cat#: 563565<br>RRID: n/a
BV711 Rat Anti-Mouse CD4	BD Biosciences	Cat#: 563050<br>RRID: n/a
PE-Cy™7 Rat Anti-Mouse CD45R/B220	BD Biosciences	Cat#: 552772<br>RRID: AB_394458
Alexa Fluor® 488 anti-mouse CD117 (c-Kit)	BioLegend	Cat#: 105815<br>RRID: AB_493473
Chemicals, Peptides, and Recombinant Proteins		
Recombinant protein human JMJD1B	Active motif	Cat#: 31429<br>RRID:
Propidium iodide	SIGMA-ALDRICH	Cat#: 11348639001<br>RRID: n/a
DAPI	SIGMA-ALDRICH	Cat#: D9542<br>RRID: n/a
Power SYBR™ Green PCR Master Mix	Thermo Fisher Scientific	Cat#: 4367659<br>RRID: n/a
Applied Biosystems™ TaqMan™ Reverse Transcription Reagents	Applied Biosystems	Cat#: N8080234<br>RRID: n/a
Critical Commercial Assays		
Direct Lineage Cell Depletion Kit, mouse	Miltenyi Biotech	Cat#: 130-110-470<br>RRID: n/a
Deposited Data		
Raw RNA-seq and ChIP-seq data	This paper	GSE94966
Experimental Models: Cell Lines		
293T cells	ATCC	CRL-3216
Experimental Models: Organisms/Strains		
JMJD1B knockout mice B6.129P2 background	This paper	n/a
Oligonucleotides		

shRNA against JMJD1B	Sigma	Cat#:<br>TRCN0000017093<br>RRID: n/a
Scramble shRNA	Sigma	Cat# SHC001<br>RRID: n/a
Recombinant DNA		
pEZ-M12-JMJD1B	This paper	n/a

Supplemental References

Akashi, K., Traver, D., Miyamoto, T., and Weissman, I.L. (2000). A clonogenic common myeloid progenitor that gives rise to all myeloid lineages. Nature *404*, 193-197.

Barski, A., Cuddapah, S., Cui, K., Roh, T.Y., Schones, D.E., Wang, Z., Wei, G., Chepelev, I., and Zhao, K. (2007). High-resolution profiling of histone methylations in the human genome. Cell *129*, 823-837.

Cuddapah, S., Barski, A., Cui, K., Schones, D.E., Wang, Z., Wei, G., and Zhao, K. (2009). Native chromatin preparation and Illumina/Solexa library construction. Cold Spring Harbor protocols *2009*, pdb prot5237.

Girardot, M., Hirasawa, R., Kacem, S., Fritsch, L., Pontis, J., Kota, S.K., Filipponi, D., Fabbrizio, E., Sardet, C., Lohmann, F., *et al.* (2014). PRMT5-mediated histone H4 arginine-3 symmetrical dimethylation marks chromatin at G + C-rich regions of the mouse genome. Nucleic acids research *42*, 235-248.

Quinlan, A.R., and Hall, I.M. (2010). BEDTools: a flexible suite of utilities for comparing genomic features. Bioinformatics *26*, 841-842.

Walport, L.J., Hopkinson, R.J., Chowdhury, R., Schiller, R., Ge, W., Kawamura, A., and Schofield, C.J. (2016). Arginine demethylation is catalysed by a subset of JmjC histone lysine demethylases. Nature communications *7*, 11974.

Zhang, Y., Liu, T., Meyer, C.A., Eeckhoute, J., Johnson, D.S., Bernstein, B.E., Nusbaum, C., Myers, R.M., Brown, M., Li, W., *et al.* (2008). Model-based analysis of ChIP-Seq (MACS). Genome biology *9*, R137.
